# Asymmetric Ligand Field Effects in Electron‐Rich Heterometallic Extended Metal Atom Chain Compounds

**DOI:** 10.1002/chem.202502090

**Published:** 2025-10-22

**Authors:** Rebecca K. Walde, Trey C. Pankratz, Amelia M. Wheaton, Milton Acosta, John F. Berry

**Affiliations:** ^1^ Department of Chemistry University of Wisconsin–Madison 1101 University Ave Madison WI 53706 USA

**Keywords:** bond theory, chain structures, metal‐metal interactions, multiple bonds, transition metals

## Abstract

In heterometallic systems, electron donation from the ligands may influence the metal atoms in either a symmetric or asymmetric way, with the expected case being that the more electronegative metal is favored. Here, we describe a systematic study of heterometallic compounds where this expectation is not observed. In this study, we use a modification of the symmetric 2,2′‐dipyridylamine (dpa) ligand with electron donating ethyl groups, the 4,4′‐diethyl‐2,2′‐dipyridylamine ligand (dedpa), to prepare heterometallic extended metal atom chain (HEMAC) complexes with formula Mo_2_M′(dedpa)_4_Cl_2_ (M′ = Cr, Mn, Fe, Co, Ni). The effects of the electron donating substituents were studied through techniques including crystallography, magnetometry, cyclic voltammetry, EPR, Mössbauer, electronic absorption spectroscopy, and DFT calculations. We find that the new HEMACs are indeed more electron rich, easier to oxidize, and, most interestingly, the impact of the ethyl substituents is not applied equally to all the metals in the chain. The ligand field is stronger at the Mo_2_ site, but is surprisingly weaker at the M′ center when compared to Mo_2_M′(dpa)_4_Cl_2_ complexes. We also find that changing the ligand field allows for previously unassigned electronic transitions to become visible, including excitations tentatively assigned to a triplet δ‐δ* state within the Mo_2_ unit.

## Graphical abstract

Synthesis of multimetallic compounds with modifiable scaffolding ligands raises questions about the distribution of electronic effects in heterometallic systems. Here we investigate the asymmetry of the substituent effects in a modified 2,2′‐dipyradylamine ligand applied to a heterotrimetallic extended metal atom chain (HEMAC) framework. We find that the Mo_2_ core experiences a stronger effect than the heterometal, counter to expectations based on metal atom electronegativity.

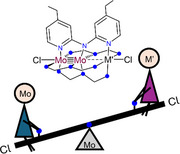

## 1 Introduction

Metal‐metal bonded complexes have long been of interest for their unique properties and reactivity.^[^ ^1^, ^2^ ^]^ The simplest of these systems, homo‐bimetallic complexes, have been well studied for many of the transition metals.^[^ ^3^, ^4^, ^5^ ^]^ While many studies focus on metal identity as the primary driver of reactivity and electronic properties (a factor whose importance certainly cannot be denied), attention must also be given to the ligands that support the metal‐metal bonds. Many bimetallic and larger clusters rely on easily modifiable ligands such as carboxylates,^[^ ^6^, ^7^, ^8^, ^9^, ^10^, ^11^, ^12^, ^13^ ^]^ NHCs,^[^ ^14^ ^]^ pincer ligands,^[^ ^15^ ^]^ nitrogen containing heterocycles,^[^ ^4^, ^14^ ^]^ and many more.^[^ ^16^, ^17^, ^18^, ^19^ ^]^ As should be expected, these supporting ligands can have a large impact on the electronics of the resulting compound. However, studies on the impact of ligands with electron donating/withdrawing substituents have focused primarily on homometallic metal‐metal bonded systems instead of heterometallic ones.^[^ ^4^, ^14^ ^]^

**Figure 1:**
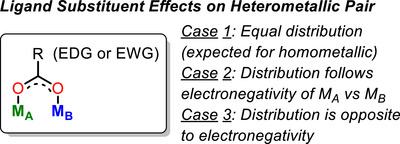
Possible outcomes for ligand substituent effects on an idealized heterometallic pair.

Consider, as a hypothetical example, a heterobimetallic system with a set of symmetric bridging ligands holding the core together. There are three possible scenarios for what can happen if a bridging ligand is modified to include an electron withdrawing or, as described in this work, electron donating substituent (Figure 1). In the first case, the impact of the ligand substituent on the two metals is symmetric, as would be expected if the complex were symmetric and homobimetallic. Several explicit studies of linear free energy relationships on metal‐metal bonded homobimetallic complexes of this type have been examined by Ren and coworkers.^[^ ^20^ ^]^ For heterobimetallic complexes, a second possibility arises in which a ligand substituent effect is distributed unequally among the two metals, with the distribution being mainly based on the differing electronegativities of the metals themselves. Examples of this case appear mainly in theoretical works,^[^ ^21^ ^]^ as most of the studies on substituent effects in heterobimetallic systems use asymmetric ligands that make assessing the impact on both metals difficult.^[^ ^22^, ^23^, ^24^, ^25^ ^]^ It is also possible that a ligand substituent effect could be distributed among two metals in such a way that is counter to what is expected from the electronegativities of the metals. It is this unusual third case that we describe in this work.

**Figure 2:**
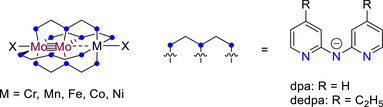
Diagram of Mo Mo–M compounds and structure of the dpa and dedpa ligands.

To interrogate the ligand substituent effects in a systematic series of well‐defined heterometallic compounds, we examine here a class of heterotrimetallic complexes that we have found to be highly tuneable and robust to electronic modification: Heterotrimetallic metal atom chain complexes (HEMACs) with a general formula Mo_2_M′(dpa)_4_Cl_2_, where M′ is varied systematically across the first row transition series (Cr, Mn, Fe, Co, or Ni), and dpa is the anion of the 2,2′‐dipyridylamine ligand (Figure 2). HEMACs of this type feature a 3‐center‐3‐electron (3*c*/3*e*) sigma bonding manifold that allows for communication and electron delocalization through the metal atom core.^[^ ^26^, ^27^ ^]^ Recently, we have found that the polarity of the Mo–M′ bonds in these compounds is opposite of that expected from the metal atoms’ electronegativities.^[^ ^27^ ^]^ This happens because the strong Mo≡Mo quadruple bond gives rise to a low energy Mo_2_ bonding combination of σ‐symmetry orbitals that effectively acts as an electron donor to M′. By adding electron donating substituents to the dpa ligand and then varying the identity of the metal atom, we can determine to what extent the umpolung of the metal‐metal bond polarity influences how the substituent effect is distributed on the heterometallic core.

For this investigation, we utilize the modified 4,4′‐diethyl‐2,2′‐dipyridylamine (dedpa) ligand, which adds electron donating ethyl substituents to the dpa ligand (Figure 2). This ligand has been used previously in the synthesis of homometallic EMAC (extended metal atom chain) complexes with Cr_3_, Co_3_, and Ni_3_ cores and has been shown to increase their solubility in nonpolar solvents and make them more electron‐rich relative to dpa compounds.^[^ ^28^, ^29^ ^]^ To probe the electron donating effects on the individual metals, we are able to use electrochemical measurements and magnetic measurements as probes of the relative HOMO energies of the HEMACs and ligand field splitting of M′.

**Scheme 1:**
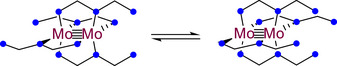
Isomerism of the ligands of **1** and **7** from the crystallographically characterized trans‐(2,2) geometry to the (4,0) geometry found in HEMACs.

In this work we present a modernized synthesis method for the Hdedpa ligand using Buckwald‐Hartwig cross coupling techniques^[^ ^30^, ^31^ ^]^ that has allowed us to synthesize the novel metalloligand Mo_2_(dedpa)_4_ (**1**). Metalloligand **1**, as expected, is vastly more soluble in common organic solvents than Mo_2_(dpa)_4_ (**7**), as well as being more electron rich. We have used **1** to synthesize a novel series of Mo_2_M′(dedpa)_4_Cl_2_ HEMAC complexes with M′ = Cr (**2**), Mn (**3**), Fe (**4**), Co (**5**), and Ni (**6**) using a solution state synthesis method with 1,4‐dioxane as a solvent that we demonstrate works well for both the dpa and dedpa HEMACs. Finally, we use computational methods to explore how the ethyl substituents on dedpa impact ligand “shuffling” (Scheme 1) of **1** and **7** (a process that appears to be facile for **1**, and is a necessary step to form HEMAC complexes from either metalloligand).

## 2 Results and Discussion

### 2.1 Synthesis

**Scheme 2:**
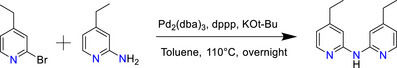
Synthesis of the Hdedpa ligand used in this work. Pd_2_(dba)_3_ refers to tris(dibenzylideneacetone)dipalladium(0), and dppp refers to 1,3‐bis(diphenylphosphino)propane.

The synthesis of the 4,4′‐diethyl‐2,2′‐dipyridylamine (Hdedpa) ligand was achieved here through a modified Buchwald‐Hartwig reaction (Scheme 2).^[^ ^31^ ^]^ This method is higher yielding (60% vs. 25% reported) than previously reported syntheses^[^ ^29^ ^]^ and compatible with column‐free workup. Deprotonation of Hdedpa with potassium bis(trimethylsilyl)amide yields the salt (Kdedpa), which was combined without further purification with Mo_2_(OAc)_4_ in THF to produce Mo_2_(dedpa)_4_ (**1**) in excellent yield (>85%).

**Figure 3:**
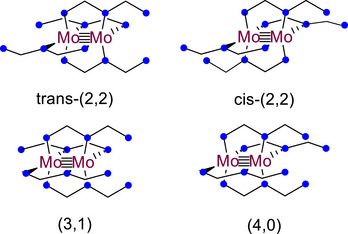
Possible conformations of the ligands around **1** in solution.

Examination of **1** by NMR spectroscopy at room temperature and ‐20 °C in CD_2_Cl_2_ reveals a complex, temperature dependent equilibrium in the solution state. Further investigation with 2D NMR methods shows multiple sets of pyridine rings that appear to interchange with each other. We observe that there are three major groups of peaks and four minor groups. Multiple conformations of the ligands around the Mo_2_ core of **1** are possible (Figure 3), leading us to believe that the speciation observed in the NMR spectra is the result of a slow equilibrium (slow relative to the NMR timescale) between ligand conformations in the solution state. A workup of the data is given in the Supporting Information (Figures S7–S21), though definitive assignment of the species observed in solution is not possible. While the dpa analog Mo_2_(dpa)_4_, **7**, is rigorously insoluble in most solvents, **1**, by contrast, is soluble in a wide range of solvents spanning in polarity from hexanes to MeOH.

**Scheme 3:**
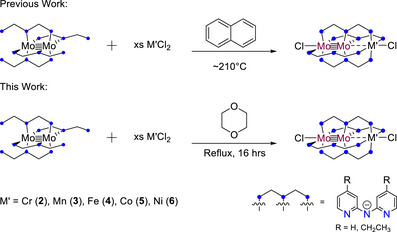
General reaction schemes for producing HEMAC complexes from either naphthalene or dioxane as solvents. In previous works, the naphthalene method was the only method available for the production of Mo_2_M'dpa_4_Cl_2_ HEMACs.

To produce Mo_2_M′(depa)_4_Cl_2_ HEMACs with M′ = Cr (**2**), Mn (**3**), Fe (**4**), Co (**5**), and Ni (**6**), two methods of synthesis were explored, as outlined in Scheme 3. First, we attempted the same method employed for the synthesis of Mo_2_M′(dpa)_4_Cl_2_ compounds previously, using molten naphthalene as solvent.^[^ ^32^, ^33^, ^34^, ^35^, ^36^ ^]^ Yields from these reactions ranged from fair (∼30%) to excellent (∼85%) depending on the metal used (Table 1).

**Table 1:** Yields for HEMAC complexes from naphthalene or dioxane synthesis.

Complex	Naphthalene Yield %	Dioxane Yield %
Mo_2_Cr(dpa)_4_Cl_2_	45	28
Mo_2_Mn(dpa)_4_Cl_2_	32	25
Mo_2_Fe(dpa)_4_Cl_2_	43	74
Mo_2_Co(dpa)_4_Cl_2_	67	63
Mo_2_Ni(dpa)_4_Cl_2_	39	93
Mo_2_Cr(dedpa)_4_Cl_2_ (**2**)	60	17
Mo_2_Mn(dedpa)_4_Cl_2_ (**3**)	46	45
Mo_2_Fe(dedpa)_4_Cl_2_ (**4**)	85	50^[^ ^a^ ^]^
Mo_2_Co(dedpa)_4_Cl_2_ (**5**)	30	65
Mo_2_Ni(dedpa)_4_Cl_2_ (**6**)	56	75

^[a]^ The yield improves to 87% when 10 eq LiCl is included in the reaction mixture.

The second method that we used to generate HEMAC complexes was prompted by the increased solubility of **1** as compared to **7**. First, we investigated the solution state method used to synthesize HEMACs with formula Cr_2_M′dpa_4_Cl_2_: using THF as a solvent.^[^ ^34^, ^36^, ^37^ ^]^ This method failed to produce the desired product, so a higher boiling solvent was chosen: 1,4‐dioxane. This ether proved to facilitate solution state synthesis for HEMACs using both the dpa and dedpa ligands. Yields for 2–6 and the dpa HEMACs are given in Table 1. For the dpa HEMACs, yields in 1,4‐dioxane for M′ = Cr, Mn, and Co are comparable to the previously reported syntheses, while the yields for M′ = Fe and Ni are improved. The results are similar for the dedpa HEMACs. Yields for **5** and **6** are improved using 1,4‐dioxane while the yield for **3** is comparable. Yields for **2** and **4** are significantly lower. The CrCl_2_ salt used in the synthesis of **2** is not very soluble in 1,4‐dioxane, likely accounting for its low yield in the solution state synthesis. For **4**, the yield can be improved from 50% to 87% by adding 10 eq of LiCl. We have not yet tested this additive in other HEMAC syntheses to determine if this effect is particular to **4** or globally applicable.

Despite the varying yields, synthesis in the solution state has a number of advantages when compared to using naphthalene as a solvent. For the dedpa HEMACs, the primary benefit is the ability to recover unreacted **1** from the reaction mixture by extraction with hexanes. We have also found workup using method 2 to be faster than method 1, though the reaction times are longer.

### 2.2 X‐Ray Crystallography

**Figure 4:**
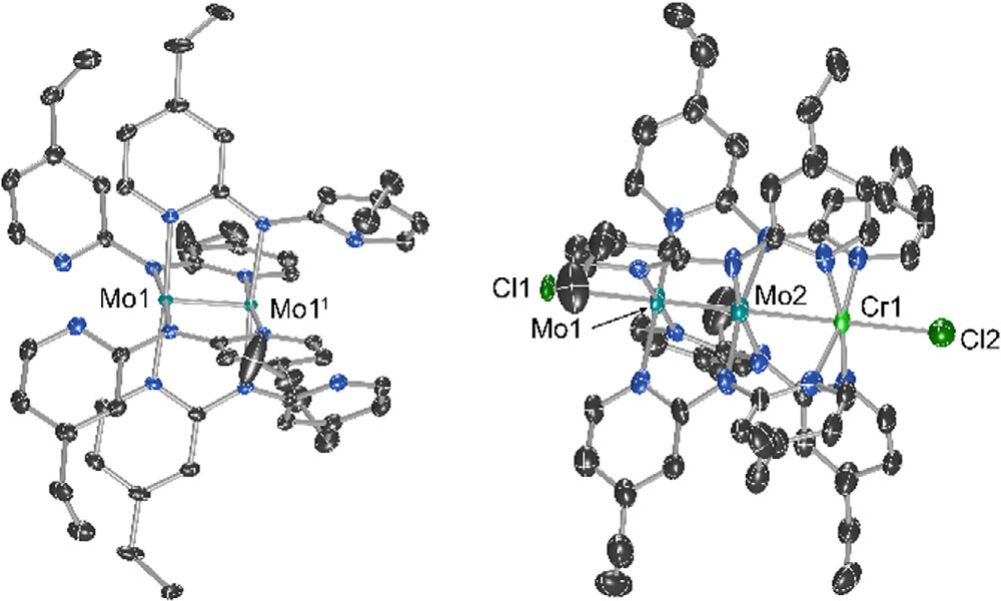
Left: molecular structure of **1**. Right: Molecular structure of **2**. Ellipsoids are drawn at 50% probability and hydrogen atoms are omitted for clarity. Where disorder is present, only the major component is shown.

Compounds **1–6** have been structurally characterized, and a full description of each structure can be found in the SI. Compound **1**, (Figure 4 left) was modelled in the *I*4_1_/*a* space group and adopts the tetragonal paddlewheel geometry expected for quadruply‐bonded Mo_2_^4+^ compounds. The molecule lies on the site of a crystallographic inversion center, which is located at the midpoint of the Mo–Mo vector; thus, half of the molecule is symmetry independent. Compound **1** was found to have a Mo≡Mo bond length of 2.09 Å, typical of a Mo≡Mo quadruple bond.^[^ ^5^ ^]^ The Mo≡Mo bond length of **1** is shorter than that of **7** by 0.03 Å. Compound **1** also has a slightly smaller N–Mo–Mo–N torsion angle than **7** (∼2° vs. ∼4°), indicating a somewhat stronger δ bonding interaction between the Mo atoms in **1**.

The structure for compound **2** is shown in Figure 4 (right), and the structures of **3**–**6** are qualitatively similar (Figures S33–40). Compounds **2**, **4**, **5**, and **6** are isomorphous in the *P*4*nc* space group whereas **3** adopts the cubic space group *P*432. Aside from **3**, the compounds crystallize without solvent; the crystal packing is instead dominated by interactions between the peripheral ethyl substituents fitting together like puzzle pieces (Figure S53).

**Figure 5:**
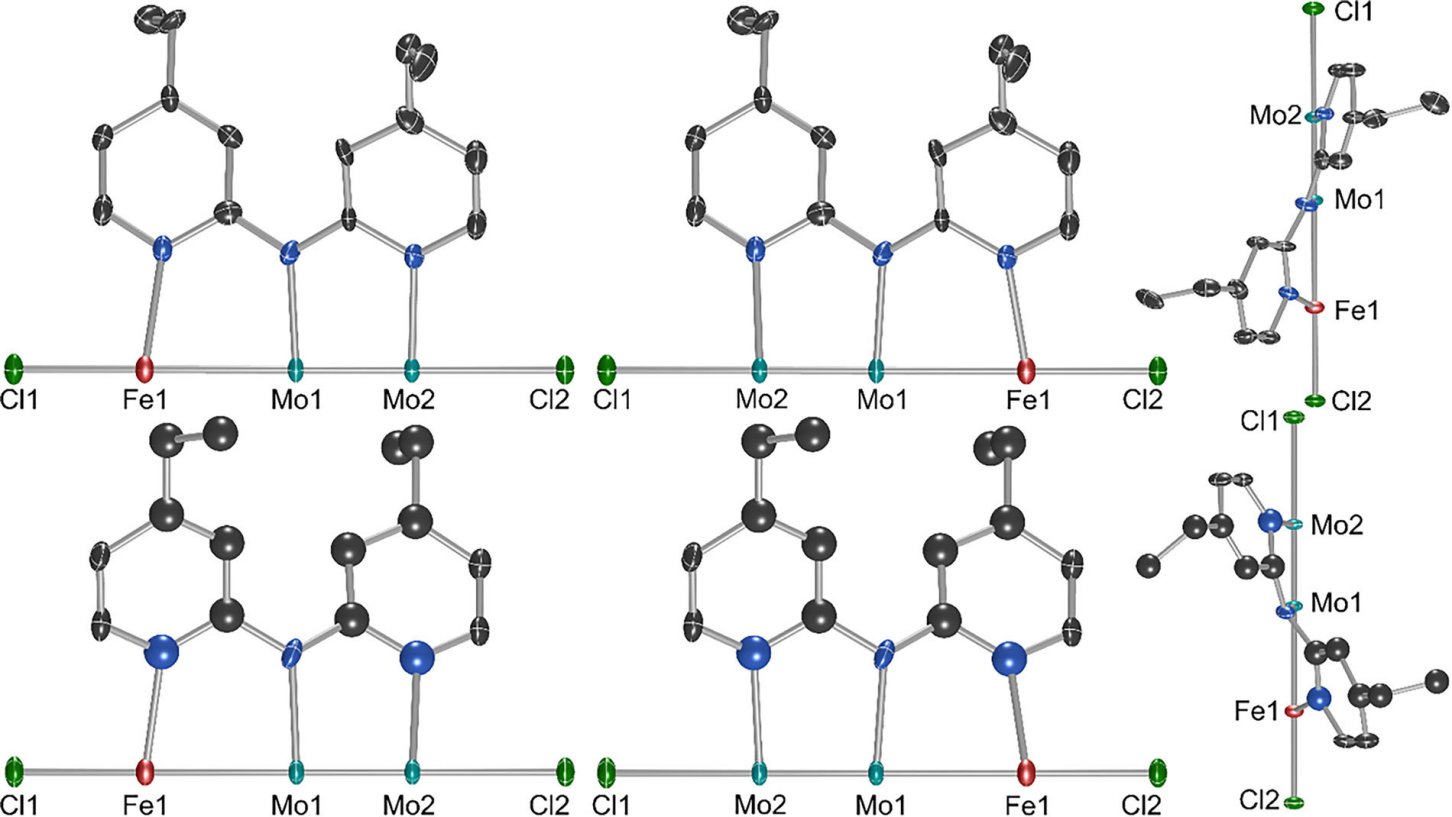
Positional disorder within the crystal structure of **4**, also present in **2**, **5**, and **6**, modeled as four distinct molecular orientations of two enantiomers. The top row shows the Λ enantiomer with both metal orientations (major left, minor right). The bottom row shows the Δ enantiomer with both orientations. The right most images show a side‐on view of the asymmetric unit to illustrate the different helical twist of the two enantiomers. Only one ligand out of **4** is shown around the metal axis for clarity. Hydrogen atoms are omitted for clarity. Ellipsoids are shown at 50% probability.

For the structures in the *P*4*nc* space group, the metal atoms in the molecule are colinear with a crystallographic fourfold axis. The structures are complicated by two types of positional disorder. First, there is disorder of the metal atom positions such that molecules pointing in opposite directions both occupy the same crystallographic site. The metal atom positions show a ∼55:45 split over the two sites in all structures. The second type of positional disorder is caused by the dedpa ligands wrapping around the metal atom chains in a helical fashion, making the trimetallic compounds chiral. Both the Λ and the Δ enantiomers of the helical structures cocrystallize at the same site, leading to positional disorder in the ligand manifold with one of the isomers (in this case, the Λ isomer) favored with an occupancy of ∼80%. These instances of positional disorder result in four distinct crystallographic orientations of the molecule at a single site that can be more simply described as pairs of enantiomers that face in opposite directions. The four disordered orientations are illustrated in Figure 5. Bond lengths for the major disorder components are provided in Table 2, with a full description of each compound found in the Supporting Information.

**Table 2:** Crystallographic and computational bond lengths of interest for the major components of 2–6.

M’	Mo‐Mo [Å]	Mo‐M' [Å]	M’‐N [Å]	Mo_in_‐N [Å]	Mo_out_‐N [Å]	Mo‐Cl [Å]	M'‐Cl [Å]
**2**	2.11(2)	2.77(2)	2.181(8)	2.123(5)	2.165(7)	2.736(17)	2.48(2)
**2‐DFT**	2.108	2.653	2.110	2.139	2.230	2.721	2.501
**3**	2.1954(19)*	2.765(4)*	2.267(4)	2.133(5)	2.211(3)	2.808(3)	2.238(4)
**3‐DFT**	2.109	2.773	2.236	2.150	2.232	2.709	2.366
**4**	2.100(9)	2.721(14)	2.206(8)	2.131(4)	2.171(3)	2.773(8)	2.369(13)
**4‐DFT**	2.114	2.689	2.169	2.133	2.213	2.620	2.318
**5**	2.122(9)	2.620(16)	2.190(8)	2.121(5)	2.183(7)	2.688(8)	2.470(13)
**5‐DFT**	2.115	2.574	2.137	2.133	2.213	2.622	2.370
**6**	2.118(9)	2.589(18)	2.151(9)	2.119(5)	2.174(8)	2.645(8)	2.435(15)
**6‐DFT**	2.112	2.511	2.096	2.129	2.214	2.601	2.408

Due to the high crystallographic symmetry for **3**, the central Mo atom of the structure is fixed at a site of 422 symmetry and does not refine to an atomic position off of this site. However, it is clear from comparing metal‐metal bond distances in the structures of **2**, **4**, **5**, and **6**, that this Mo atom in **3** *should* be at a position slightly off of the 422 site, further toward the terminal Mo atom along the fourfold axis. Since it is not possible to refine the Mo atom at a more appropriate site, bond lengths involving this Mo atom are inconsistent with those seen in the other crystal structures and are thus deemed not to be chemically reasonable. These distances, marked with * in Table 2, are excluded from further analysis and are replaced, where necessary, with the bond lengths from the computationally optimized structure.

In Table 2, we see a decrease in Mo─M′ bond lengths of ∼ 0.2 Å going from **2** to **6**. A similar trend was observed with the analogous dpa compounds (Table S2), which is attributed to the decrease in M′ radius and increase in Mo─M′ covalency with increasing electronegativity of M′.^[^ ^27^ ^]^ In addition, the M′─N bond lengths give insight into the spin state of M′. For **2–6**, the M′─N bond lengths > 2.15 Å indicate that each compound contains a high spin M′^2+^ ion. These results are consistent with the spin states found in the Mo_2_M′(dpa)_4_Cl_2_ series, despite the fact that dedpa is more electron rich than dpa and is, presumably, a stronger field ligand.^[^ ^27^, ^34^, ^38^ ^]^ In fact, the M′─N bond lengths are uniformly *longer* in **2–6** than in the analogous dpa compounds, suggesting that the dedpa ligand is actually a *poorer* σ‐donor to M′ in this series. A possible reason for this seeming paradox is that dedpa forms shorter bonds to the more electron poor outer Mo atom than dpa does, by ∼0.07–0.1 Å. Since the inner Mo─N distances are very similar for both series of compounds, there is a lever effect whereby a shorter outer Mo─N distance requires a longer M′─N distance. Overall, this leads to a weaker ligand field around the less tightly bound M′ atom and a stronger field around the more tightly bound outer Mo atom. Thus, the electron donating effect of the dedpa ligand is not distributed equally among all metal atoms in the HEMACs.

### 2.3 Magnetic Properties

**Figure 6:**
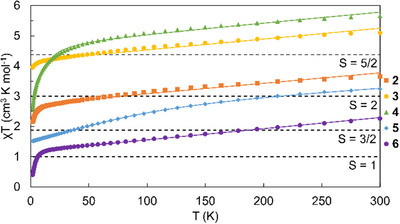
SQUID Magnetometry data for **2**–**6**. Data are shown as markers with the fits indicated with solid lines. The spin‐only expected values are given with dotted lines. For each compound, the saturation value approximates the spin only expectation value.

The high spin states of M′ in **2–6** were confirmed through SQUID magnetometry. Magnetic susceptibility data in the form of χ·T versus T plots for each compound are shown in Figure 6. For each compound except **5,** the χ·T versus T curve is characterized by a sharp rise starting at very low temperatures, then a linear increase with increasing temperature. The susceptibility for **5** shows a gradual incline at low temperatures that levels off at higher temperatures. Each compound reaches the expected spin only values for a high‐spin M′^2+^ ion in the high temperature limit, as indicated in Figure 6, confirming that the M′^2+^ ions in **2–6** are all high spin.

The magnetic susceptibility and reduced magnetization data for compounds **2–6** were modeled using spin systems with *S* = 2, 5/2, 2, 3/2, and 1, respectively, using the parameters given in Table 3. These models consider each compound to contain an isolated M′ center coordinated to a diamagnetic, quadruply‐bonded, Mo_2_ unit. The sharper rise at low temperatures seen in **2–6** indicates magnetic anisotropy that can be attributed to either an effect of zero‐field splitting (ZFS), an effect of intermolecular coupling (*zJ*), or a combination of the two. Compounds **2** and **3** are well modelled only considering axial ZFS, but **4–6** required the additional inclusion of an intermolecular term. The modelled ZFS terms for **2** and **4** have values of *D* < 0 cm^−1^, while those for **3**, **5**, and **6** have *D* > 0 cm^−1^, with the sign of *D* being determined via modeling of the reduced magnetization curves. The intermolecular terms included for **4–6** are negative in all cases, indicating antiferromagnetic coupling between molecules in the lattice. The gentle, linear rise in the higher temperature regime for all compounds was modelled as temperature independent paramagnetism (TIP), which is large, but within a narrow range of 2032 to 3,697 × 10^−6^ cm^3^ mol^−1^. The large TIP values could be attributed to one of two sources: It is possible that the relatively low‐lying triplet δ‐δ* state of the Mo_2_ unit contributes to the large TIP, or a superparamagnetic impurity could be present in the cavity of the magnetometer. Given the fact that similar dpa complexes show little to no TIP despite a (presumably) lower‐lying triplet δ‐δ* state, we find it more likely that the TIP can be attributed to impurities.

**Table 3:** Fitting parameters for SQUID magnetometry data of **2**–**6**; EPR simulation parameters for **3** and **5** are given in brackets.

M′	*g*_⊥_ [EPR]	*g*_∥_ [EPR]	*D* (cm^−1^) [EPR]	TIP	z*J*
**2·1.5 DCM**	2.012(2)	1.696(3)	−1.304(6)	0.00343(2)	N/A
**3**	1.953(2) [1.95, 2.1]	1.953(2) [2]	0.5–0.75^[^ ^a^ ^]^ [0.3]	0.00358(6)	N/A
**4**	2.607(4)	2.313(5)	−6.36(4)	0.00354(6)	−0.0063(9)
**5**	2.2362(9) [2.37, 2.89]	2.858(5) [2.3]	79.5(2) [79.5]	0.002032(9)	−0.0024(3)
**6**	2.2196(9)	2.092(2)	7.864(5)	0.003697(5)	−0.015(1)

^[a]^ Value is given as a range because the reduced magnetization and susceptibility data were modelled independently.

Compounds **2** and **4** have easy axis magnetic anisotropy (*D* < 0), presumably oriented along the Mo‐Mo‐M′ axis. The small value of *D* for **3** is consistent with the *d* ^5^ configuration for Mn(II). The resulting *g*‐factors of these models are consistent with the respective valence electron count of each M′ metal. For **2** the *g*_ave_ < 2.00 is consistent with the *d* ^4^ electron configuration of Cr(II). The average *g* for **3** is also less than 2; while the *d* ^5^ Mn(II) ion is expected to have isotropic *g* = 2.00, the lower value for **3** likely reflects some electron delocalization to the Mo(II) ions. The *g*_ave_ of **4–6** are greater than 2.00, consistent with M′ systems with greater than five valence electrons.

**Figure 7:**
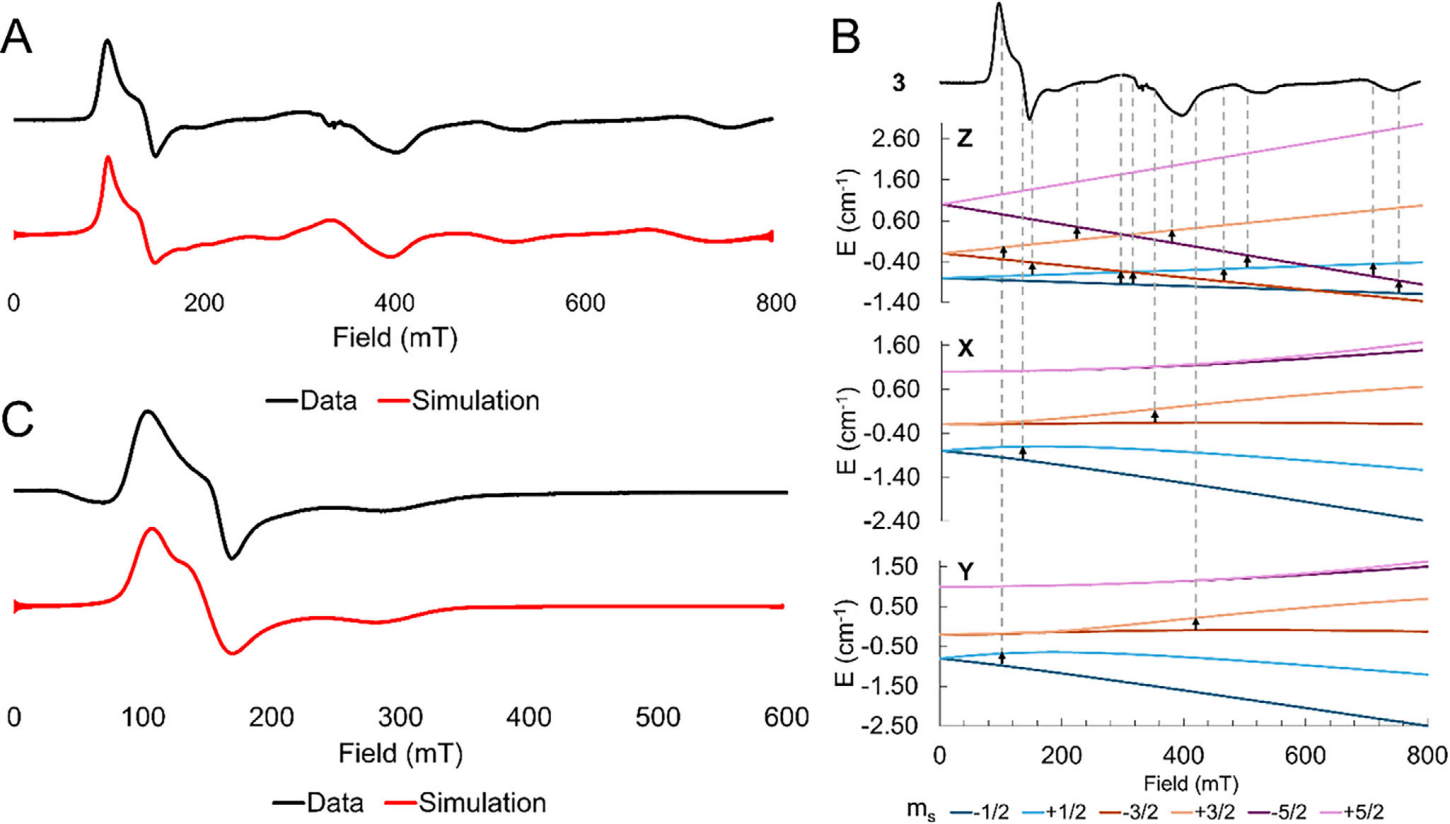
A: An EPR spectrum of **3** taken in frozen DCM solution at 5 K. The spectrum is simulated in red with g values of [1.95, 2.1, 2], an *E*/*D* of 0.03, some unresolved hyperfine from the Mn nucleus of A = [150, 50, 0] MHz, with broadening from *D*‐strain of [1500, 100] MHz. B: A Zeeman analysis of the transitions visible in the X‐band EPR spectrum of **3**. The experimental spectrum is shown at the top, with the simulated Zeeman diagrams for an applied field along the *z*, *x*, and *y* directions from top to bottom. The m_*S*_ states are given in pairs of complimentary colors, with m_*S*_ ± ½ at the bottom in blue, m_*S*_ ± 3/2 in the middle in orange, and m_*S*_ ± 5/2 in pink at the top. C: An EPR spectrum of **5** taken in frozen DCM solution at 10 K. The spectrum is simulated in red with *g*‐values of [2.37, 2.89, 2.30]. The *D*‐value used here is fixed to that from the fitted SQUID magnetometry data, at 79.5 cm^−1^. The *E*/*D* is 0.07. *A* = [80, 80, 0] MHz, H strain = [500, 1000, 1500] MHz, *D* strain = [0, 200000] MHz.

Compounds **3** and **5** were further investigated using X‐band EPR spectroscopy in frozen DCM solution at 5 and 10 K, respectively (Figure 7, A, C). Spectra for both compounds show one major, nearly axial feature (*E*/*D* = 0.03 (**3**) and 0.07 (**5**)) with intrinsic *g*_avg_ = 2.01 (**3**) and 2.52 (**5**) consistent with high‐spin Mn^2+^ and Co^2+^, respectively. Parameters used in the simulation of both spectra are given in brackets in Table 3. The EPR spectrum for **3** is complex, with features at effective *g* values of $g_{⊥}$ ∼ 6 and $g_{∥}$ ∼ 2 with several other observable features suggesting *S* = 5/2 with a small *D* value (0.3 cm^−1^) that is similar to the energy of the microwave quantum (9.4 GHz = 0.31 cm^−1^). Mo_2_Mn(dpa)_4_Cl_2_ was studied by high‐field EPR spectroscopy and found to have an essentially identical *D* value to **3**.^[^ ^36^ ^]^ Further analysis of **3** using simulated Zeeman splitting diagrams with applied fields in the *x*, *y*, and *z* directions helps to confirm the assignment of the *D* value of **3** as positive and very close to the microwave quantum with a slight rhombicity contributing to the number of visible transitions including inter‐doublet transitions (Figure 7B).

The spectrum of **5** is more clearly in the *S* = 3/2, *D* > *h*ν regime with effective *g* values of $g_{⊥}$ ∼ 4 and $g_{∥}$ ∼ 2. This spectrum was modeled using a value of *D* fixed to that determined from the magnetic susceptibility measurement and *E*/*D* of 0.07. The features are broadened, likely due to unresolved hyperfine coupling to the *I* = 7/2 ^59^Co nucleus. Mo_2_Co(dpa)_4_Br_2_ has similar EPR features to **5** in its high‐spin form: *D* > *h*ν and *E*/*D* = 0.1.^[^ ^39^ ^]^

### 2.4 Mössbauer Spectrum of **4**

**Figure 8:**
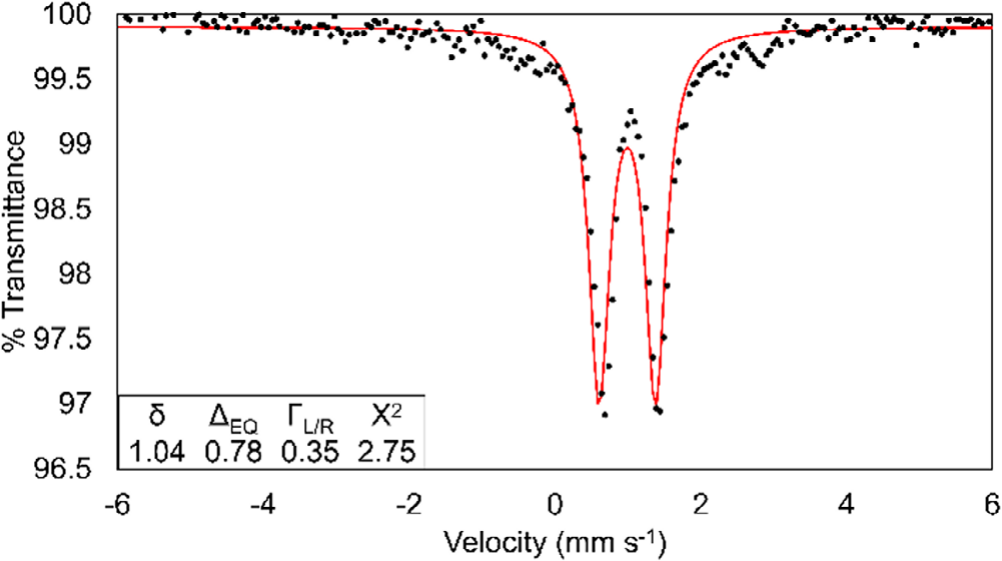
A Mössbauer spectrum of **4** taken at 77 K that shows one site of high spin Fe^2+^ with the fit shown in red. The doublet has a δ of 1.04 mm s^−1^ and ΔE_Q_ of 0.78 mm s^−1^.

The Mössbauer spectrum of **4** displays one quadrupole doublet (Figure 8) with an isomer shift (δ) of 1.04 mm s^−1^ and a quadrupole splitting (∆E_Q_) of 0.78 mm s^−1^. The ∆E_Q_ and δ for this feature are consistent with a single pseudo‐octahedral high‐spin Fe^2+^ site. Given the metal‐atom disorder in the crystal structure of **4**, two Fe sites would be expected to be visible in the Mössbauer spectrum, however, as only one quadrupole doublet is observed, it can be assumed that the two sites have very similar Mössbauer parameters. Attempts to model the doublet as a pair of overlapping doublets were unproductive. The similarity of the Mössbauer parameters for the different Fe sites in **4** is in contrast with the Mössbauer data for Cr_2_Fe(dpa)_4_Cl_2_, for which we found two distinct quadrupole doublets corresponding to two crystallographically independent Fe sites, with identical δ values (1.01 mm s^−1^) and very different ΔE_Q_ values (1.63 vs. 2.39 mm s^−1^).^[^ ^36^, ^37^ ^]^

Mössbauer parameters for **4** and related compounds are given in Table 4. In comparison to the other known CrCrFe and MoMoFe compounds, **4** has a smaller quadrupole splitting by up to 1.7 mm s^−1^. This smaller quadrupole splitting indicates a smaller electric field gradient at the Fe nucleus for **4**, which is consistent with the structural analysis above indicating longer Fe─N bonds and a weaker ligand field for the Fe atom in **4** as compared to other M_2_Fe(dpa)_4_X_2_ compounds. The isomer shift of **4** is very similar to other compounds in this family, and lies in the expected range for a high spin Fe^2+^ complex.

**Table 4:** Collected Mössbauer parameters for 4 and related compounds.

Compound	δ mm s^−1^	ΔE_Q_ mm s^−1^
Cr_2_Fe(dpa)_4_Cl_2_ (site 1)^[^ ^36^ ^]^	1.01	1.63
Cr_2_Fe(dpa)_4_Cl_2_ (site 2)^[^ ^36^ ^]^	1.01	2.39
Mo_2_Fe(dpa)_4_Cl_2_^[^ ^40^ ^]^	1.02	2.02
Mo_2_Fe(dpa)_4_(OTf)_2_^[^ ^27^ ^]^	1.05	2.48
**4**	1.04	0.78
**4** (site 1, DFT)	0.88	2.96
**4** (site 2, DFT)	0.88	2.9
**4** (DFT optimized structure)	0.82	2.92

Mössbauer parameters were calculated using DFT methods for a computationally optimized structure of **4** as well as the two crystallographically distinct sites in frozen geometry single point calculations. The experimental values of δ and ΔE_Q_ were not well reproduced using these computational methods, which is likely attributable to the partial sharing of valence electrons with the Mo atoms. Nevertheless, the predicted differences in δ and ΔE_Q_ between the two crystallographic sites are sufficiently small as to be functionally indistinguishable (Δδ, ΔΔE_Q_ ∼ 0.06 mm s^−1^), validating the theory that the two sites are too similar to appear separately in the experimental spectrum.

### 2.5 Electrochemical Properties

**Figure 9:**
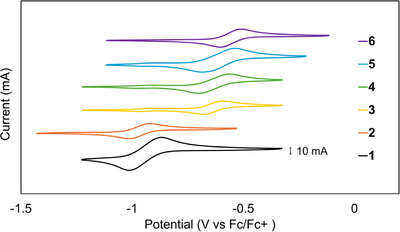
Cyclic voltammetry data of 1–6 taken in CH_2_Cl_2_ versus Fc/Fc^+^ with a scan rate of 100 mV/s. The scans of **3**–**5** show a second peak at approximately the same V as **1**, indicating a slow equilibrium relative to scan rate in solution of **3**–**6** with **1**. The scans of **3**–**6** show a trend in which the potential of the major signal (theorized to be the Mo_2_^4+/5+^ couple) shifts more positive with an increasingly electronegative heterometal.

The cyclic voltammograms (CVs) of **1–6** taken in CH_2_Cl_2_ solution are shown in Figure 9. Each compound displays one major quasi‐reversible feature; that for **1** at ‐966 mV versus Fc/Fc^+^ may be straightforwardly assigned to the Mo_2_^4+/5+^ redox couple of the quadruple‐bonded Mo_2_ unit. This redox couple has a very low voltage as compared to other Mo_2_ complexes supported by N,N′‐donor ligands. For example, Mo_2_(formamidine)_4_ complexes have Mo_2_^4+/5+^ couples ranging from ‐381 mV to +170 mV versus Fc/Fc^+^,^[^ ^41^ ^]^ and the first redox event for Mo_2_(2‐anilino‐pyridinate)_4_ appears at 0.00 V versus Ag/AgCl (∼ ‐400 mV vs. Fc/Fc^+^).^[^ ^42^ ^]^ The parent dpa compound, **7**, is insoluble and thus could not be interrogated by cyclic voltammetry. However, salts of the **7** ^+^ cation are isolable and have been studied.^[^ ^35^ ^]^ The Mo_2_^4+/5+^ couple for **7** is reported at ‐832 mV versus Fc/Fc^+^. Compound **1** is thus easier to oxidize than its dpa congener by ∼ 130 mV, which is a significant change indicating that **1** is more electron rich than **7**. This change mirrors the lowering of the M_3_^6+/7+^ couples by ∼ 130 mV in M_3_(dedpa)_4_Cl_2_ versus M_3_(dpa)_4_Cl_2_ compounds with M = Cr, Co, and Ni.^[^ ^28^, ^29^ ^]^

We report here, for the first time, electrochemical measurements on a complete series of Mo_2_M′ HEMAC compounds with M′ = Cr─Ni. Compound **2** has a low Mo_2_M′^6+/7+^ couple, ‐969 mV versus Fc/Fc^+^, whereas the corresponding redox events for **3**–**6** are more invariant, within the relatively small range of ‐635 to ‐556 mV versus Fc/Fc^+^. These data suggest that there is a fundamental difference in the nature of the redox process for **2** versus **3**–**6**. We may consider whether the redox processes in these compounds may be attributed to the Mo_2_^4+/5+^ couple or the M′^2+/3+^ couple. One may expect from first principles that the addition of a positively charged Lewis acid to a redox active Mo_2_ unit would cause the Mo_2_ unit to be more difficult to oxidize. Thus, oxidation of the Mo_2_ unit in **3**–**6** is less accessible than in **1** by at least 330 mV. This assignment of the quadruply‐bonded Mo_2_ group as the redox active unit in **3**–**6** also agrees with our recent report of the [Mo_2_Ni(dpa)_4_Cl_2_]^+^ cationic species, which clearly displayed evidence of an oxidized Mo_2_^5+^ unit within the compound.^[^ ^33^ ^]^ But what of the Mo_2_Cr compound **2**? It is oxidized at only 30 mV above the parent compound **1**. It's most likely that this redox event corresponds to the oxidation of Cr^2+^ to Cr^3+^. Support for this conclusion comes from reports of the oxidation of Cr_3_(dpa)_4_X_2_ compounds, which yield unsymmetric species in which a Cr^3+^ ion is appended to an intact Cr≡Cr quadruply bonded group.^[^ ^43^, ^44^ ^]^

In addition to the Mo_2_^4+/5+^ signals described above, **3**–**5** display a second, smaller peak that has an E_1/2_ matching that of **1**. We suspect that these signals indicate an equilibrium in solution between the trimetallic HEMAC complex and the bimetallic metalloligand precursor, Equation 1. The peak corresponding to **1** does not grow with successive scans, further supporting that the extra peak is due to an equilibrium between two species and is not due to degradation of the HEMAC upon oxidation. The peak magnitude is also not dependent on the scan rate of the measurement down to 50 mV/s, indicating that the equilibrium is slow relative to the scan rate.

Mo_2_M′(dedpa)_4_Cl_2_ ⇌Mo_2_(dedpa)_4_ + M′Cl_2_ Equation 1: M′ = Mn, Fe, Co.

**Figure 10:**
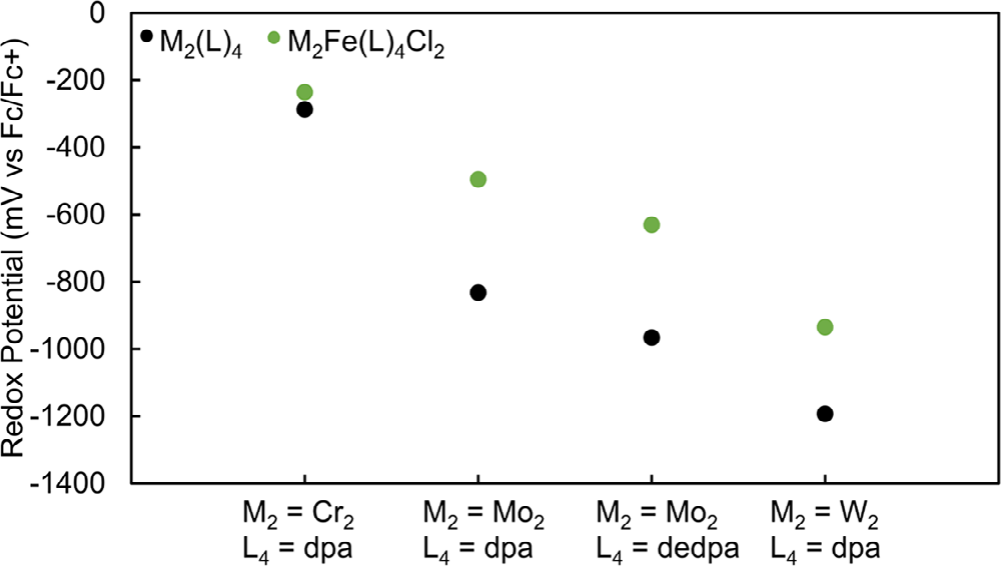
A comparison of redox potentials of metalloligand M_2_(L)_4_ species compared to their Fe‐containing HEMAC complexes. The potentials of the HEMACs are universally higher than the metalloligands, with the difference being largest in Mo_2_ containing HEMACs, indicating that the Mo_2_‐Fe interaction is the most covalent of the three.

Although the Mo_2_^4+/5+^ redox couples for **3**–**6** lie within a narrow range, a clear trend emerges wherein the potential becomes more positive with increasing electronegativity of the heterometal (Table 5). Electronic structure studies on the Mo_2_M’(dpa)_4_(OTf)_2_ series indicated that the heterometallic bonds become more covalent across the series from Mn to Ni.^[^ ^27^ ^]^ Thus, we can conclude that the increasing covalency of the Mo_2_‐M′ bond is modifying the oxidation potential of the Mo_2_ core to make it more difficult to oxidize. From previously reported data on similar compounds with a Cr_2_, Mo_2_, and W_2_ core (Table 5), we can draw more conclusions about the redox properties of **1–6**. We note that redox potentials for all [M_2_(L)_4_]^0/+^ metalloligands are known as well as for [M_2_Fe(L)_4_Cl_2_]^0/+^ compounds. These potentials are depicted in Figure 10, where we see that the Fe complexes lie uniformly higher in potential than the metalloligands. In the Cr_2_Fe case, oxidation is attributed to the Fe^2+/3+^ couple,^[^ ^37^ ^]^ but for the other compounds, the M_2_Fe oxidation is centered at the multiply bonded M_2_^4+/5+^ core. Addition of Fe to Mo_2_ species **1** or **7** causes large increases in the Mo_2_^4+/5+^ couple, ∼340 mV, compared to ∼260 mV for the W_2_ case. It's therefore likely that heterometallic covalency is maximal for the Mo_2_─Fe bonds. Another important takeaway from Figure 9 is that the impact of the electron donating ethyl substituents of the depa ligand is clearly seen in the redox potentials of both **1** and **4**: both complexes show a ∼130 mV negative shift of their Mo_2_^4+/5+^ couples.

**Table 5:** Oxidation potentials for some HEMAC compounds taken in DCM and referenced against Fc/Fc^+^

Compound	M_2_^4+/5+^ E_1/2_ [mV]
**1**	−966
**2**	−969
**3**	−635
**4**	−630
**5**	−610
**6**	−556
Cr_2_dpa_4_^[^ ^37^ ^]^	−287^[^ ^a^ ^]^
Cr_2_Fedpa_4_Cl_2_^[^ ^37^ ^]^	−236
Mo_2_dpa_4_BPh_4_^[^ ^35^ ^]^	−832
Mo_2_Crdpa_4_Cl_2_^[^ ^32^ ^]^	−860
Mo_2_Fedpa_4_Cl_2_^[^ ^40^ ^]^	−495
Mo_2_Nidpa_4_Cl_2_^[^ ^33^ ^]^	−418
W_2_dpa_4_BPh_4_^[^ ^35^ ^]^	−1193
W_2_Crdpa_4_Cl_2_^[^ ^32^ ^]^	−1010
W_2_Fedpa_4_Cl_2_^[^ ^40^ ^]^	−935

^[a]^ this feature is irreversible.

### 2.6 Electronic Structure Analysis

**Figure 11:**
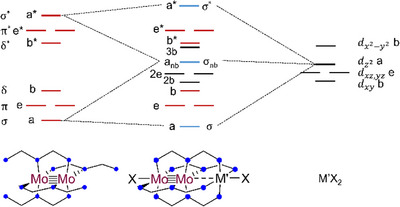
Idealized molecular orbital diagram for **1**, left, and its interaction with a tetragonally symmetric M′X_2_ unit to produce an Mo_2_M′(dedpa)_4_X_2_ compound (center).

Density functional theory (DFT) calculations were used to analyze the structures and bonding of **2–6** further. We utilized the BP86 functional for these calculations, which has been found in our previous research efforts to be a good choice for the Mo_2_M′(dpa)_4_X_2_ compounds.^[^ ^27^ ^]^ Furthermore, bond lengths from the optimized structures are generally in good agreement with the crystallographic models, with the exception of the Mo‐M′ bond lengths, which have errors as high as ± 0.15 Å (Table 2 and Table S3). The Mo‐M′ bond lengths were not improved with the use of the B3LYP or PBE functionals, so BP86 was used for models of **2–6**. As seen for the series of Mo_2_M′(dpa)_4_(OTf)_2_ compounds, the Mo─M′ Mayer bond orders increase from **2** to **6**. Bonding is understood in reference to the molecular orbital model in Figure 11.^[^ ^27^ ^]^ In this diagram, Mo_2_‐centered electrons occupy red orbitals and M′‐centered electrons occupy black orbitals. The blue orbitals, of σ symmetry, are mixed such that the three metal $d_{z^{2}}$ orbitals form three‐center bonding, nonbonding, and antibonding orbital combinations. Since all M′^2+^ ions are high‐spin, compounds **2**–**6** have electron configurations of σ^2^σ_nb_^1^, with a 3‐center, 3‐electron σ bond. The change in Mo─M′ Mayer bond order stems from the change in M′ effective nuclear charge (Z*) from **2** to **6**, with the Mo─M′ bonds becoming more covalent from Cr to Ni.

Electronic spectra for **1**–**6** are best understood with reference to the molecular orbital model in Figure 11. The Mo_2_ unit has its valence *d* orbitals split into bonding and antibonding orbitals of σ, π, and δ symmetry with a relatively large energy gap between the highest‐occupied δ and lowest unoccupied δ^*^. The valence 3*d* orbitals of the first‐row metal atom generally fall between this gap, though the energies are uniformly lowered from Cr to Ni with increasing Z*. For M′ = Cr, the σ_nb_ orbital lies energetically between the Mo_2_ δ and δ^*^ orbitals as shown in Figure 11, but the increase in Z* from Cr to Ni causes the α M′ orbitals for the later M′ metals to fall below the Mo_2_ δ orbital.

**Figure 12:**
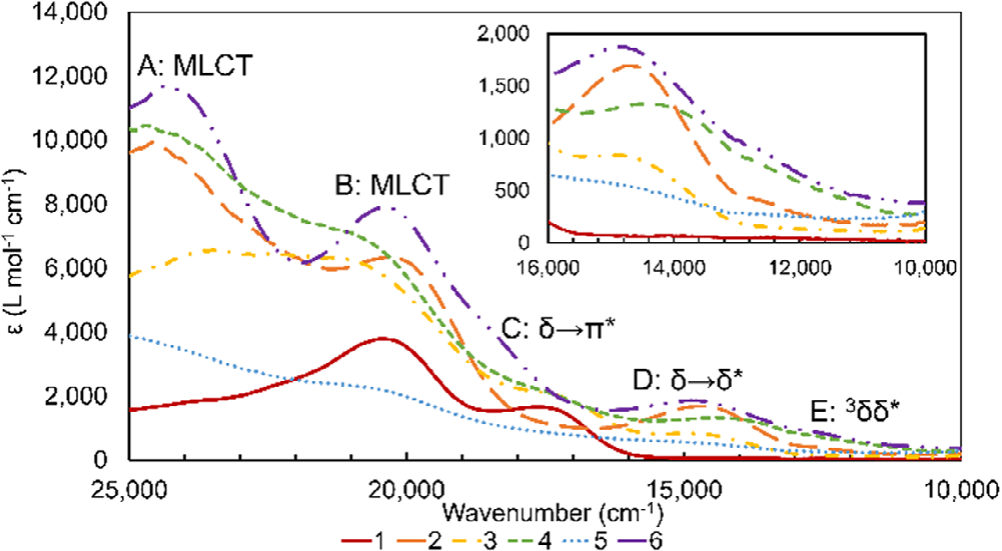
UV‐vis spectra for **1**–**6** with an inset of the 10000–14000 cm^−1^ region. There are five distinct transitions that are present in **2**–**6**: two metal to ligand charge transfer bands (∼24000 and 20500 cm^−1^), a transition assigned as δ to π* (17000–18500 cm^−1^), a δ to δ* transition (∼14500 cm^−1^), and a low energy transition assigned as a transition involving the Mo_2_^3^δδ* state (12500 cm^−1^).

Electronic absorption spectra of **1–6** were taken in DCM solution (Figure 12). The spectrum for **1** features two main absorption features at 20408 and 17540 cm^−1^ assigned (with the aid of TD‐DFT) as the Mo_2_ δ→δ^*^ transition (17540 cm^−1^) and a Mo_2_ δ→π* transition (20408 cm^−1^). Compounds **2–6** have absorption features at 24200–24700 cm^−1^ (transition A), 20325–20900 cm^−1^ (transition B), 14000–14800 cm^−1^ (transition D), and ∼12500 cm^−1^ (transition E). An additional peak is visible in **3–6** at 17000 cm^−1^ (**3**–**5**) and 18500 cm^−1^ (**6**) (transition C). Transitions A and B are of an intensity that suggests metal to ligand charge transfer (MLCT), while transitions C‐E are significantly weaker, ∼ 1000 to 2,000 L mol^−1^ cm^−1^, suggesting that they are ligand field transitions. Based on its position, intensity, similarity to the absorption feature in **1**, and assignments in Mo_2_M’(dpa)_4_Cl_2_ compounds, transition D should be assigned to the Mo_2_ δ‐δ^*^ transition for each compound. Transitions C and E, however, are not assigned in the analogous Mo_2_M'(dpa)_4_Cl_2_ complexes or in the Mo_2_M'(dpa)_4_OTf_2_ complexes.^[^ ^27^, ^45^ ^]^ We therefore have employed TD‐DFT methods at the cam‐B3LYP level to explore the electronic transitions further on models of **2**–**6** whose geometries were optimized as described in the DFT section above. The experimental and calculated absorption features are collected together in Table 6.

**Table 6:** Electronic absorption transition peaks (cm^−1^) and molar absorptivity values, ε (L mol^−1^ cm^−1^)

Transition	1	2	3	4	5	6	Assignment
A. (ε)	−	24631 (9930)	23641 (6530)	24510 (10300)	24752 (3790)	24272 (11700)	MLCT
B. (ε)	−	20325 (6150)	20877 (6180)	20833 (6970)	20534 (2230)	20325 (7890)	MLCT
C. (ε)	20408 (3790)	−	17007 (1790)	17182 (1880)	−	18450 (4030)	δ → π*
D. (ε)	17544 (1610)	14641 (1840)	14641 (823)	14025 (1290)	14728 (540)	14771 (1870)	δ → δ*
E. (ε)	−	12500 (393)	12500 (275)	12500 (718)	12500 (274)	12500 (849)	3δδ*

These results confirm the MLCT character of bands A and B, as well as that D results from a Mo_2_‐based δ‐δ* transition. Interestingly, transition C is assigned to a δ‐π* transition on the Mo_2_ unit that has not been independently visible in other Mo_2_M'(dpa)_4_X_2_ complexes.

**Figure 13:**
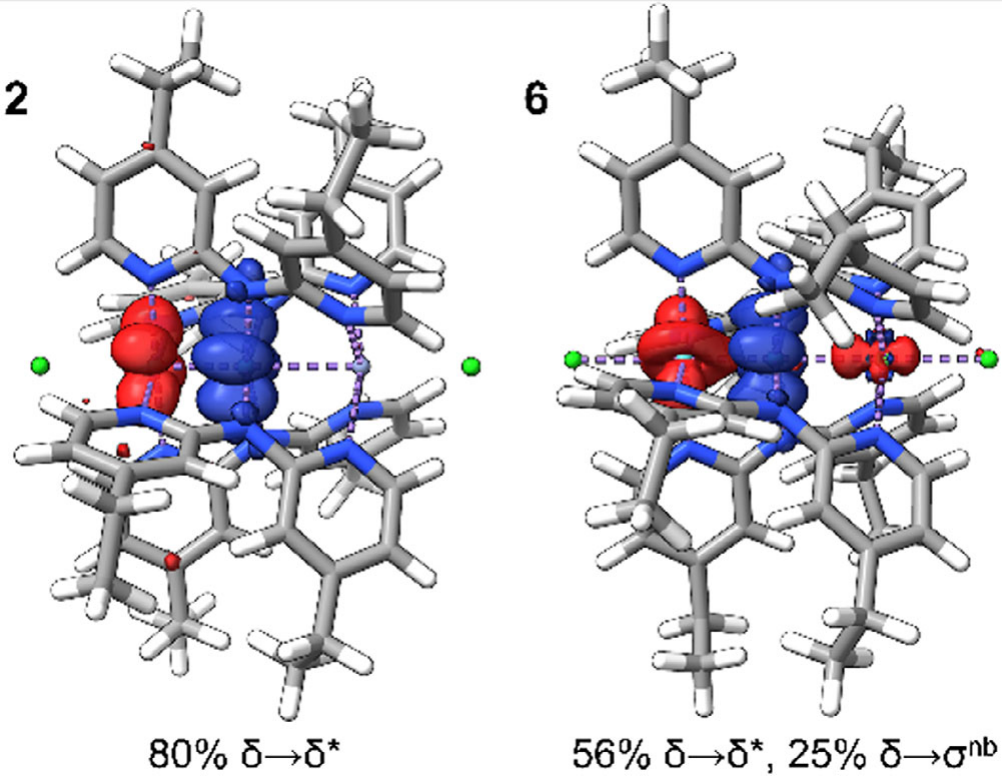
Electron density difference maps (EDDMs) for transition D in **2** (left) and **6** (right) where the electron density is being transferred from a δ symmetry orbital (blue) to a δ* or combined δ* and charge transfer orbital (red). The charge transfer character increases going from **2** to **6**.

Upon inspection, the δ‐δ* transitions of **2–6** do not have the same energies, suggesting a perturbation of the Mo_2_ δ‐bond by the neighboring M′ atom. The breakdown of TD‐DFT orbital contributions for this transition indicates that the transition has Mo_2_→M′ charge transfer character in addition to δ‐δ* character and that the degree of charge transfer character increases as the Mo─M′ bonds become more covalent from M′ = Cr to Ni (Figure 13). Transition D has an overwhelming (∼ 80%) δ‐δ* character for **2**, but the δ‐δ* character decreases to only 56% in **6**, in which the transition has an additional δ‐σ_nb_ character (25%). Thus, as the electronegativity of M′ increases and the Mo─M′ bonds become more covalent, the sigma MMCT character of band D increases. The same trends can be seen in transition C, though the trends in orbital contribution are less clear.

Compounds **2**–**6** also display low‐intensity bands at around 12500 cm^−1^ (Band E) that may involve a formally spin‐forbidden excited state of the Mo_2_ unit. Because the position of band E is independent of M′, it is tempting to assign this to a Mo_2_‐centered transition. Specifically, a triplet Mo_2_ δ‐δ* excited state may be antiferromagnetically coupled to the high‐spin M′^2+^ ion such that this excited state becomes accessible. For diamagnetic quadruply‐bonded Mo_2_ compounds, the transition from the singlet δ^2^δ*^0^ to the triplet δ^1^δ*1 state (a.k.a., the 3δ‐δ* transition) has eluded detection, being a rigorously spin‐forbidden transition. This energy splitting has been determined experimentally for some phosphine‐supported Mo_2_ complexes to be in the range of 1300 to 3000 cm^−1^, being strongly influenced by the P─Mo─Mo─P torsion angle around the quadruple bond.^[^ ^46^ ^]^ Further estimates of the ^3^δ‐δ* energy in the range of 2400 to 10400 cm^−1^ have been made for a range of quadruply bonded compounds and their cations with halide, carboxylate, and phosphine supporting ligands.^[^ ^5^ ^]^ The observed energy here, 12500 cm^−1^, is notably larger than in these previous reports, but we anticipate that this is due to the fact that the dedpa ligand is a strong π‐donor, raising the energy of the δ* orbitals and leading to a large one‐electron δ‐δ* splitting (Δ*W*). The observed spin‐allowed (“singlet”) δ‐δ* transitions for **2**–**6** lie in an expected energy range for Mo_2_ quadruple bonds because the energies of these transitions are more influenced by two‐electron interactions (quantum mechanical exchange, *K*), whereas the spin‐forbidden (“triplet”) δ‐δ* energy is affected more by Δ*W*.^[^ ^5^, ^47^ ^]^ Presumably, these ^3^δ‐δ* transitions also occur in the corresponding Mo_2_M’(dpa)_4_X_2_ compounds, but would be lower in energy than in **2**–**6** because the dpa ligand is less π‐donating than the dedpa ligand.

The assignment of Band E to a ^3^δ‐δ* state is supported by spin unrestricted TD‐DFT calculations on **6**, which predict a pair of transitions at 12024 and 12196 cm^−1^ with nonzero intensity that are mostly Mo_2_ δ‐δ* with some δ‐π* character. It is notable that the addition of a paramagnetic metal to the diamagnetic Mo_2_ unit provides a mechanism for increasing the intensity of the spin‐forbidden Mo_2_ transitions.

With both singlet δ‐δ* energies (average: 14561 cm^−1^) and triplet δ‐δ* energies (12500 cm^−1^) available, we may estimate both *K* and Δ*W* (ignoring the effects of configuration interaction). We find an average value of *K* = 2061 cm^−1^, which is smaller than the range of values determined for chloro, phosphino, and carboxylato complexes (4900─8400 cm^−1^).^[^ ^5^ ^]^ The smaller value determined here is consistent with a higher degree of Mo─N covalency than is seen with the other types of ligands. The one‐electron δ‐δ* splitting Δ*W*, estimated here to be ∼12300 cm^−1^ on average, is within the anticipated range for quadruply bonded complexes. Due to the stronger electron donation of the dedpa ligand, Δ*W* is larger than the lowest energy observed electronic transition for the [Mo_2_(dpa)_4_]^+^ cation (∼11000 cm^−1^),^[^ ^35^ ^]^ which is a direct measure of Δ*W*.

### 2.7 DFT Reaction Mechanism Studies

To gain insight into the fluxional behavior of **1** as compared to Mo_2_(dpa)_4_ (**7**), DFT studies were conducted on both compounds. Crystal structures of both **1** and **7** adopt the *trans*‐(2,2) configuration of ligands (Figure S32), though there is evidence from NMR spectroscopy that other conformations of **1** exist in solution. Importantly, rearrangement of the equatorial ligands is required in order to form the trimetallic HEMACs. The metalloligand precursor compounds must undergo a rearrangement to the (4,0) configuration in which there is a “pocket” to allow for the binding of a third metal atom (Scheme 1).

**Scheme 4:**
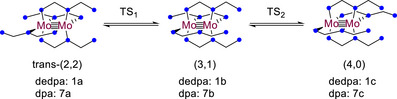
Optimized structures for the species involved in ligand shuffling.

Geometries of both **1** and **7** were optimized to produce **1a** and **7a**. These optimized geometries were found to be in good agreement with experimentally observed bond distances. The *trans*‐(2,2) structures **1a** and **7a** were used as a starting point to optimize new structures in the (3,1) configuration, **1b**, **7b**, and in the (4,0) configuration, **1c**, and **7c**. Using these optimized structures, the nudged elastic band (NEB) method^[^ ^48^, ^49^ ^]^ was employed to locate the transition states along the stepwise pathway shown in Scheme 4 going from the *trans*‐(2,2) to (3,1) geometry (**1TS1, 7TS1**), and from the (3,1) to (4,0) geometry (**1TS2, 7TS2**). Structural data and thermodynamic info for these intermediate species are given in Table 7.

**Table 7:** Energy decomposition breakdown for 1 and 7 given in kcal mol^−1^

	*E*_Total_	*E*_M‐M_	*E*_Lig._	*E*_Int._		*E*_Total_	*E*_M‐M_	*E*_Lig._	*E*_Int._
1a	0	0	0	0	7a	0	0	0	0
1TS1	17.21	−0.07	11.18	6.1	7TS1	18.84	−0.81	6.16	13.50
1b	0.19	0.25	12.79	−12.85	7b	4.12	−0.85	3.24	1.74
1TS2	19.67	0.14	20.02	−0.49	7TS2	22.53	0.03	11.96	10.55
1c	5.17	0.69	17.6	−13.12	7c	7.90	0.21	9.59	−1.90

**Scheme 5:**
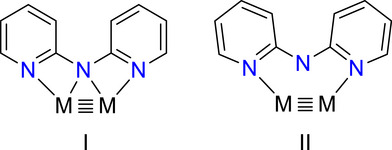
Proposed coordination modes along the ligand shuffling pathway. Coordination mode **I** has all three N atoms interacting with the metals of the core, whereas coordination mode **II** has only the N atoms in the pyridine rings bound to the core while the amido N is essentially unbound.

The concept of ligand “shuffling” to achieve a (4,0) conformation from a *trans*‐(2,2) starting point was proposed as early as 2001 for pyridylamine and pyridylformamidine complexes based on the observation of several isomers in solution by ^1^H NMR spectroscopy.^[^ ^50^, ^51^ ^]^ We studied fluxionality of Cr_2_(dpa)_4_ by ^1^H NMR spectroscopy in the presence and absence of ZnCl_2_ and proposed the symmetric coordination mode **I** seen in Scheme 5 as accounting for intermediate or transition state geometries.^[^ ^40^ ^]^ The suggestion of coordination mode **I** was based on the fact that this coordination mode has been crystallographically established in several other coordination compounds.^[^ ^35^, ^52^, ^53^, ^54^, ^55^, ^56^ ^]^

The NEB calculations reported here provide us with the first structural picture of this ligand shuffling process. Upon moving from the *trans*‐(2,2) to the (3,1) conformation, and also from the (3,1) to the (4,0) conformation, the potential energy landscape consists of a single transition state, **TS1** or **TS2**, respectively, and no metastable intermediate species. Interestingly, the ligand undergoing the longitudinal shuffle does not adopt coordination mode **I** along its pathway, rather we optimize to a new coordination mode, **II**, which sees each pyridine ring bonded to a different metal but the amido group essentially uncoordinated. To our knowledge, there are no existing precedents for this coordination mode of the dpa ligand.

**Figure 14:**
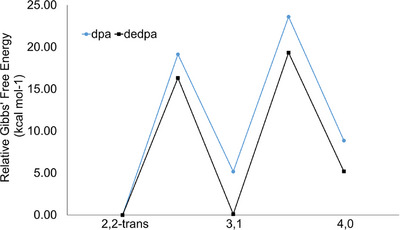
Relative Gibbs free energies for the ligand shuffling of **1** and **7** for a stepwise ligand shuffling mechanism. The energies for all transition states and intermediates are relative to the (2,2)‐trans configuration of both metalloligands. For each intermediate and transition state after the (2,2)‐trans starting point, the energies are higher for **7** than **1**, but by only ∼5 kcal mol^−1^ for each point. Calculations were performed with the BP86 functional.

The potential energy landscape for equatorial ligand shuffling in **1** and **7** is shown in Figure 14. Notably, for **1** we find the trans‐(2,2) and the (3,1) isomer to be nearly isoenergetic, whereas the trans‐(2,2) isomer is clearly the lowest energy stationary point for **7**. Additionally, we calculate the ∆*G* ^‡^ at 298 K for both **TS1** and **TS2** to be lower for **1** than it is for **7**. This ∆*G* can further be broken down into its enthalpy and entropy components with values of ∆*H* of 21.9 and 19.0 kcal/mol for **1** and **7,** respectively, and values of ∆*S* of 5.7 and 1.2 cal/mol*T for **1** and **7**. These calculations agree with the fact that there are multiple isomers of **1** that are observable in solution. Although the stronger Mo─N bonds in **1** contribute to a higher transition state enthalpy, the additional degrees of freedom from the ethyl substituents likely make **TS1** for **1** more favorable from an entropy standpoint.

To gain further insight into the cause of the differences in transition state energies, an energy decomposition analysis was performed on **1a,b,c**, **7a,b,c**, and their associated transition states. Here, we include further calculations to determine the energies of just the Mo_2_ core (*E*_Mo_) and of just the four ligands (*E*_L_), frozen in the geometries optimized for the associated compounds. The total energy *E*_Total_ of the system must be the sum of these two energetic terms and an interaction energy, *E*_Int_, indicating the favorability of the metal‐ligand bonds. The term *E*_Int_ is thus determined by Equation 2.

(2) $$E_{Total}=E_{Mo}+E_{L}+E_{Int}$$

The results of this analysis are showcased in Table 7, with all values relative to the structures of either **1a** or **7a**. Uniformly, E_Mo_ values are relatively small, indicating that reorganization of the Mo≡Mo bond does not play a substantial role in the energetics of the reaction. Ligand rearrangement energies and the interaction energies, however, are both large, meaning that they play substantial roles. When comparing the dpa and dedpa ligands, the ligand reorganization energy along the shuffling pathway is higher for dedpa, but the interaction energy is less destabilizing in the transition states and is actually stabilizing for intermediates **1b** and **1c**. These effects lead to lower barriers in electronic energy for both TS1 and TS2, and greater overall favorability of the shuffling reaction for **1** versus **7**. We attribute this energetic benefit to greater electron donation into the Mo_2_ core (or stronger Mo‐ligand bonding) from the four relatively electron‐rich dedpa ligands.

These two conflicting effects of higher reorganization energy due to a larger ligand and more stabilized transition states due to electrondonating groups suggest that other substituted dpa ligands are likely to show behavior that cannot be boiled down to a simple, single Hammett substituent effect.

## 3 Conclusion

In this work, we have presented the novel syntheses of Mo_2_(dedpa)_4_ (**1**) and five new trimetallic compounds of the form Mo_2_M′(dedpa)_4_Cl_2_ with M′ = Cr, Mn, Fe, Co, and Ni (**2**–**6**). We synthesize these compounds using a novel solution‐state synthetic method that we show also works for dpa HEMACs of the same form. We find that the effects of the electron donating ethyl substituents in the dedpa ligand are not felt uniformly by the Mo_2_ and M′ groups. Contrary to our expectations, the more electron‐rich dedpa ligand provides a *weaker* ligand field at the M′ site with a concomitantly stronger field at the Mo_2_ core, which also allows for lowenergy triplet δ‐δ* states to be visible in the absorption spectra of **2**–**6**. Cyclic voltammetry confirms that the dedpa ligand creates a more electron‐rich, and thus easier to oxidize Mo_2_ unit with the series of compounds as compared to dpa compounds with similar structures. Computational analysis of the isomerism of **1** in comparison to an unsubstituted version showed that, while reorganization energy is larger for the more bulky dedpa ligand, the stronger metal‐ligand bonding leads to lower energy transition states along the ligand shuffling reaction pathway. Overall, these results suggest that the influence of substituents on the dpa ligand is not distributed evenly between the metals of **2**–**6**, with a stronger effect present on the Mo atoms, and a weakening of the ligand field at the M′ heterometal. The fact that these effects occur counter to expectation based on metal atom electronegativities highlights the importance of the heterometallic bond polarity umpolung in these compounds.

## Supporting Information

The .xyz files for the geometry optimized structures referenced in the paper are housed in the Dryad data repository. Deposition Numbers 2 456 099 (**1**), 2 455 982 (**2**), 2 456 098 (**3**), 2 455 984 (**4**), 2 455 983 (**5**), and 2 456 097 (**6**) contain the supplementary crystallographic data for this paper. These data are provided free of charge by the joint Cambridge Crystallographic Data Centre and Fachinformationszentrum Karlsruhe Access Structures service. The authors have cited additional references within the Supporting Information.^[^ ^30^, ^31^, ^36^, ^57^, ^58^, ^59^, ^60^, ^61^, ^62^, ^63^, ^64^, ^65^, ^66^, ^67^, ^68^, ^69^, ^70^, ^71^, ^72^, ^73^, ^74^, ^75^, ^76^, ^77^, ^78^, ^79^, ^80^, ^81^, ^82^, ^83^, ^84^, ^85^, ^86^, ^87^, ^88^, ^89^, ^90^, ^91^, ^92^, ^93^, ^94^, ^95^ ^]^

## Conflict of Interest

The authors declare no conflict of interest.

## Supporting information



Supporting Information

Supporting Information

## Data Availability

The data that support the findings of this study are available in the supplementary material of this article.
