# Supplementary material for: Asymmetric Ligand Field Effects in Electron‐Rich Heterometallic Extended Metal Atom Chain Compounds
Source: Chemistry. 2025 Oct 22;31(65):e02090. doi: 10.1002/chem.202502090 (PMC12642394; doi:10.1002/chem.202502090)
Supplement: Supplementary file 1 — Supporting Information [file CHEM-31-e02090-s001.pdf]

Supporting Information for

Asymmetric Ligand Field Effects in Electron-Rich Heterometallic Extended Metal Atom Chain Compounds

Rebecca K. Walde, Trey C. Pankratz, Amelia M. Wheaton, Milton Acosta, and John F. Berry*

Department of Chemistry, University of Wisconsin – Madison, 1101 University Ave, Madison, WI 53706
Email: berry@chem.wisc.edu

Table of Contents:

1. General Methods 2
2. Synthetic Methods 4
3. Collected IR Spectra 7
4. Collected NMR Spectra 13
5. Collected Cyclic Voltammograms 33
6. Table of EPR Transitions in **3** 39
7. Collected Magnetometry Data 40
8. Crystallographic Figures 45
9. Computational Figures 56
10. References 62

Materials and General Methods

All reactions were carried out under a dry N_2 atmosphere using Schlenk techniques and glovebox methods. Anhydrous hexanes, diethyl ether, 1,4-dioxane, and tetrahydrofuran were purchased from Sigma-Aldrich. Acetonitrile and dichloromethane were freshly distilled from CaH_2 under an N_2 atmosphere. All solvents were stored over 3 Å molecular sieves under an N_2 atmosphere for at least 3 days before use. Naphthalene was purchased from Sigma-Aldrich and sublimed prior to use. The metal(II) chloride salts (M = Cr, Fe, Ni) were purchased from Strem Chemicals and used as received. The Co(II) chloride salt was purchased from Strem Chemicals and dried in thionyl chloride before use. The Mn(II) chloride THF adduct was prepared according to literature.[1] Eicosane was purchased from Sigma-Aldrich, melted and fused four times under vacuum, and stored under an atmosphere of N_2. Tris-(dibenzylideneacetone)-dipalladium(0), and 1,3-bis(diphenylphosphino)propane were purchased from Strem and Oakwood chemical respectively and were used as received. 2-amino-4-ethyl pyridine and 2-bromo-4-ethyl pyridine were prepared according to the literature.[2-4] IR spectra were taken on a Bruker ALPHA II FTIR spectrometer using an attenuated total reflectance (ATR) adapter. Elemental analyses were carried out by Midwest Microlabs, LLC in Indianapolis, IN. ^{1}H NMR spectra were obtained on a 400 MHz Bruker Advance III spectrometer and 500 MHz Bruker Avance NEO. MALDI mass spectra were obtained on Bruker Impact™ II MALDI mass spectrometer. Electronic absorption spectra were taken on a StellarNet Inc BLACK-Comet UV-VIS spectrometer using a dip probe with a 2 mm tip.

Physical Measurements: EPR data for **3** and **5** at 5 and 10 K, respectively, were acquired on a Bruker ELEXSYS E500 EPR spectrometer equipped with a Varian E102 microwave bridge. An Oxford Instruments ESR-900 continuous-flow helium cryostat and an Oxford Instruments 503S temperature controller were used to control the sample temperature. The measurement conditions for **3** were as follows: 9.38 GHz, 4 G modulation amplitude, 4000 G center field, 8000 G sweep width, 0.6325 mW microwave power, 65 dB gain, 10.24 ms time constant and 20.48 ms conversion time. The measurement conditions for **5** were as follows: 9.38 GHz, 2 G modulation amplitude, 3000 G center field, 6000 G sweep width, 0.6325 mW microwave power, 60 dB gain, 10.24 ms time constant and 20.48 ms conversion time. All EPR data were simulated using the EasySpin software package.[5]
Variable temperature magnetic susceptibility measurements of polycrystalline samples of **2-6** suspended in eicosane in polycarbonate sample holders were performed on a SQUID magnetometer (MPMS 3 Quantum Design) in the temperature range 1.8–300 K with an applied field of 0.1 Tesla. Variable temperature/variable field measurements of the same samples of **2-6** were also collected on the same magnetometer at 2, 4, 6, and 8 K with the field swept from 0.1 to 7 T. Experimental susceptibility data were corrected for the underlying diamagnetism using Pascal's constants.[6] The data for **2-6** were modeled using the program PHI.[7] Data for **2-6** were parameterized using the following general formalism of the spin Hamiltonian:

$$\hat{H} = \beta \vec{S}\boldsymbol{g}\vec{H} + \vec{S}\boldsymbol{D}\vec{S}$$

where β is the Bohr magneton, $\vec{H}$ is the applied external magnetic field, and $\vec{S}$, $\boldsymbol{g}$, and $\boldsymbol{D}$ are the spin vector, electronic **g**-tensor, and zero-field splitting (ZFS) tensors, respectively. Including a rhombicity term did not improve the fits and therefore the E term was not used in models of the magnetic data for **2-6**. Simultaneous fits of the susceptibility and reduced magnetization data allowed for assignment of the sign of $D < 0$ for both **2** and **4** and $D > 0$ for **5**, and **6**. Independent fits of the susceptibility and reduced magnetization data of **3** allowed for the assignment of the sign of D > 0. Temperature independent paramagnetism (TIP) contributions to the experimental susceptibility were included in the susceptibility models for **2-6**.

Mössbauer spectroscopy measurements were performed with a 1024 channel See Co model W304 resonant gamma-ray spectrometer using ^{57}Co on Rh foil as a gamma-ray source (initial strength = 25 mCi, obtained from Ritverc Isotope Products). The source velocity range used was ±6 mm s^{-1} and measurements were conducted under vacuum. A Lakeshore model 336 temperature controller and a Janis model SHI-850 cryostat were used to reach cryogenic temperatures. Data were fitted with the WMOSS4F software package,[8] using an adaptive nonlinear least-squares algorithm.[9] Data were collected at 77 K for **4**. Refined parameters were the isomer shift (δ), quadrupole splitting (ΔE_Q), and a single linewidth (Γ) was modeled by the full-width at half-maximum (FWHM). Isomer shifts are referenced

to α-Fe foil at 77 K. The quality of the chemical models used to fit **4** were assessed using the reduced χ^2 of the overall fits. Here, the reduced χ^2 is defined as the χ^2/d, where d is the degrees of freedom (the number of data points – the number of fit parameters). The χ^2 statistic is given by: $\chi^2 = \sum_1^n(\frac{\chi_{observed}-\chi_{expected}}{\sigma_x})^2$ where n is the number of data points, $\chi_{observed}$ is the observed data, $\chi_{expected}$ is the expected data, and σ_x is the standard deviation of the data.

Electrochemical experiments were performed in a MBraun Unilab glovebox with a nitrogen atmosphere using a BioLogic SP-200 potentiostat in dry, degassed CH_2Cl_2 containing 0.1 M NBu_4PF_6 (tetrabutylammonium hexafluorophosphate, Aldrich, 98%, recrystallized) as the supporting electrolyte. A glassy carbon disk (3 mm diameter, CH Instruments) and a platinum wire were respectively used as the working electrode and the counter electrode in cyclic voltammetry experiments. The glassy carbon working electrode was polished between experiments. All working electrode potentials were measured versus a $Ag/AgNO_3$ reference electrode (Pine Research) containing an internal solution of 100 mM Bu_4NPF_6 and 10 mM $AgNO_3$ in CH_3CN. The Ag electrode was lightly polished with 600 grit sandpaper between trials to remove any surface corrosion. Solid ferrocene was dissolved in analyte solution at the end of each cyclic voltammetry experiment to provide an internal reference for calibrating potentials to the $Fc^{0/+}$ redox couple.

Computational Methods: Calculations were performed using the ORCA program,[10] version 5.0.0 using unrestricted Kohn-Sham (UKS) DFT.[11-13] The ZORA keyword was employed to account for relativistic effects.[14] The D3 dispersion correction, as well as the resolution of identity and chain-of-spheres approximations were employed (when appropriate, given functional choice), using the D3 and RIJCOSX keywords respectively.[15-16] The geometry optimizations and frequency calculations on **2-6, 1_{TS1}**, **1_{TS2}**, **7_{TS1}**, and **7_{TS2}** were preformed using the BP86 functional,[17-18] with the transition states being located using the nudged elastic band method. The geometry optimizations and frequency calculations on **1a-c** and **7a-c** were done using BP86,[17-18] PBE,[19] and B3LYP[20-23] functionals to determine best functional choice. All geometry optimizations and frequency calculations were performed with the ZORA-def2-SVP basis set on all atoms, other than the Mo atoms where the ZORA-def2-TZVP basis was employed.[17-18] One negative frequency was found for the structure of **3**, but the frequency corresponded to movement of one of the ethyl groups, and the frequency was sufficiently small (–3 cm^{-1}) as to be disregarded. The Mössbauer calculations utilized the CP(PPP) basis on Fe and used the previously reported regressions to determine isomer shift.[24-26] TD-DFT single-point calculations on **2-6** employed the CAM-B3LYP functional[27] and located the first 50 roots. The TD-DFT single-points and the calculations on **1** and **7** employed the conductor-like polarizable continuum model (CPCM) to account for solvation effect,[11] with DCM and THF respectively. Löwdin population analysis was used to determine spin densities and orbital contributions.[28-29] Visualizations were carried out with the UCSF ChimeraX package.[30-32] Symmetry labels presented in the molecular orbital diagram were assigned by inspection to conform with the idealized C_4 point group.

Crystallographic Refinement Details for 1-6: Crystals for **1-6** were selected under oil and attached to the tip of a MiTeGen MicroMount. Each crystal was mounted under a stream of cold nitrogen at 100(1) K and centered in the X-ray beam using a video camera. The crystal evaluation and data collection for **1-3** and **6** were performed on a Bruker D8 VENTURE PhotonIII four-circle diffractometer with Cu K_α (λ = 1.54178 Å) radiation and a detector to crystal distance of 4.0 (**2**) or 4.5 (**1**, **3**, **6**) cm. Crystal evaluation and data collection for **4** and **5** were performed on a Bruker Quazar SMART APEXII diffractometer with Mo K_α (λ = 0.71073 Å) radiation at a detector distance of 4.96 cm. Data were collected using a routine to survey the entire sphere of reciprocal space and were indexed by the SMART program.[33-35] The structures were solved using intrinsic phasing and refined by least squares on F^2 followed by difference Fourier synthesis.[36-37] All hydrogen atoms were included in the final structure factor calculation at idealized positions and were allowed to ride on the neighboring atoms with relative isotropic displacement coefficients.

The models for crystals of **2** and **4-6** share very similar features. All four compounds crystallize without solvent in the lattice, but with significant disorder, as described in the main text for **4**. The

compounds can crystallize in one of two metal atom orientations and as a pair of helical enantiomers. The Λ isomer is the dominant isomer in each structure, with an approximate 80:20 ratio of Λ:Δ (specific ratios are given in the figures for each structure). For **2**, **4**, and **5**, the minor component could not be modelled anisotropically. The compounds can also crystallize with the metal atoms facing in one of two directions, in approximately a 60:40 split of the metal atom positions (details for each compound are given below). Compound **1** has a single ethyl group (C14 and C14A) split due to positional disorder. Finally, **3** crystallizes in a different space group and crystal system than the other compounds, leading to a different morphology altogether. Finally, **2**, **4**, and **5** all crystallize as inversion twins, with between 38-49 % contributions from the minor component. This twinning is notably absent in **6**.

The model for **3** displays no apparent disorder in the ligand manifold, but instead crystallizes with both enantiomers appearing as two disconnected 1/8th molecules in the asymmetric unit (see Figure S30 and S31 below). Due to the high symmetry of the system, the metal atoms of **3** require a 50:50 split of the outer Mo atom (Mo2) and the Mn atom (Mn1). The Cl atom (Cl1/Cl1A and Cl2/Cl2A) is also split along the metal atom axis to account for the difference in bond lengths between the Mn-Cl and Mo-Cl bonds. The model for **3** has regions of electron density that seem to correspond to diffuse solvent in the lattice. These solvent molecules could not be modelled, so the SQUEEZE function was employed.[38] The solvent voids appear to approximately correspond to two molecules of CH_2Cl_2 in the asymmetric unit.

Synthetic Methods

2,2′-diethyldipyridyl amine (Hdedpa). the ligand was synthesized using a modification of a literature preparation.[39-40] The precatalyst $Pd_2(dba)_3$ (1.28 g, 1.4 mmol) was combined in a Schlenk flask with dppp (1.29 g, 2.9 mmol). The solids were dissolved in dry, degassed toluene (~30mL). 2-bromo-4-ethyl pyridine (13.00 g, 69.9 mmol) was added by syringe. The resulting solution was allowed to stir for circa 5 minutes. Solid 2-amino-4-ethyl pyridine (9.41 g, 77.1 mmol) and KO*t*-Bu (11 g, 98.0 mmol) were then added. The mixture was then heated to 110°C under N_2 and allowed to reflux for 16 hours. The mixture was cooled to RT and then filtered through Celite; a brown filtrate was collected. Volatiles (including remaining 2-bromo-4-ethyl pyridine) were removed in vacuo to produce a brown oily substance that solidifies upon standing. The crude product was dissolved in ~200 mL of a 10% Hexanes: 90% Ethyl acetate solvent mixture and then filtered through a plug of silica. The filtrate was reduced to dryness, then pure product was obtained by sublimation of the residue at 160 °C under dynamic vacuum Yield: 60% (9.5 g). A ^{1}H NMR spectrum of the pure product in $CDCl_3$ matches literature.

Potassium 2,2′-diethyldipyridyl amine (Kdedpa). Solid Hdedpa (1 g, 4.4 mmol) was added to a flask and dissolved in diethyl ether (~30 mL). In a separate flask, potassium hexamethyl disilazide (KHMDS) (0.97 g, 4.8 mmol) was dissolved in diethyl ether (~20 mL). Both flasks were cooled to –78 °C. With stirring, the KHMDS solution was added over 30 min to the Hdedpa solution to produce a yellow solution with a white precipitate. After warming to RT, the resulting solution was reduced to dryness and the residue was used without further purification. Yield 83% (968 mg). ^{1}H NMR (400 MHz, CD_2Cl_2): δ = 7.49 (s, 2H), 6.92 (s, 2H), 6.17 (s, 2H), 2.41 (q, 4H), 1.14 (t, 6H).

$Mo_2(dedpa)_4$ (1). Solid Kdedpa (2.5 g, 9.4 mmol) was combined with $Mo_2(OAc)_4$ (1.1 g, 2.5 mmol) in ~50 mL THF at RT and allowed to stir overnight, resulting in the formation of a a dark red solution and yellow and white precipitate. The mixture was filtered, and the filtrate was collected and reduced to dryness. The resulting dark red solids were extracted with hexanes and filtered. The filtrate was collected and dried in vacuo. The resulting solids were washed with MeOH and dried to produce a bright red product. SCXRD quality crystals were grown from a concentrated hexanes solution. Yield: 77% (2.1 g). ^{1}H NMR (500 MHz, CD_2Cl_2, –20 °C, ppm): multiple conformations for **1** are observed with the following features, 8.26 (dd), 8.15 (dd), 8.09 (dd), 8.07 (dd), 7.76 (dd), 7.72 (dd), 7.56 (s), 7.45 (s), 7.41 (dd), 7.35 (s), 7.08 (s), 6.96 (s), 6.93 (s), 6.87 (dd), 6.81 (s), 6.75 (dd), 6.72 (dd), 6.68 (dd), 6.66 (dd), 6.47 (dd), 6.00 (dd), 5.85 (dd), 2.64 (q), 2.60 (q), 2.56 (q), 2.50 (q), 2.40 (q), 2.34 (q), 1.26 (t), 1.18 (t), 1.15 (t), 1.12 (t), 1.09 (t), 1.03 (t), 0.89 (t); ^{13}C NMR (500 mHz, CD_2Cl_2, RT): 161.8 (d), 154.2, 149.9, 148.3, 147.7, 146.5, 114.8, 110.9, 110.4, 109.2, 108.3, 28.5, 28.4, 28.2, 14.4, 14.2, 14.1;IR (ATR, cm^{-1}): 2966, 1603, 1552, 1436, 1261, 1174,

1092, 1015, 919, 794; elemental analysis calcd (%) for $Mo_2C_{56}H_{64}N_{12}$: C 61.31 H 5.88 N 15.32; Found: C 61.28 H 5.83 N 15.21.

The trimetallic compounds **2**-**6** were all synthesized using one of two general procedures detailed below. Specifics are noted for each if any changes to the general procedure were made.

General procedure for synthesis in naphthalene (Method 1). Solid naphthalene (5 g) and a stir bar were added to a Schlenk flask and dried overnight under vacuum with stirring. Under an N_2 atmosphere, the flask was charged with **1** (1 eq) and the appropriate metal chloride salt (for **2** and **4-6**) or metal chloride THF adduct (for **3**) (1-2 eq). The flask was brought to 210°C under N_2 for 2-3 hours. The flask was allowed to cool to RT, then the solids were washed with hot hexanes. Remaining solids were extracted into CH_2Cl_2 and filtered. The filtrate was collected and reduced to dryness, then the resulting solids were taken up in minimal CH_2Cl_2 and layered with hexanes to produce crystalline product. Identities of the compounds were confirmed by MALDI mass spectrometry using anthracene as a matrix.

$Mo_2Cr(dedpa)_4Cl_2$ (**2**): 183 mg $Mo_2(dedpa)_4$, 31 mg $CrCl_2$. Yield: 60% (120 mg). MS (MALDI, anthracene) expected *m/z* for $[Mo_2Cr(dedpa)_4Cl_2]^+$: 1220; found 1222

$Mo_2Mn(dedpa)_4Cl_2$ (**3**): 250 mg $Mo_2(dedpa)_4$, 92 mg $MnCl_2{\cdot}2THF$. Yield 46% (128 mg). MS (MALDI, anthracene) expected *m/z* for $[Mo_2Mn(dedpa)_4Cl]^+$: 1188; found 1189

$Mo_2Fe(dedpa)_4Cl_2$ (**4**): 225 mg $Mo_2(dedpa)_4$, 52 mg $FeCl_2$. Yield: 85% (190 mg). MS (MALDI, anthracene) expected *m/z* for $[Mo_2Fe(dedpa)_4Cl]^+$: 1188; found 1187

$Mo_2Co(dedpa)_4Cl_2$ (**5**): 250 mg $Mo_2(dedpa)_4$, 44 mg $CoCl_2$. Yield: 30% (84 mg). MS (MALDI, anthracene) expected *m/z* for $[Mo_2Co(dedpa)_4Cl]^+$: 1191; found 1191

$Mo_2Ni(dedpa)_4Cl_2$ (**6**): 145 mg $Mo_2(dedpa)_4$, 50 mg $NiCl_2$. Yield 56% (72 mg). MS (MALDI, anthracene) expected *m/z* for $[Mo_2Ni(dedpa)_4Cl]^+$: 1191; found 1192

General procedure for synthesis in traditional solvent (Method 2): Solid **1** (1 eq) was combined with a M′(II) chloride salt (M′= Cr, Fe, Co, Ni) or $MnCl_2{\cdot}2THF$ (1-2 eq) and the solids were dissolved in 20-50 mL 1,4-dioxane. The resulting mixture was heated to reflux (140 °C) for 1 day. The mixture was allowed to cool to RT, then the volatiles were removed in vacuo. The resulting solids were washed with diethyl ether until the filtrate ran clear (5-20 mL). The remaining solids were extracted with 20-40 mL DCM and filtered. The filtrate was collected and dried in vacuo to produce the crude product mixture. The crude product was dissolved in minimal DCM, layered with hexanes and allowed to diffuse in a –30 °C freezer to produce crystalline product.

$Mo_2Cr(dedpa)_4Cl_2$ (**2**): 250 mg $Mo_2(dedpa)_4$, 45 mg $CrCl_2$. Yield: 17% (47.7 mg). ^1H NMR (400 MHz, CD_2Cl_2, ppm): 37.6 (s), 33.2 (s), 14.7 (s), 3.69 (s), 2.67-2.55 (m), 1.30 (t); IR (ATR, cm^{-1}): 3064.2, 3033.3, 2965.1, 2930.3, 2872.3, 2505.7, 2408.9, 2161.4, 1978.5, 1941.5, 1605.3, 1525.5, 1474.2, 1397.2, 1312.8, 1259.6, 1221.7, 1179.3, 1052.5, 1015.2, 922.51, 859.41, 813.82, 736.3, 695.95, 656.89; elemental analysis calcd (%) for $Mo_2CrC_{56}H_{64}N_{12}Cl_2{\cdot}1.5(CH_2Cl_2)$: C 51.26 H 4.98 N 12.48; Found: C 51.7 H 5.45 N 11.51

$Mo_2Mn(dedpa)_4Cl_2$ (**3**): 120 mg $Mo_2(dedpa)_4$, 45 mg $MnCl_2{\cdot}2THF$. Yield: 45% (60 mg). ^1H NMR (400 MHz, CD_2Cl_2, ppm): 7.28, 7.21, 6.52, 2.60, 2.38, 2.03, 1.31, 0.93; IR (ATR, cm^{-1}): 3063.9, 3028.9, 2965.3, 2930.2, 2870.5, 2233.8, 2161.9, 2041.3, 2019.1, 1978.5, 1949.2, 1608.1, 1527, 1473.9, 1407.4, 1336.7, 1309.2, 1226.1, 1181.8, 1127.4, 1057.4, 1015.1, 923.31, 860.6, 818.26, 742.52, 696.49, 656.46; elemental analysis calcd (%) for $Mo_2MnC_{56}H_{64}N_{12}Cl_2{\cdot}2(CH_2Cl_2)$: C 50.02 H 4.92 N 12.07; Found: C 49.13 H 5.04 N 11.76

$Mo_2Fe(dedpa)_4Cl_2$ (**4**): 200 mg $Mo_2(dedpa)_4$, 22 mg $FeCl_2$. Yield: 50% (110 mg). IR (ATR, cm^{-1}): 3060.8, 3033.3, 2966.2, 2931.4, 2872.4, 2345.8, 2161.1, 2033.3, 1979.1, 1943.5, 1608.1, 1523.5, 1473.4, 1402, 1336.7, 1309.6, 1221.8, 1181.1, 1129.4, 1055.9, 1015.4, 923.11, 860.02, 814.86, 733.53, 696.41, 655.74; elemental analysis calcd (%) for $Mo_2FeC_{56}H_{64}N_{12}Cl_2 \cdot 2CH_2Cl_2$): C 49.14 H 4.66 N 11.86; Found: C 49.3 H 5.04 N 11.93

$Mo_2Co(dedpa)_4Cl_2$ (**5**): 250 mg $Mo_2(dedpa)_4$, 32 mg $CoCl_2$ (1 eq). After removing dioxane, the solids were washed with THF. After this the general procedure was followed using the resulting green solids. Yield: 65% (180 mg). IR (ATR, cm^{-1}): 3063.9, 3037.5, 2966.2, 2932, 2872, 1604, 1526.9, 1473.6, 1396.5, 1336.2, 1310.3, 1223.1, 1179.8, 1131.1, 1100, 1053, 1015.8, 922.01, 859.55, 814.01, 785.88, 742.82, 696.84, 657.12; elemental analysis calcd (%) for $Mo_2CoC_{56}H_{64}N_{12}Cl_2 \cdot 0.5(CH_2Cl_2)$: C 53.46 H 5.13 N 13.25; Found: C 53.76 H 4.98 N 13.16

$Mo_2Ni(dedpa)_4Cl_2$ (**6**): 220 mg $Mo_2(dedpa)_4$, 40 mg $NiCl_2$. Yield 75% (185.8 mg). ^1H NMR (400 MHz, CD_2Cl_2, ppm): 28.5, 13.1, 6.73, 1.41, 1.34, 1.18, 1.1, 1.04, 0.8; IR (ATR, cm^{-1}): 3067.1, 2966, 2932.1, 2872.8, 1604, 1473, 1398, 1335, 1310, 1222, 1180, 1053, 1015, 922, 813, 738, 697, 656; elemental analysis calcd (%) for $Mo_2NiC_{56}H_{64}N_{12}Cl_2 \cdot 0.5(CH_2Cl_2)$: C 53.47 H 5.13 N 13.25; Found: C 53.76 H 4.98 N 13.12

General Procedure for the production of $Mo_2M'(dpa)_4Cl_2$ complexes from dioxane: Solid $Mo_2(dpa)_4$ (**7**) was added to a flask with the appropriate $M'Cl_2$ salt (M' = Cr, Fe, Co, or Ni) or $MnCl_2 \cdot 2THF$. The solids were taken up in 1,4-dioxane (30-50 mL) and brought to reflux (140 °C). The reaction mixture was allowed to reflux for 16-72 hours with stirring. The mixture, now darkly colored with most solids dissolved, was allowed to cool to room temperature and volatiles were removed in vacuo. The remaining solids were extracted into CH_2Cl_2 and filtered, leaving behind some darkly colored residue and producing a dark filtrate. The filtrate was collected and reduced to dryness, then the solids were dissolved in minimal CH_2Cl_2 and layered with hexanes to produce crystalline product. Yields and amounts are given below for each compound. Identities of the compounds were confirmed by MALDI mass spectrometry using anthracene as a matrix.

$Mo_2Cr(dpa)_4Cl_2$: 106 mg $Mo_2(dpa)_4$, 19 mg $CrCl_2$, Yield: 28% (32 mg). MS (MALDI, anthracene) expected *m/z* for $[Mo_2Cr(dpa)_4Cl_2]^+$: 994; found 995

$Mo_2Mn(dpa)_4Cl_2$: 112 mg $Mo_2(dpa)_4$, 41 mg $MnCl_2$ 2THF, Yield: 26% (33 mg). MS (MALDI, anthracene) expected *m/z* for $[Mo_2Mn(dpa)_4Cl]^+$: 961.4; found 962

$Mo_2Fe(dpa)_4Cl_2$: 115 mg $Mo_2(dpa)_4$, 18 mg $FeCl_2$, Yield: 74% (85 mg). MS (MALDI, anthracene) expected *m/z* for $[Mo_2Fe(dpa)_4Cl]^+$: 961.5; found 963

$Mo_2Co(dpa)_4Cl_2$: 105 mg $Mo_2(dpa)_4$, 16 mg $CoCl_2$, Yield: 63% (72 mg). MS (MALDI, anthracene) expected *m/z* for $[Mo_2Co(dpa)_4Cl]^+$: 965.5; found 966

$Mo_2Ni(dpa)_4Cl_2$: 153 mg $Mo_2(dpa)_4$, 40 mg $NiCl_2$, Yield: 93% (160 mg). MS (MALDI, anthracene) expected *m/z* for $[Mo_2Ni(dpa)_4Cl_2]^+$: 1001; found 1002

Collected IR Spectra

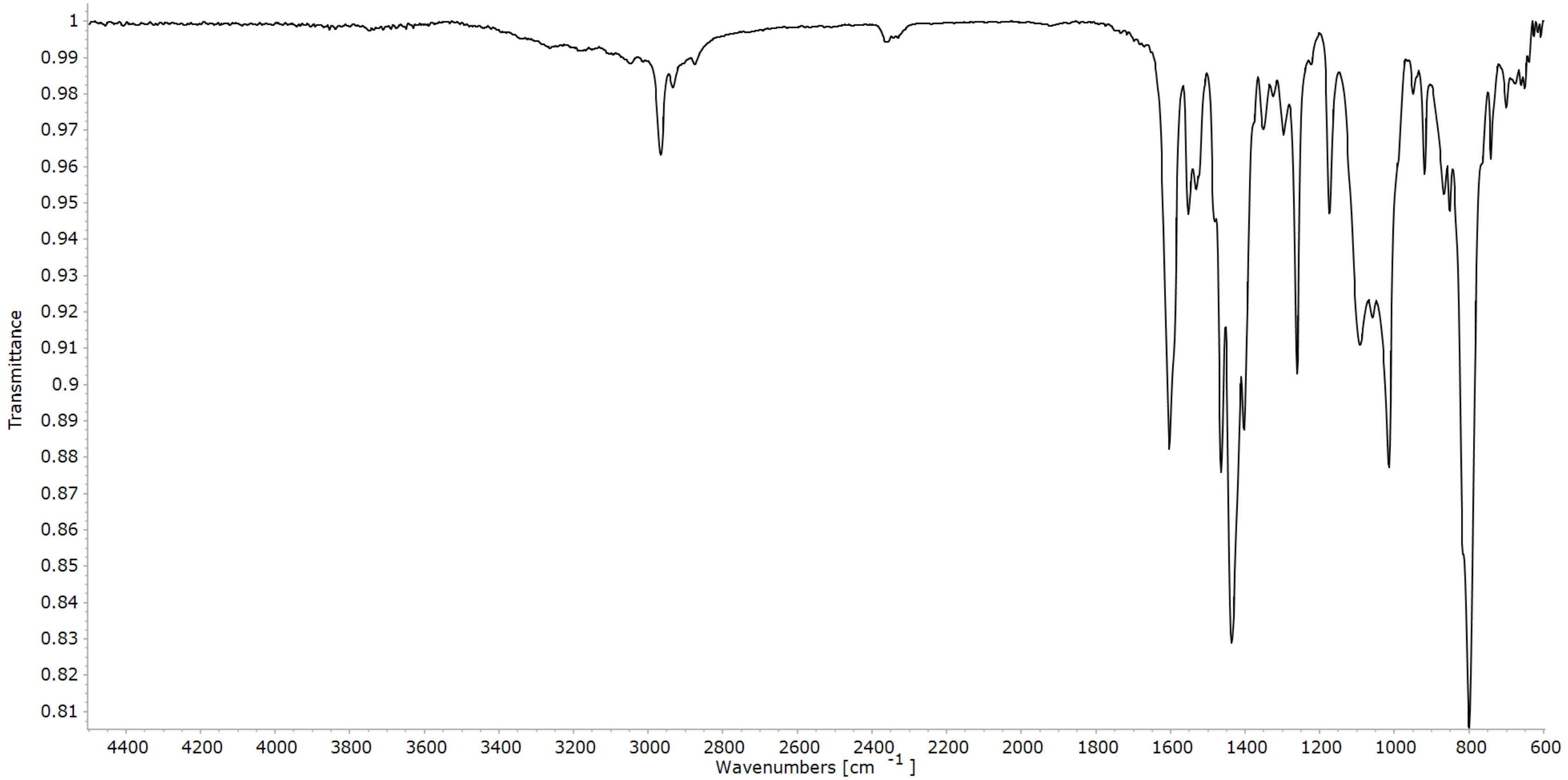

Figure S1: IR spectrum of **1** taken in ATR mode on a powder sample

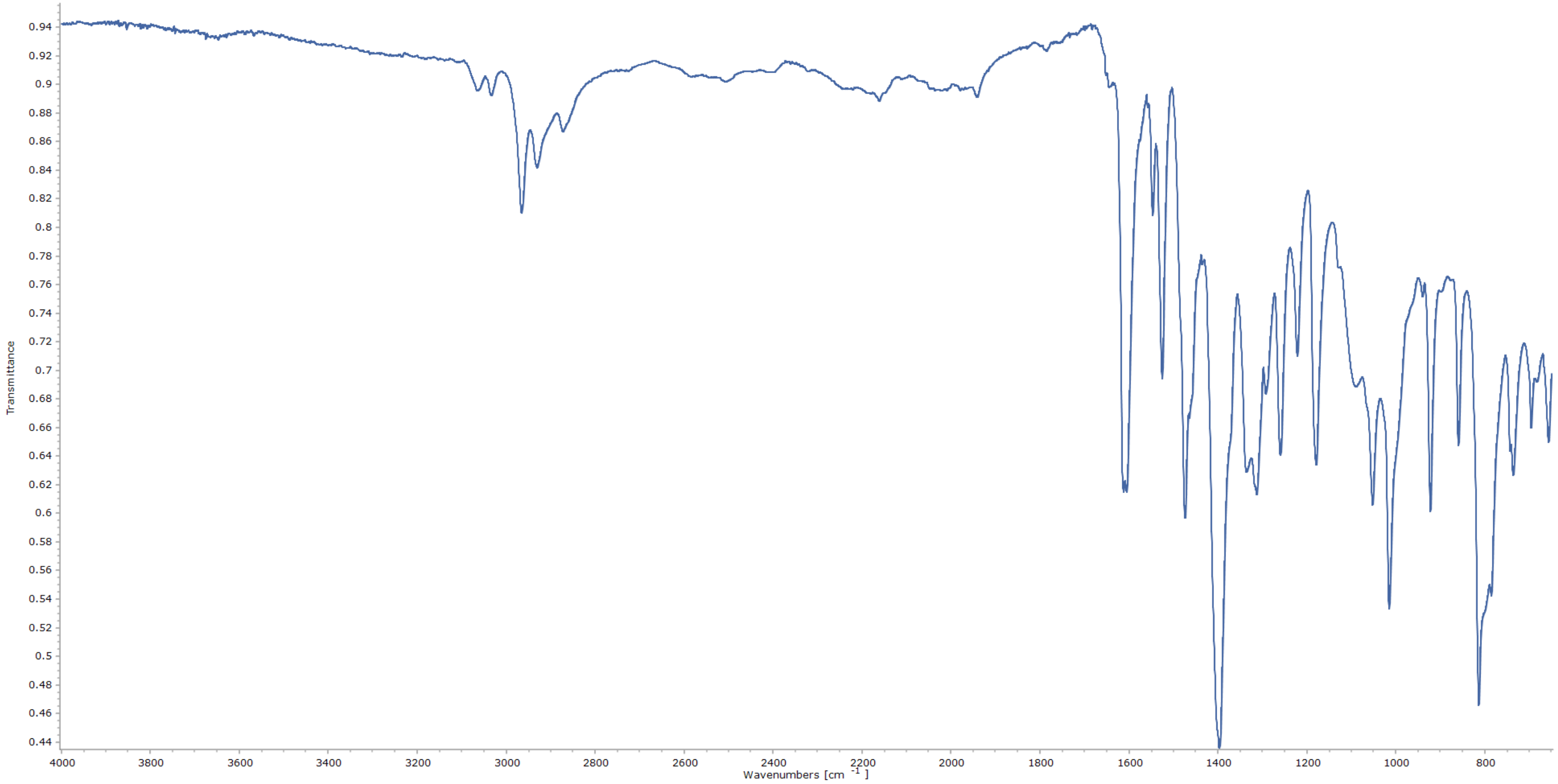

Figure S2: IR spectrum of **2** taken in ATR mode on a powder sample

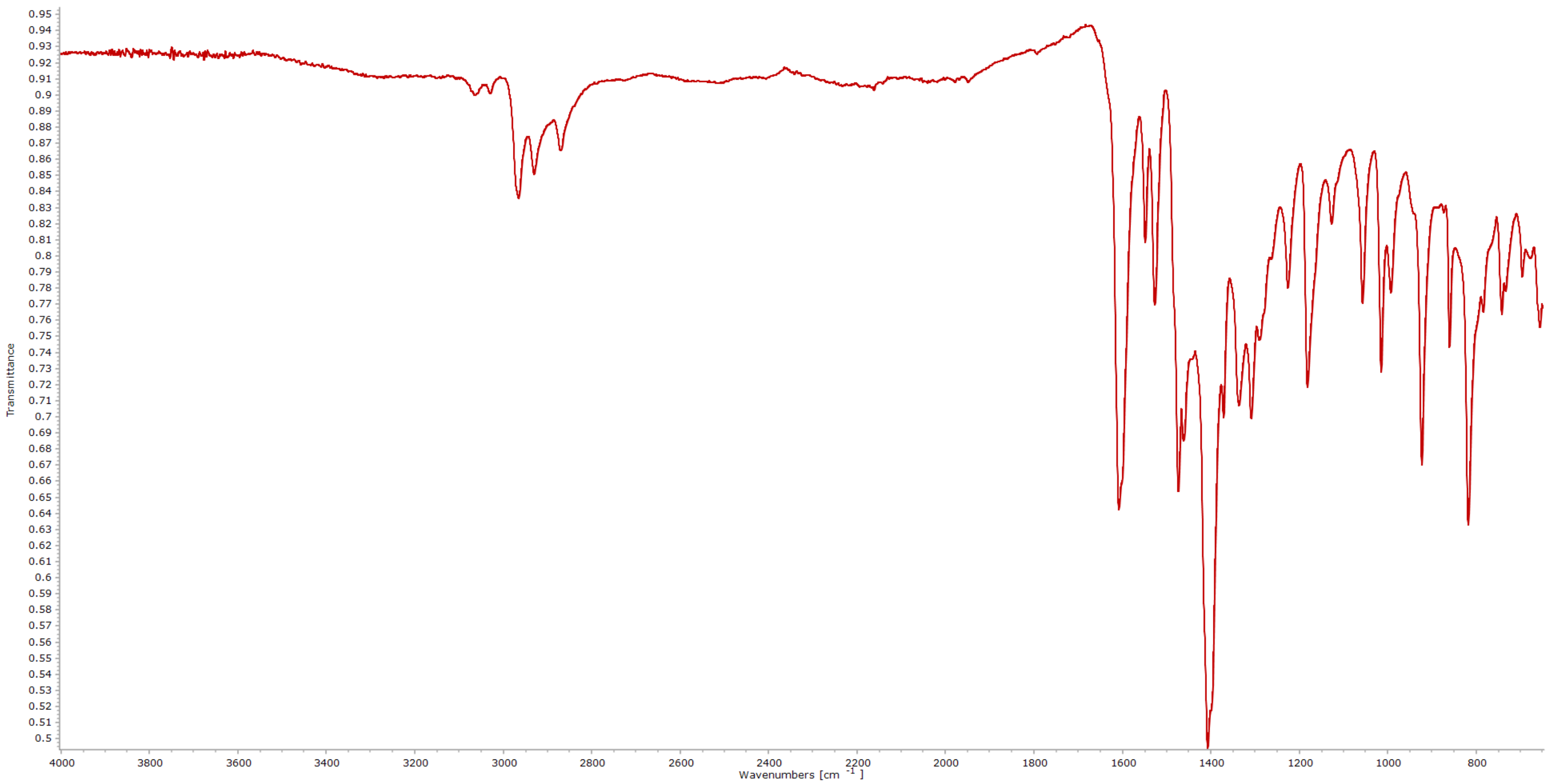

Figure S3: IR spectrum of **3** taken in ATR mode on a powder sample

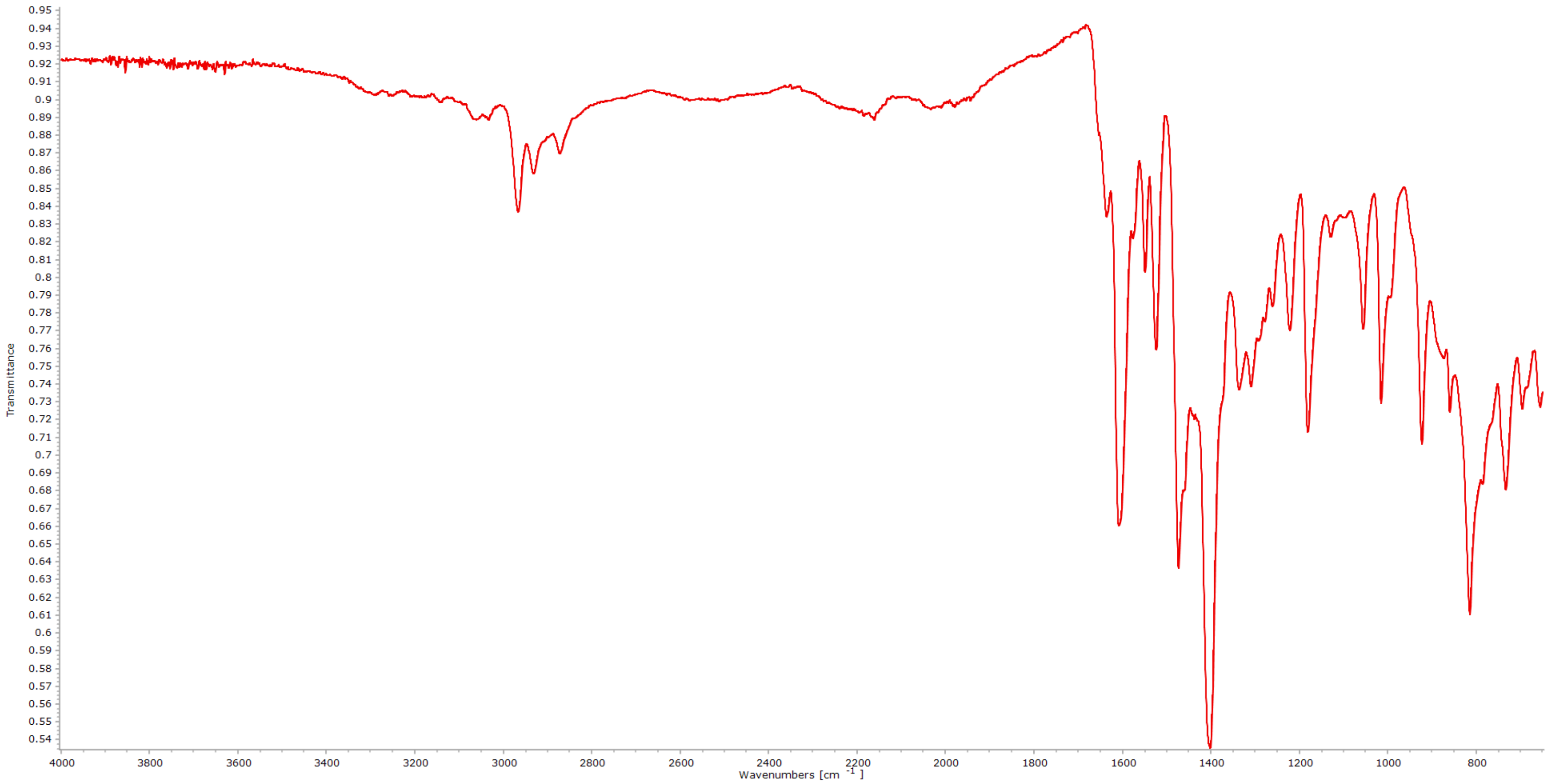

Figure S4: IR spectrum of **4** taken in ATR mode on a powder sample

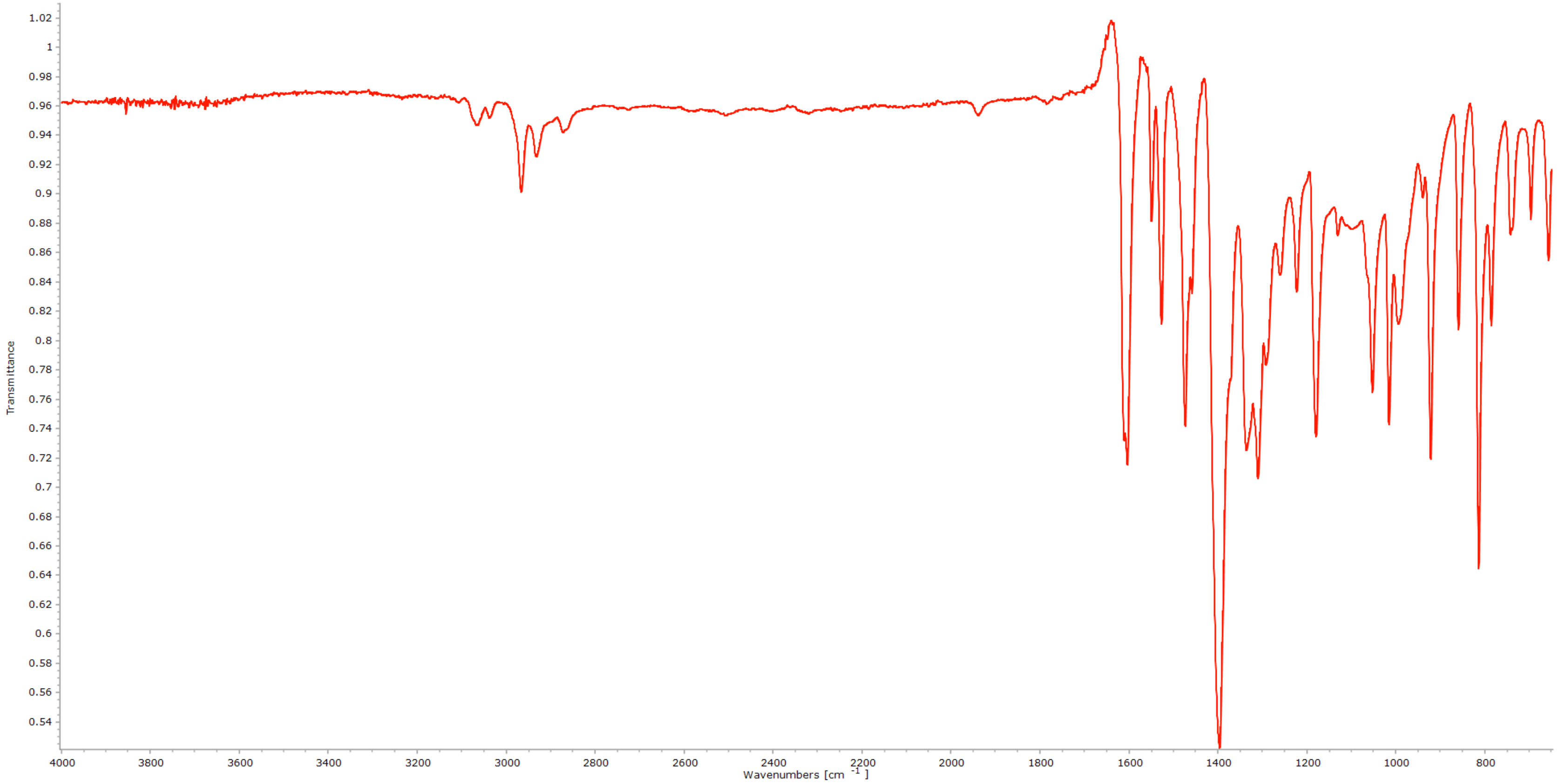

Figure S5: IR spectrum of **5** taken in ATR mode on a powder sample

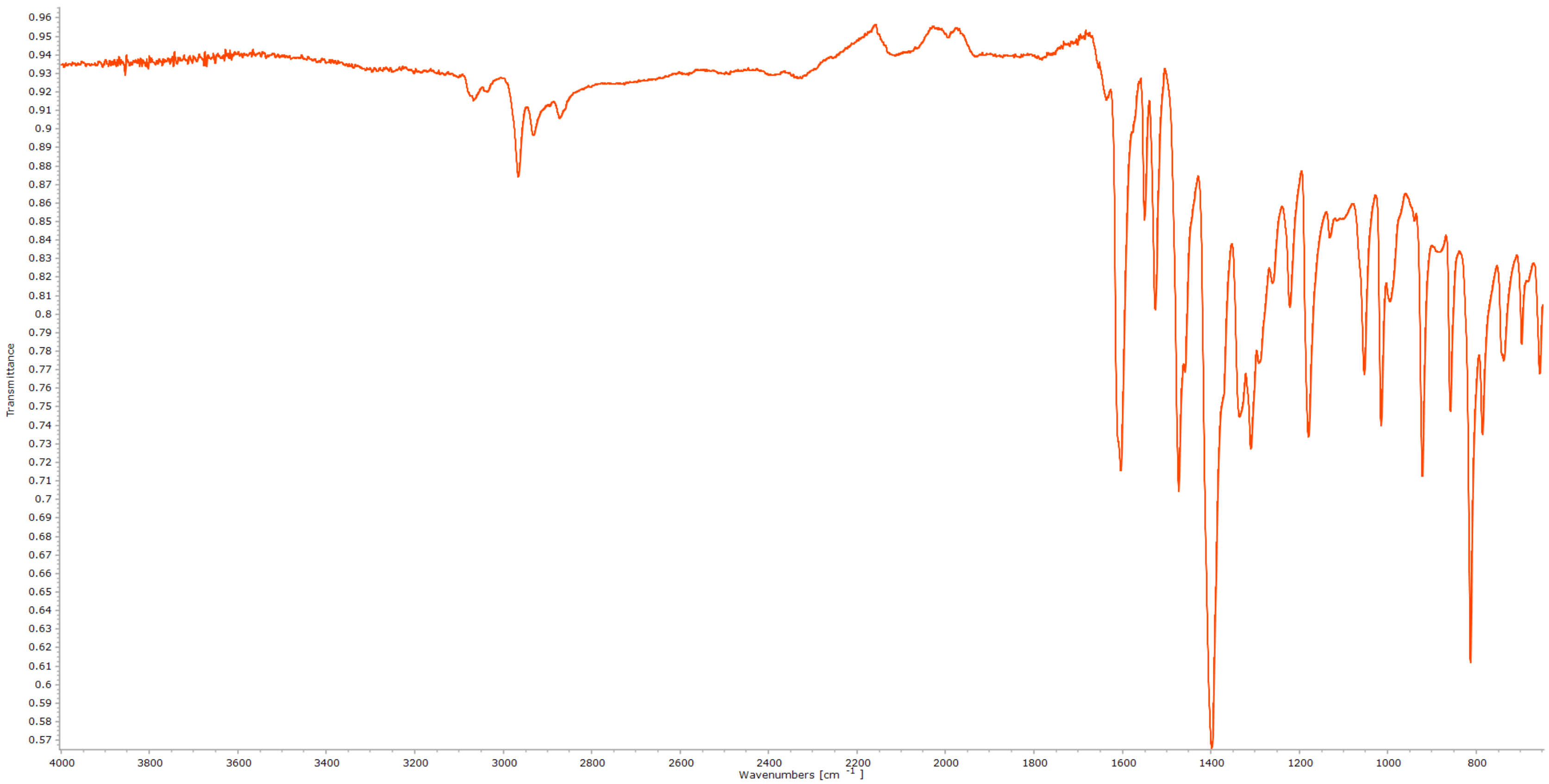

Figure S6: IR spectrum of **6** taken in ATR mode on a powder sample

Collected NMR Spectra:

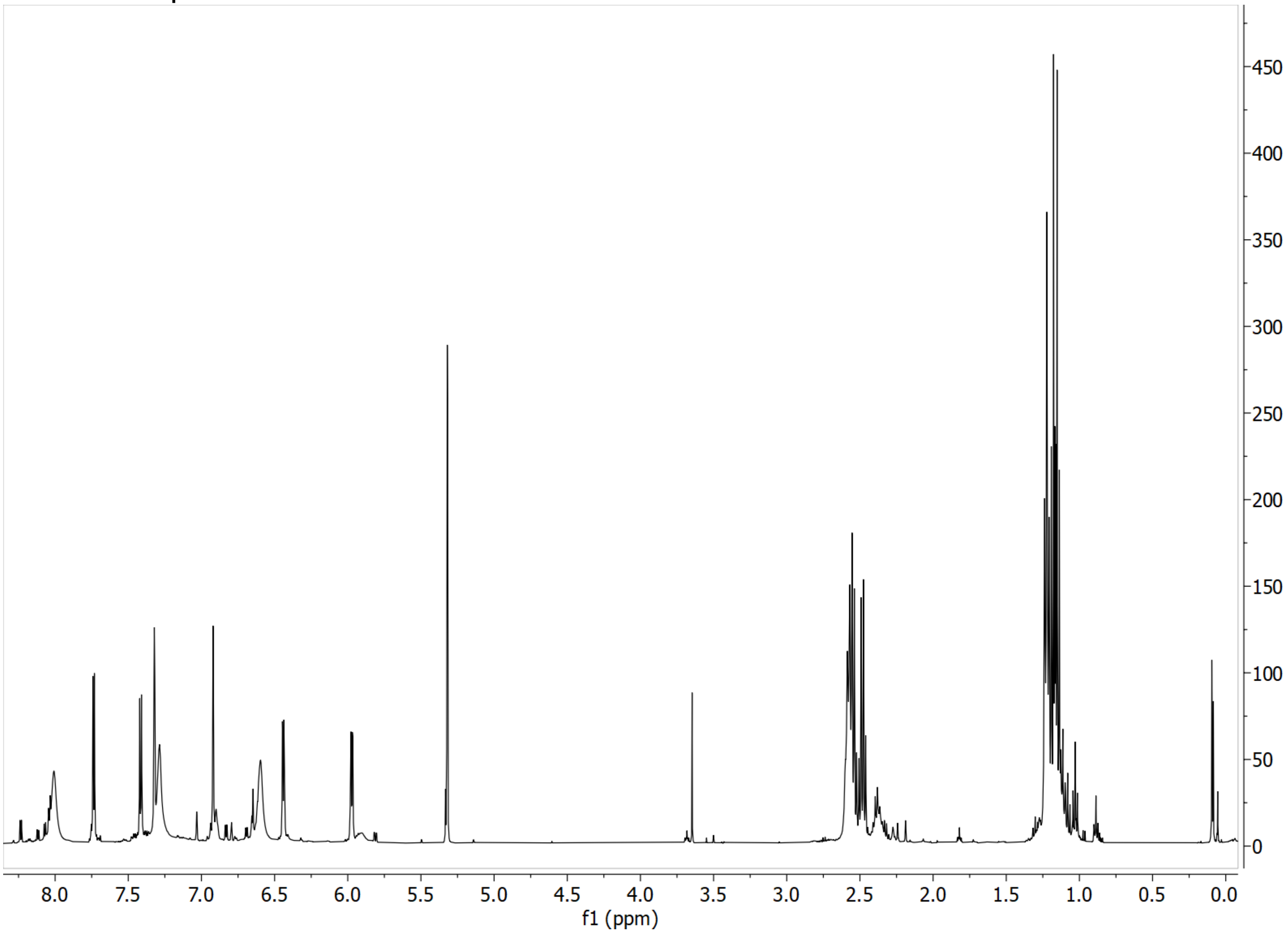

Figure S7: Full ^{1}H NMR spectrum of **1** taken in dry CD_2Cl_2 on a 500 MHz spectrometer at room temperature.

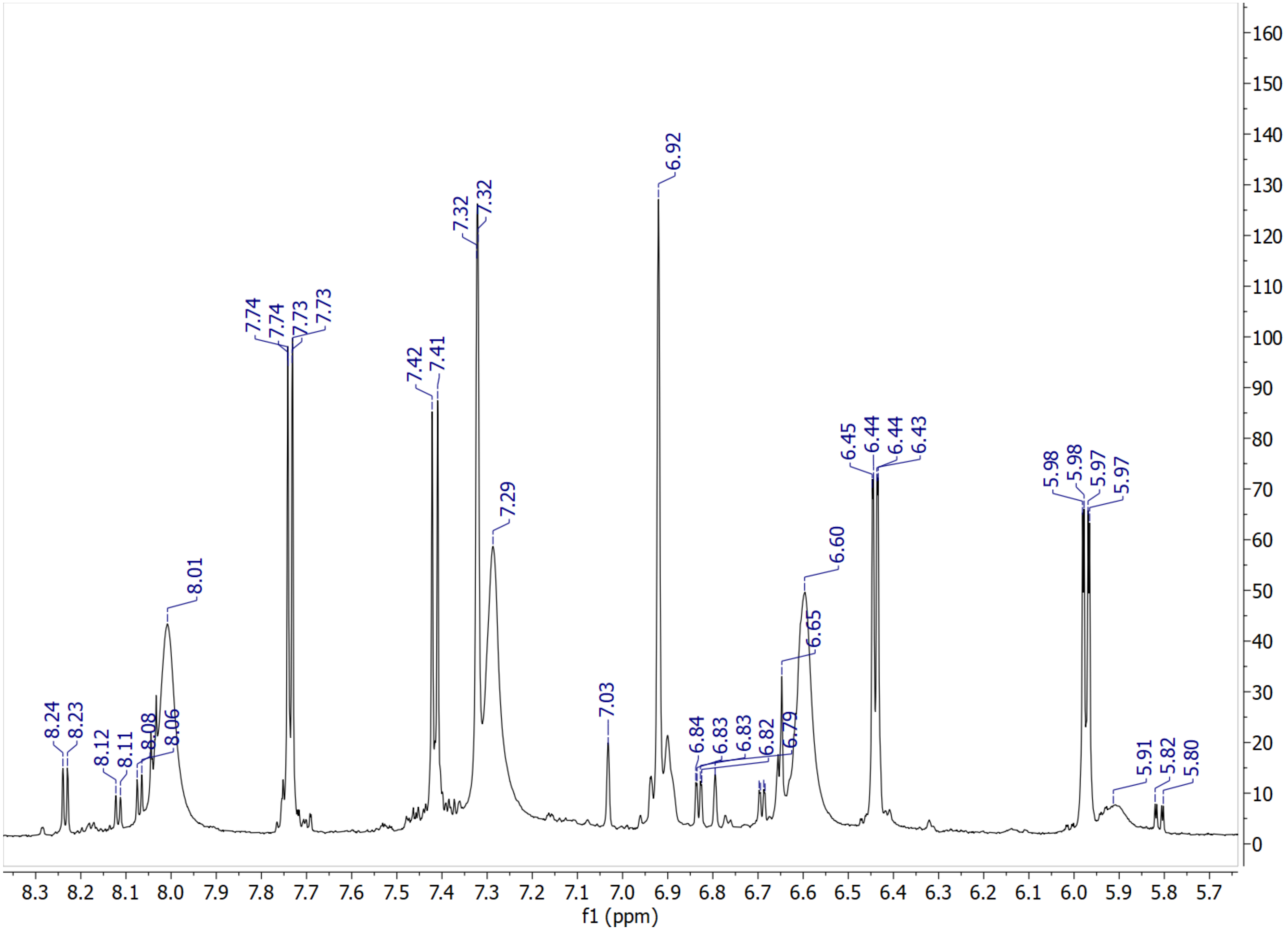

Figure S8: A zoom in of the aromatic region of the room temperature NMR spectrum of **1** in CD_2Cl_2 taken on a 500 MHz spectrometer.

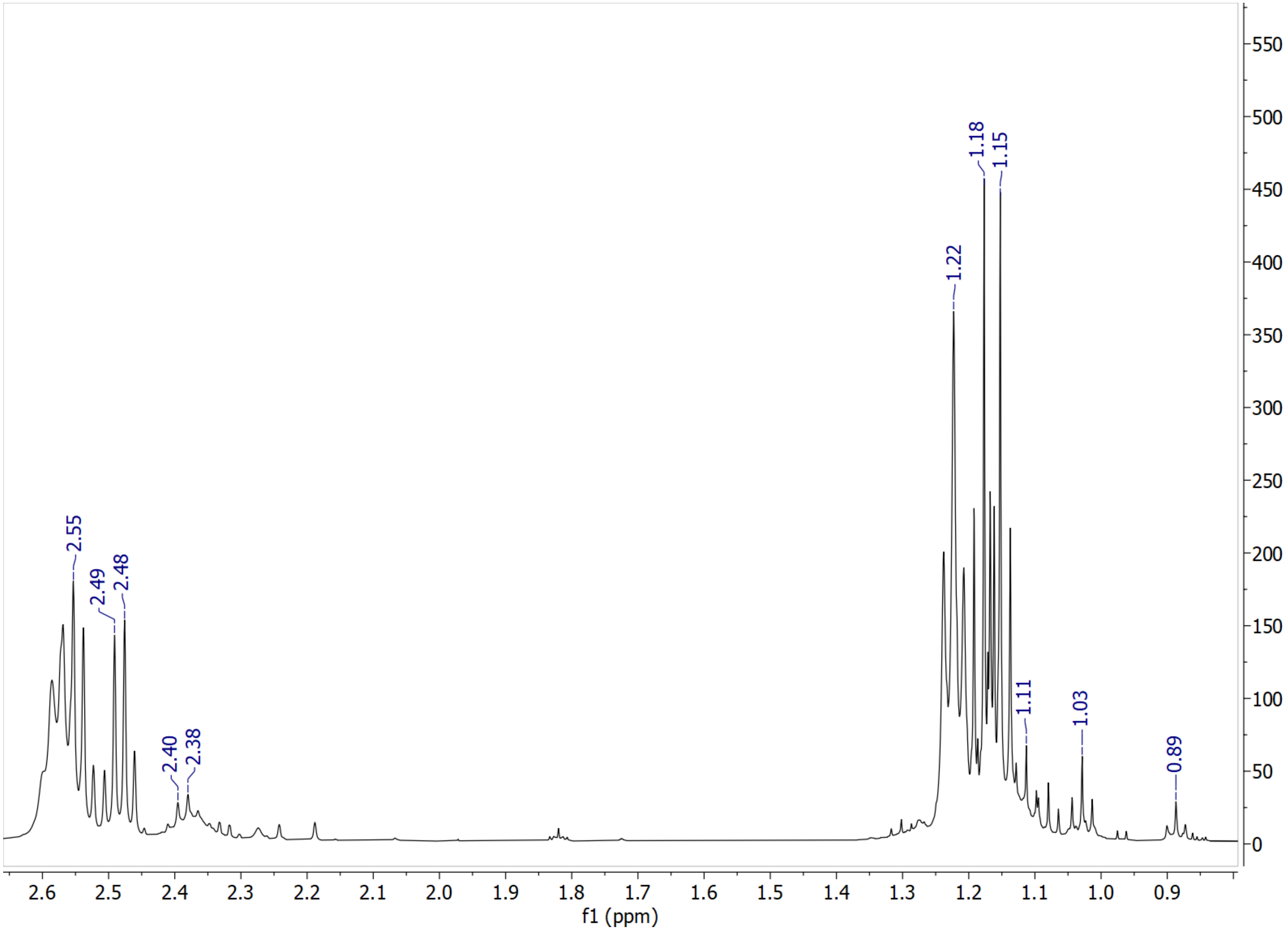

Figure S9: A zoom in of the CH_2CH_3 region of the room temperature NMR spectrum of **1** in CD_2Cl_2 taken on a 500 MHz spectrometer.

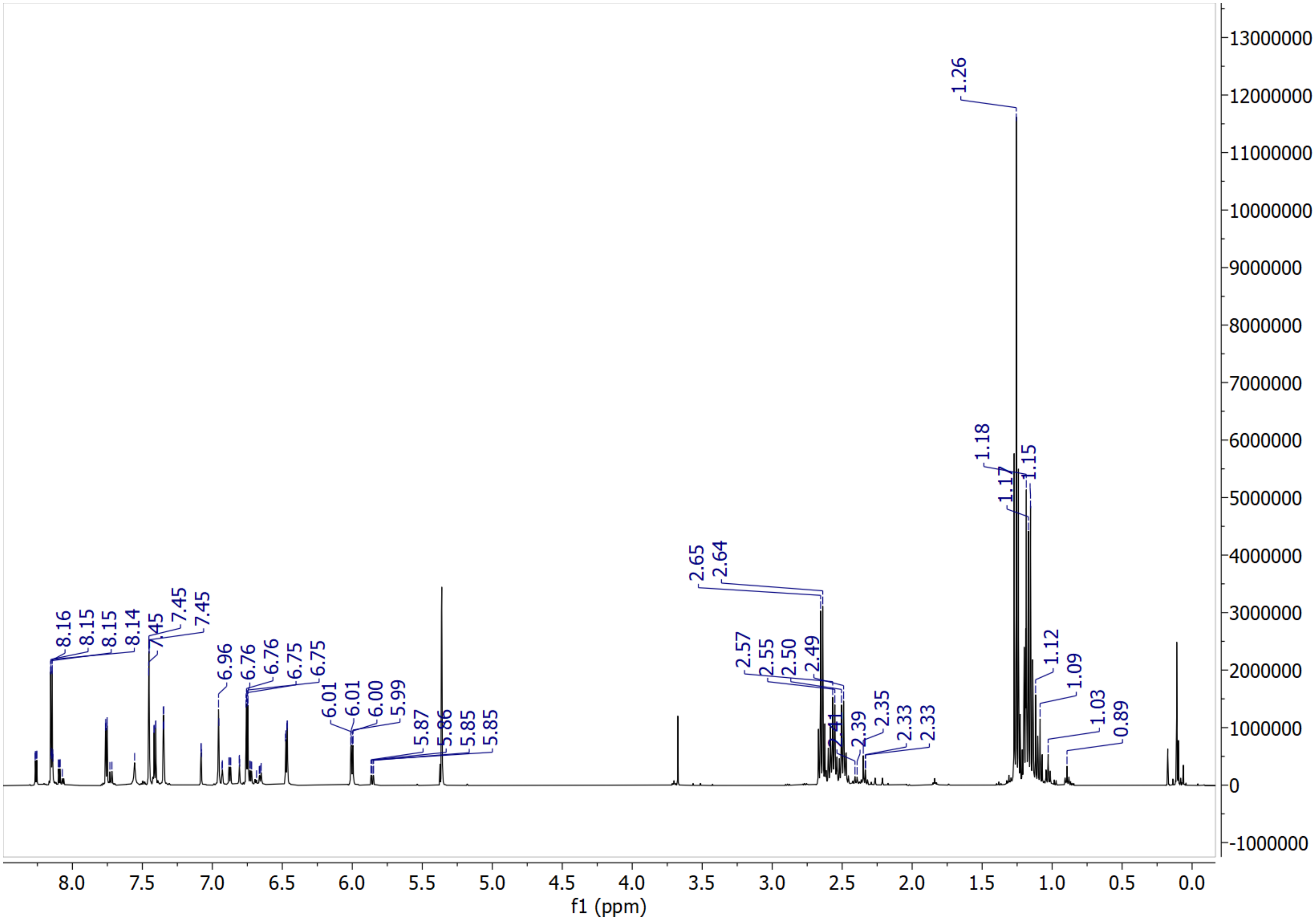

Figure S10: Full NMR spectrum of **1** taken in dry CD_2Cl_2 on a 500 MHz spectrometer at –20 °C.

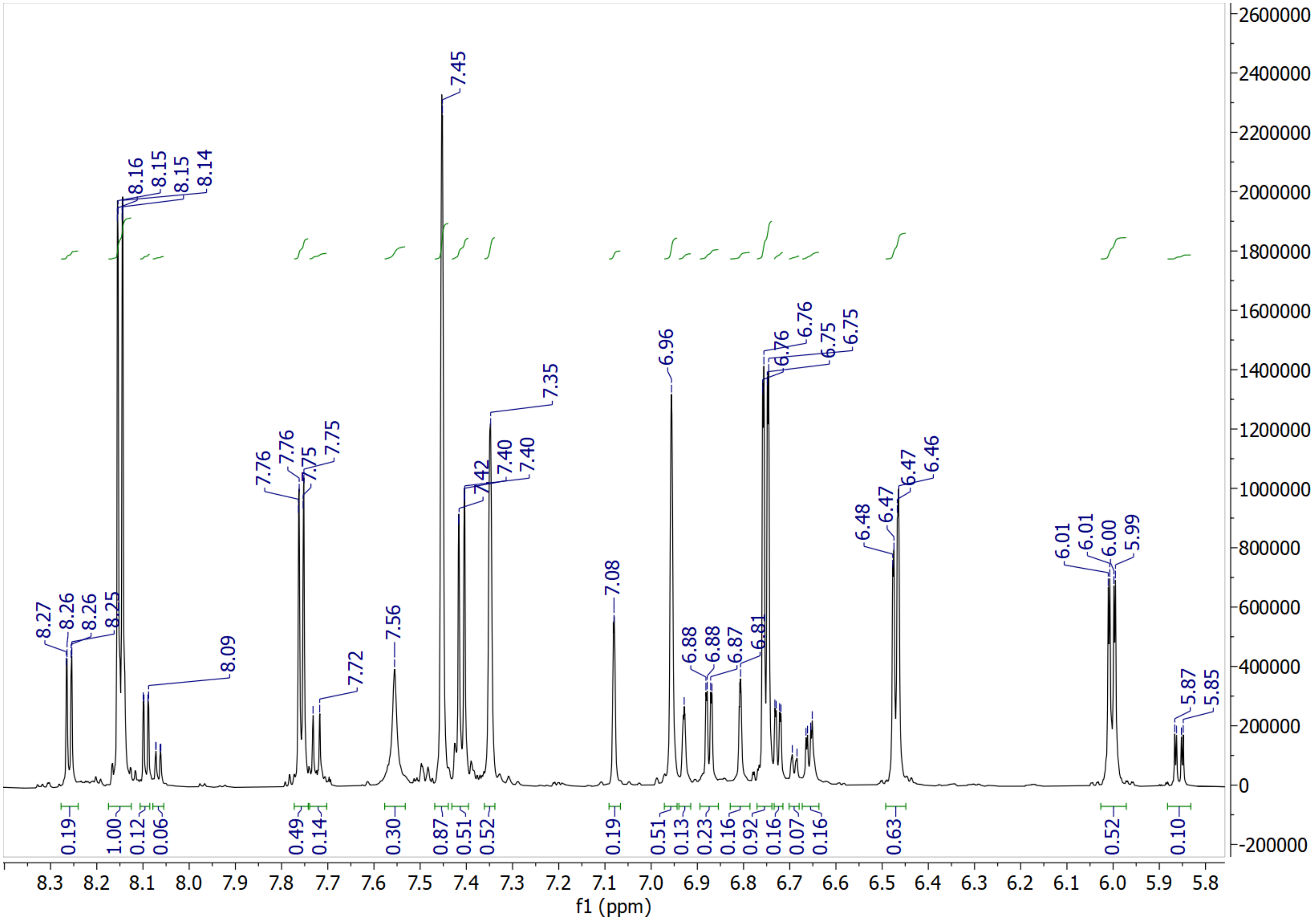

Figure S11: Zoom in of the aromatic region of the NMR spectrum of **1** taken in dry CD_2Cl_2 on a 500 MHz spectrometer at –20 °C.

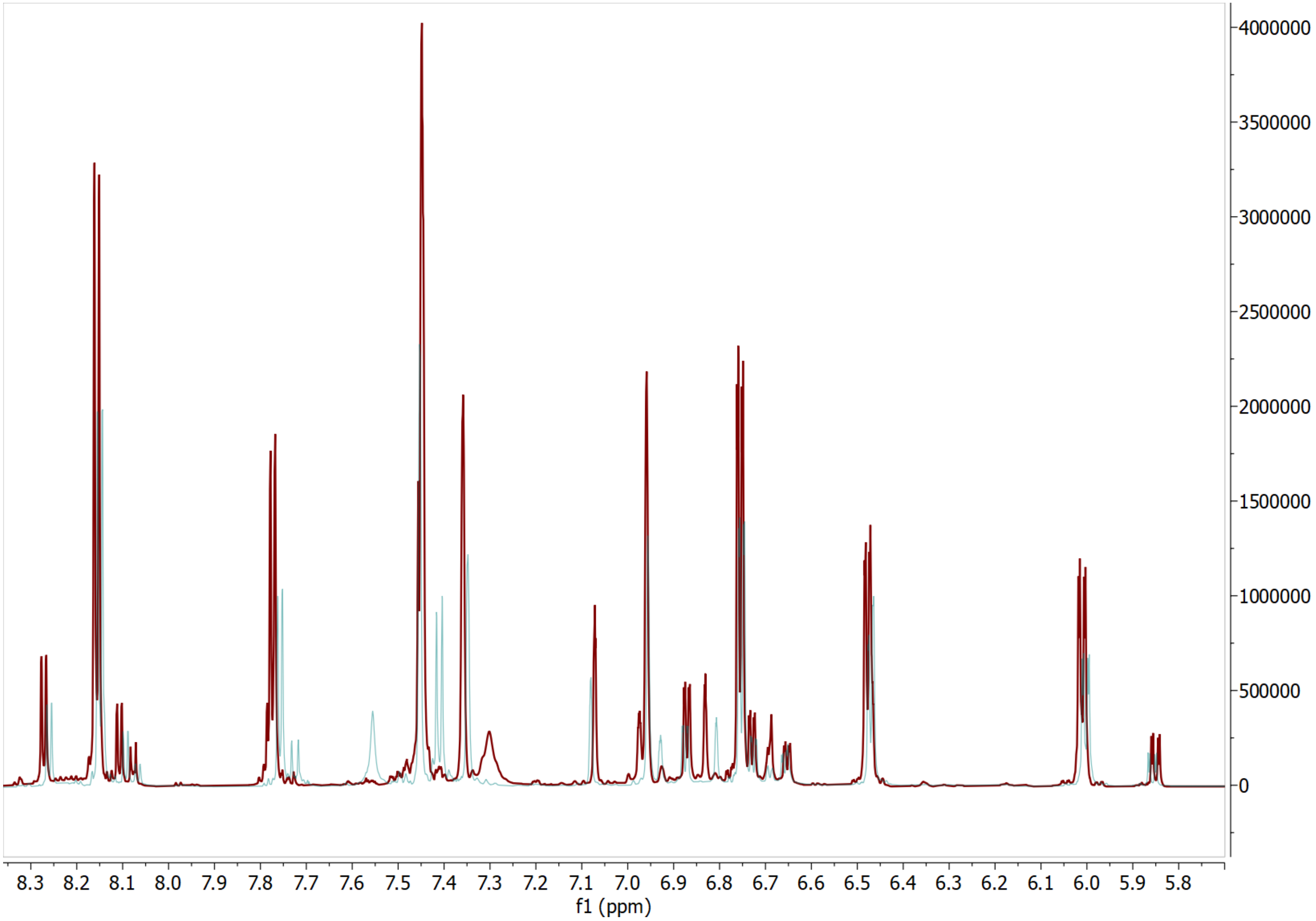

Figure S12: An overlay of the aromatic region of **1** at room temperature and –20 °C.

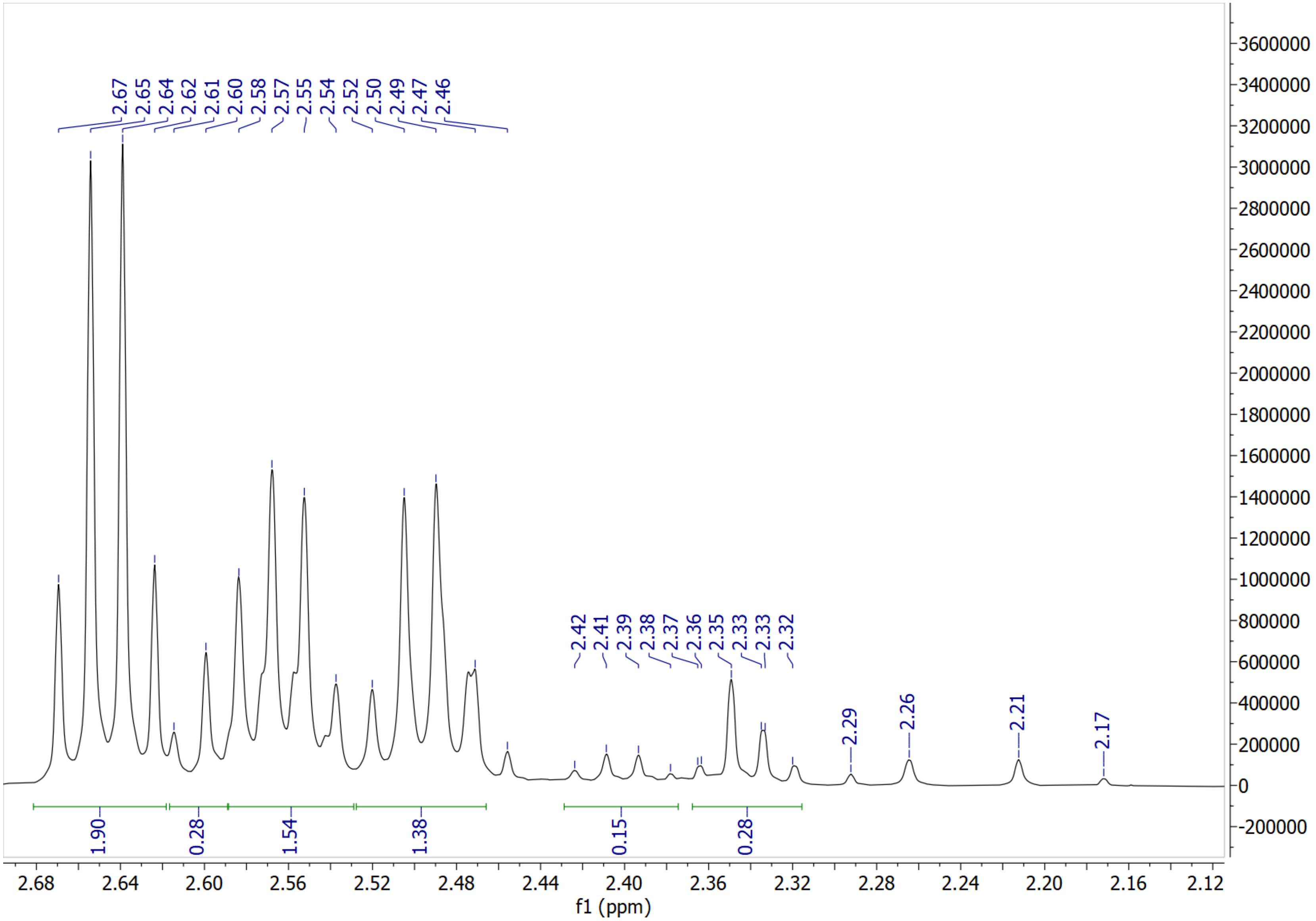

Figure S13: Zoom in of the CH_2 groups of the CH_2CH_3 region of the NMR spectrum of **1** taken in dry CD_2Cl_2 on a 500 MHz spectrometer at –20 °C

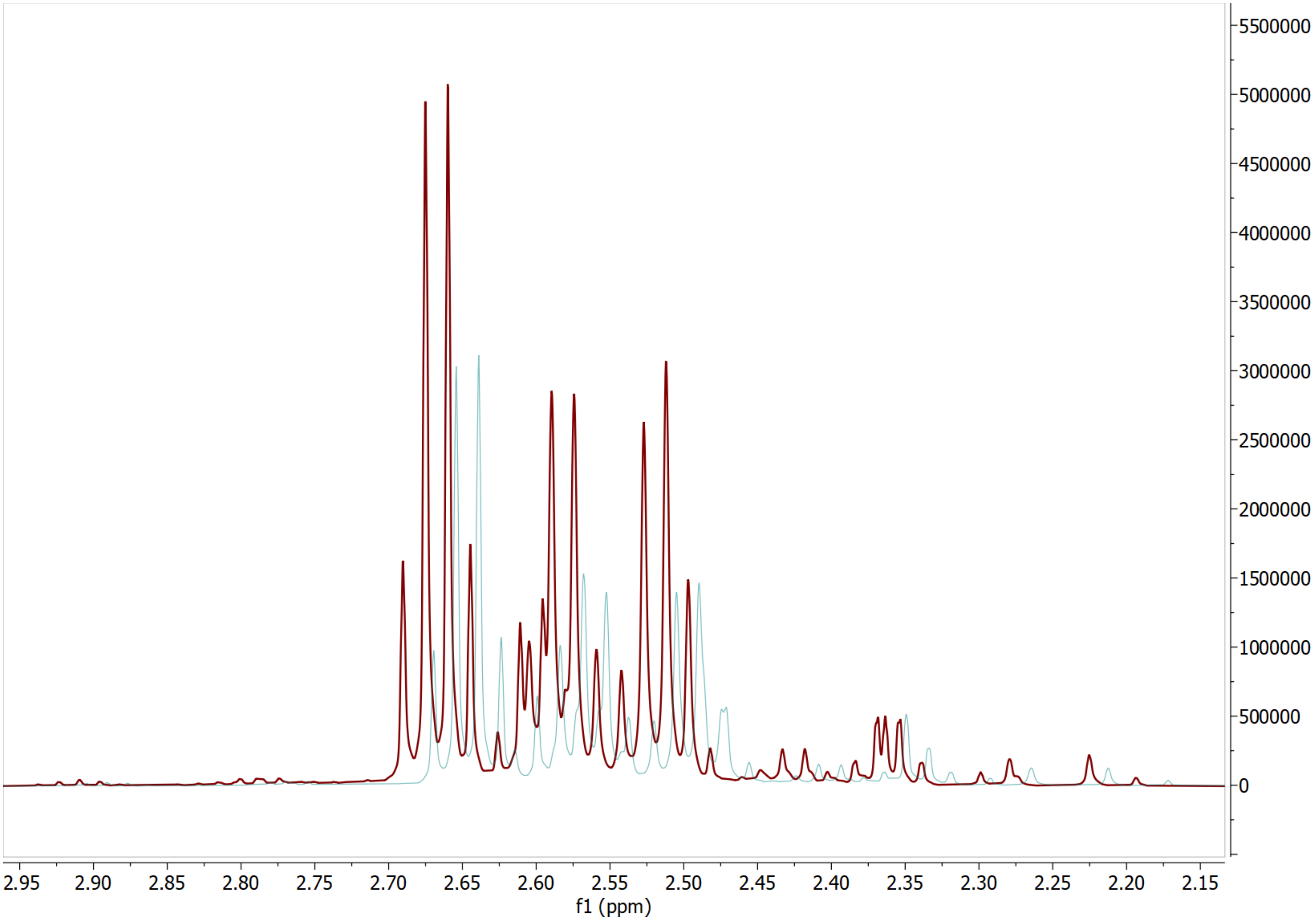

Figure S14: An overlay of the CH_2 region of the CH_2CH_3 groups of **1** at room temperature and –20 °C.

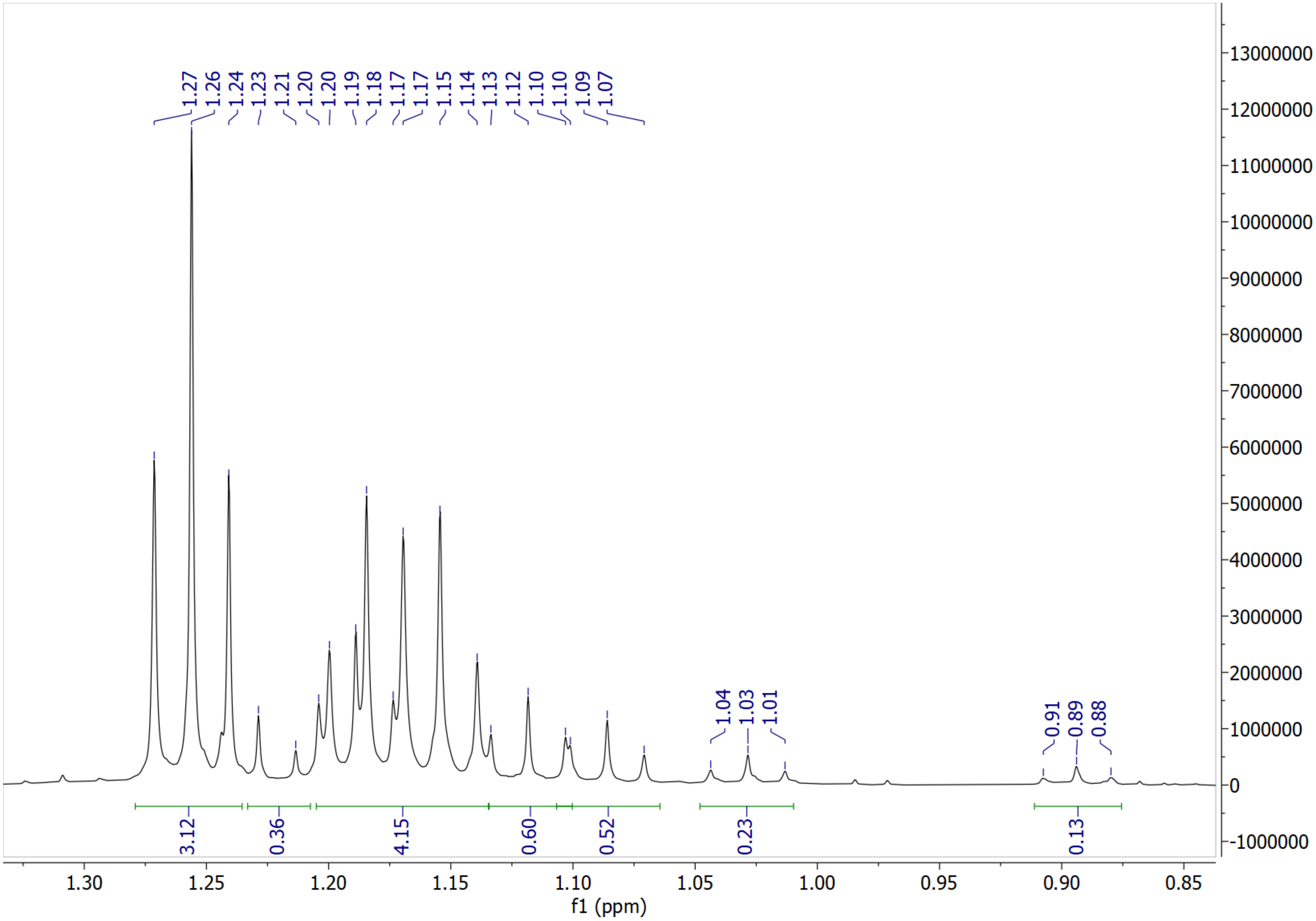

Figure S15: Zoom in of the CH_3 groups of the CH_2CH_3 region of the NMR spectrum of **1** taken in dry CD_2Cl_2 on a 500 MHz spectrometer at –20 °C

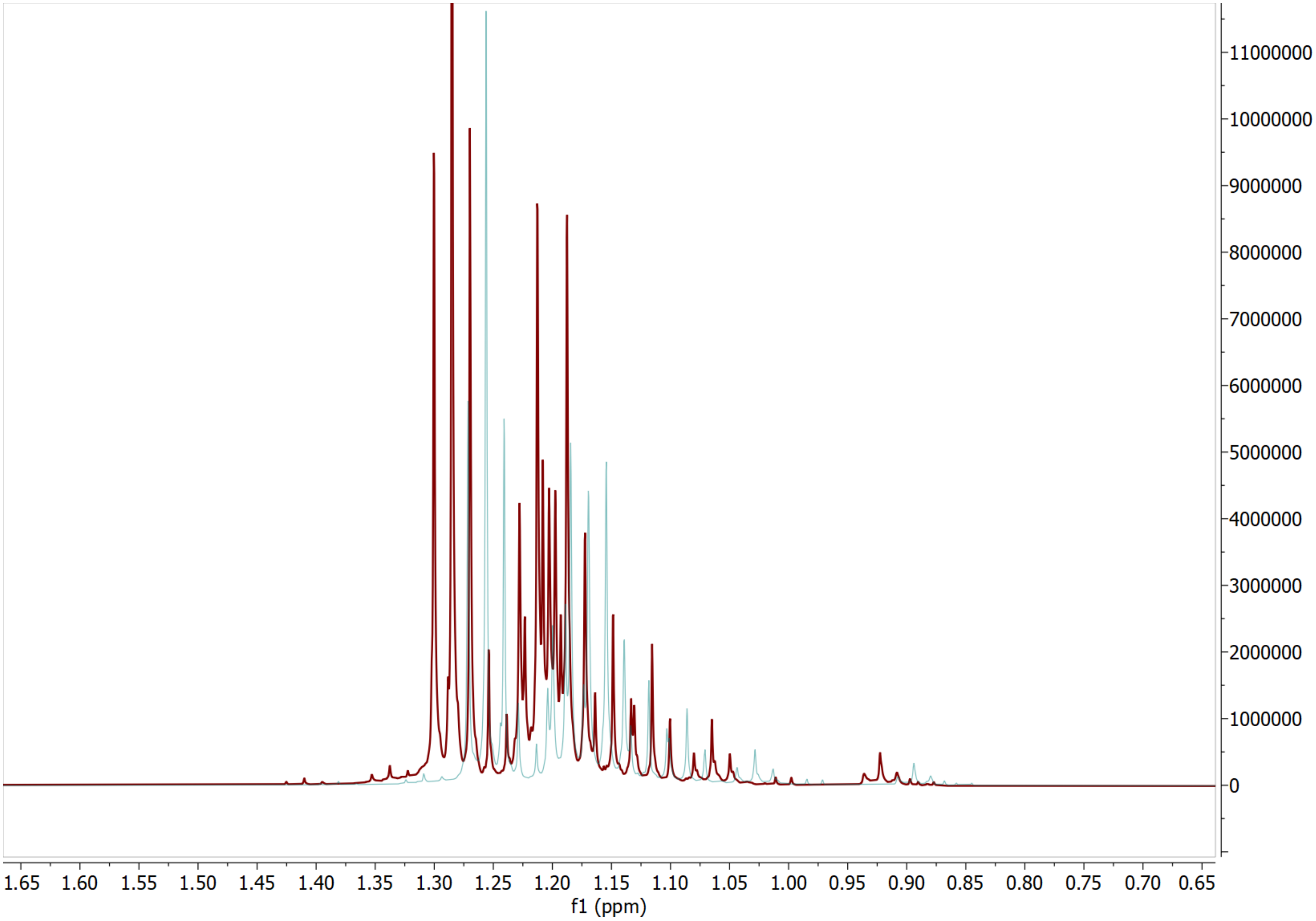

Figure S16: An overlay of the CH_3 region of the CH_2CH_3 groups of **1** at room temperature and –20 °C

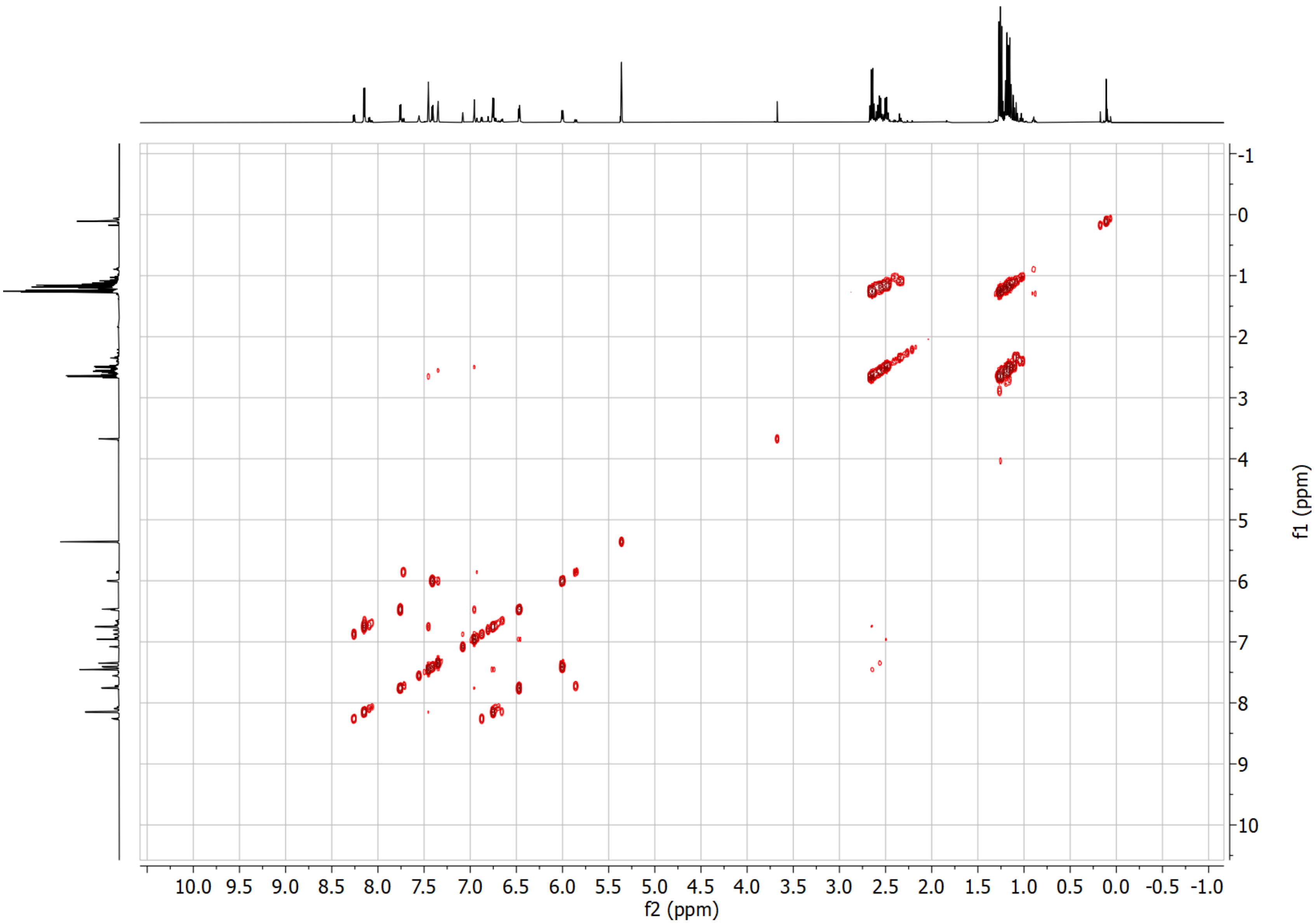

Figure S17: A 2D COSY spectrum of **1** taken in CD_2Cl_2 at –20 °C on a 500 MHz spectrometer.

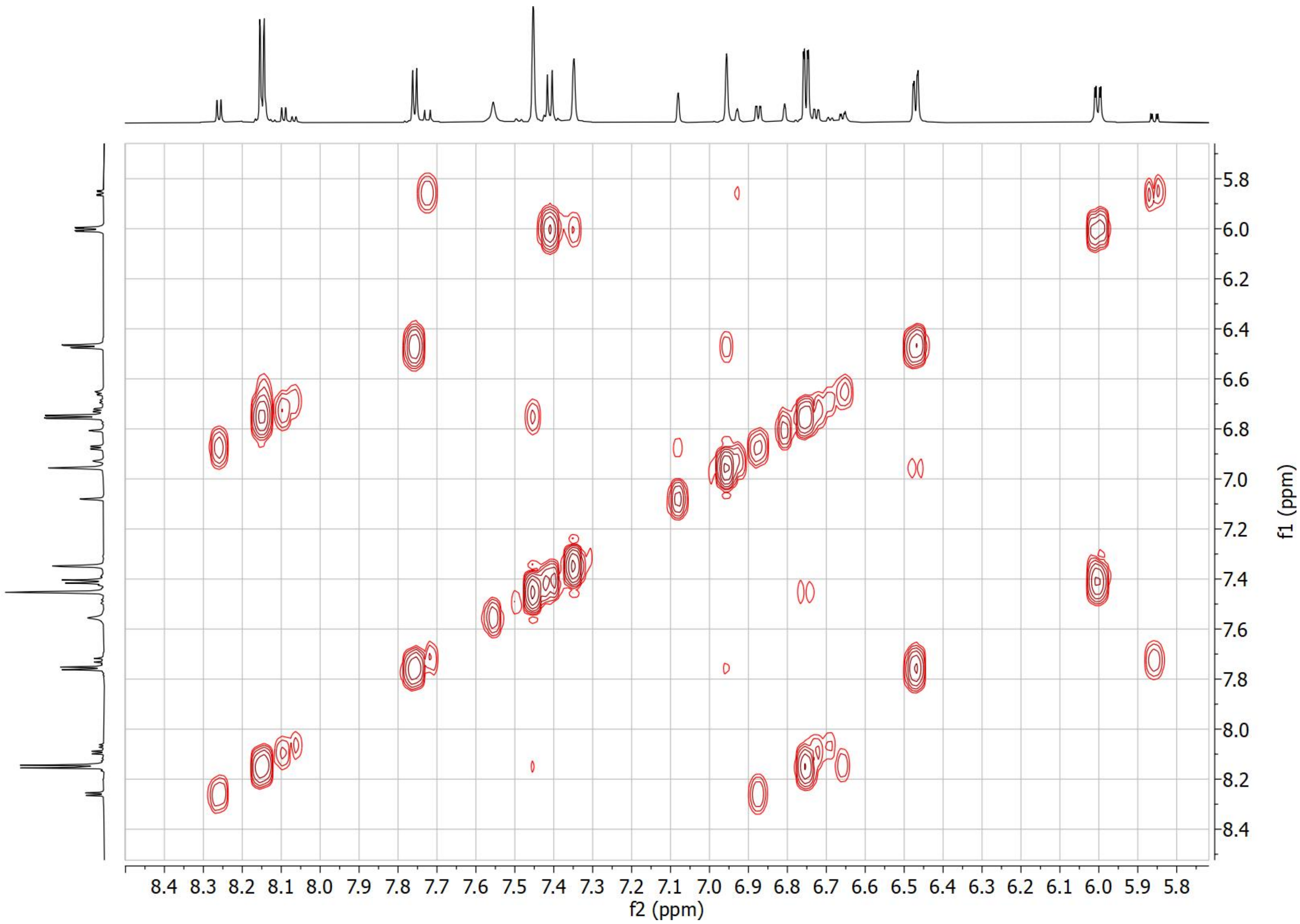

Figure S18: A zoom in of the correlations in the aromatic region of the COSY of **1** in CD_2Cl_2 at –20 °C.

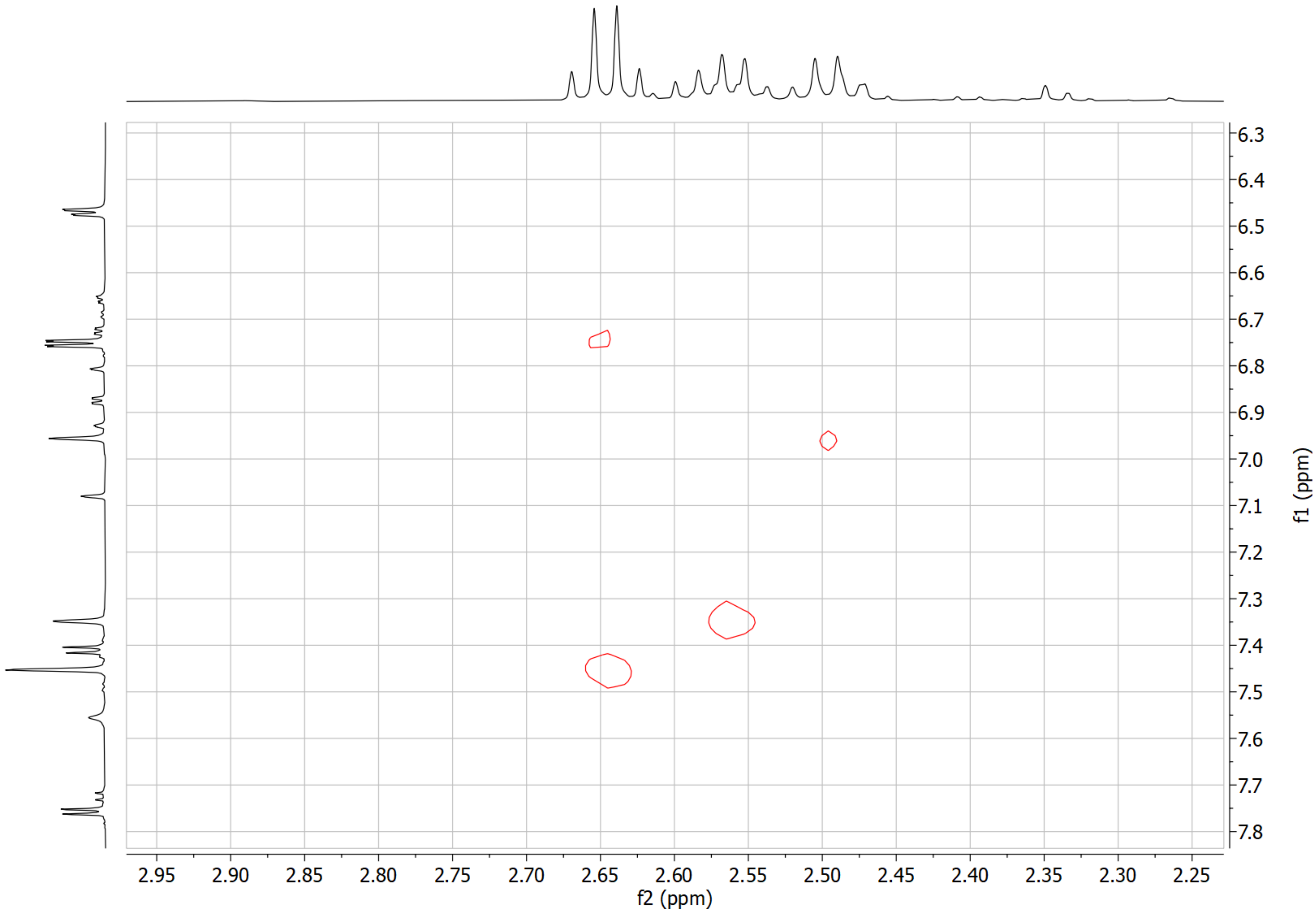

Figure S19: A zoom in of the correlations between the CH_2 ethyl protons and the aromatic protons of **1**.

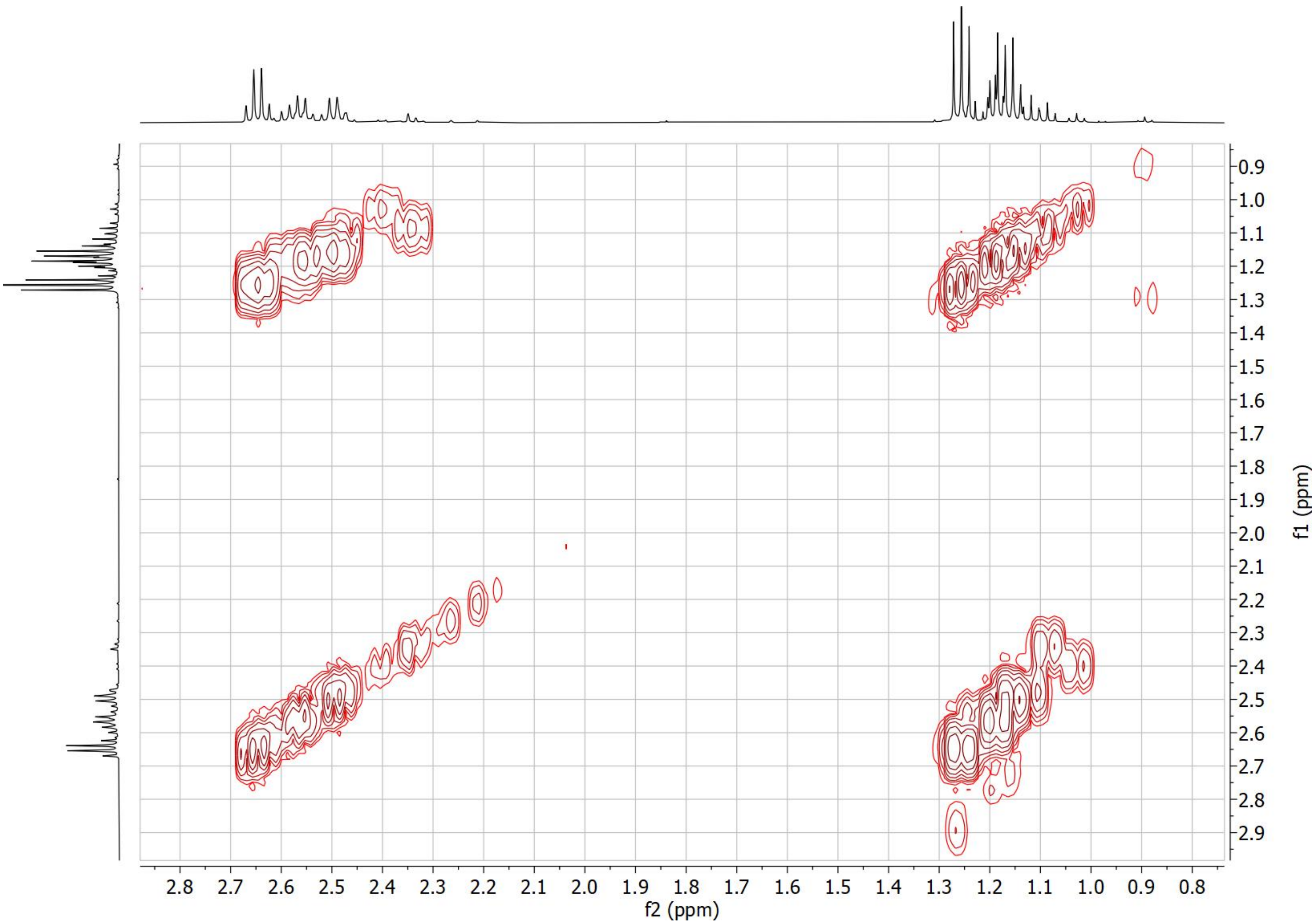

Figure S20: A zoom in of the correlations between the CH_2 ethyl protons and the CH_3 ethyl protons of **1**. Some details in this region cannot be distinguished due to the overlapping peaks.

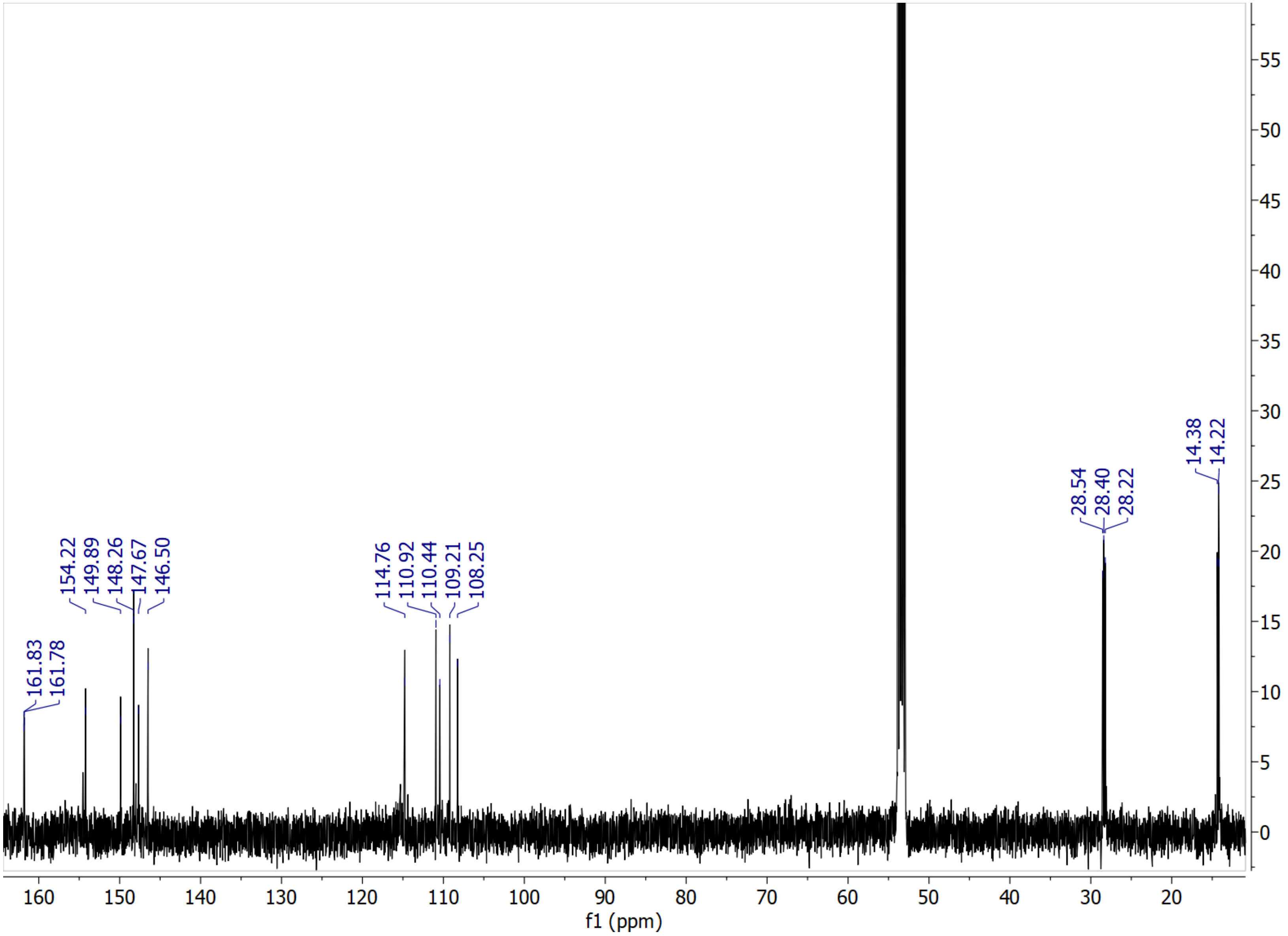

Figure S21: ^{13}C NMR spectrum of 1 taken on a 500 MHz spectrometer at room temperature in CD_2Cl_2

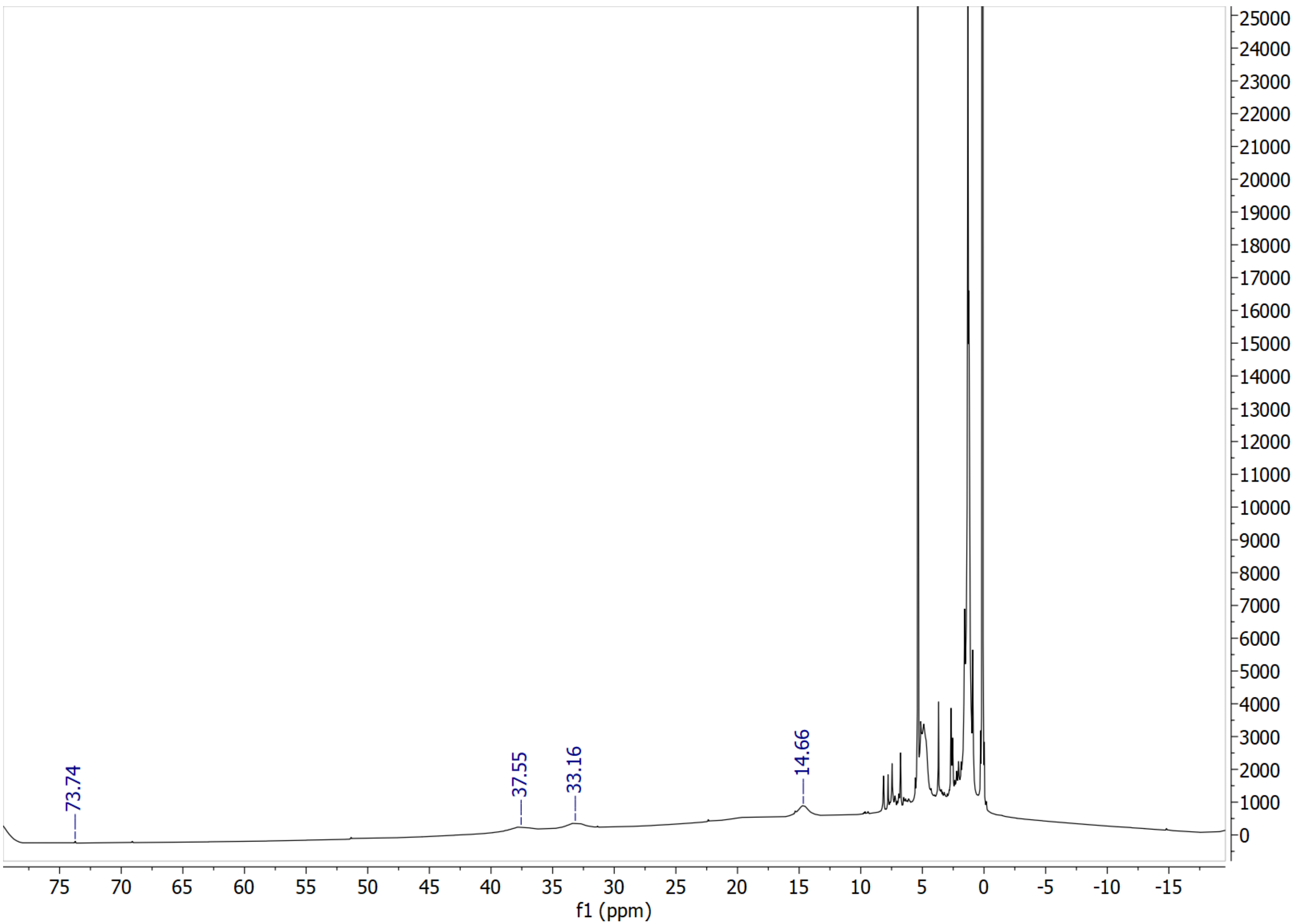

Figure S22: Full NMR spectrum of **2** taken in dry CD_2Cl_2 on a 500 MHz spectrometer

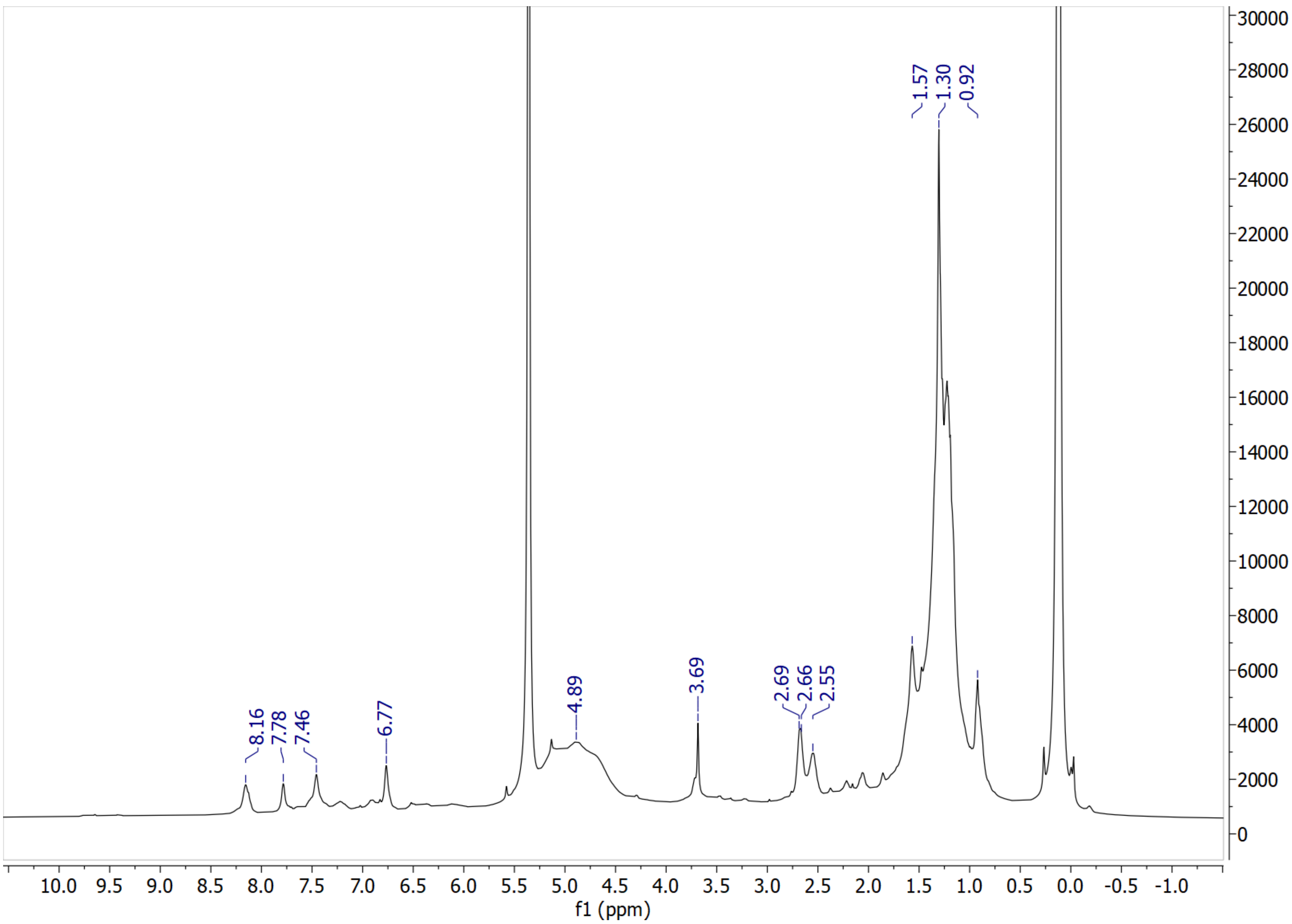

Figure S23: Zoomed in NMR spectrum of **2** taken in dry CD_2Cl_2 on a 500 MHz spectrometer

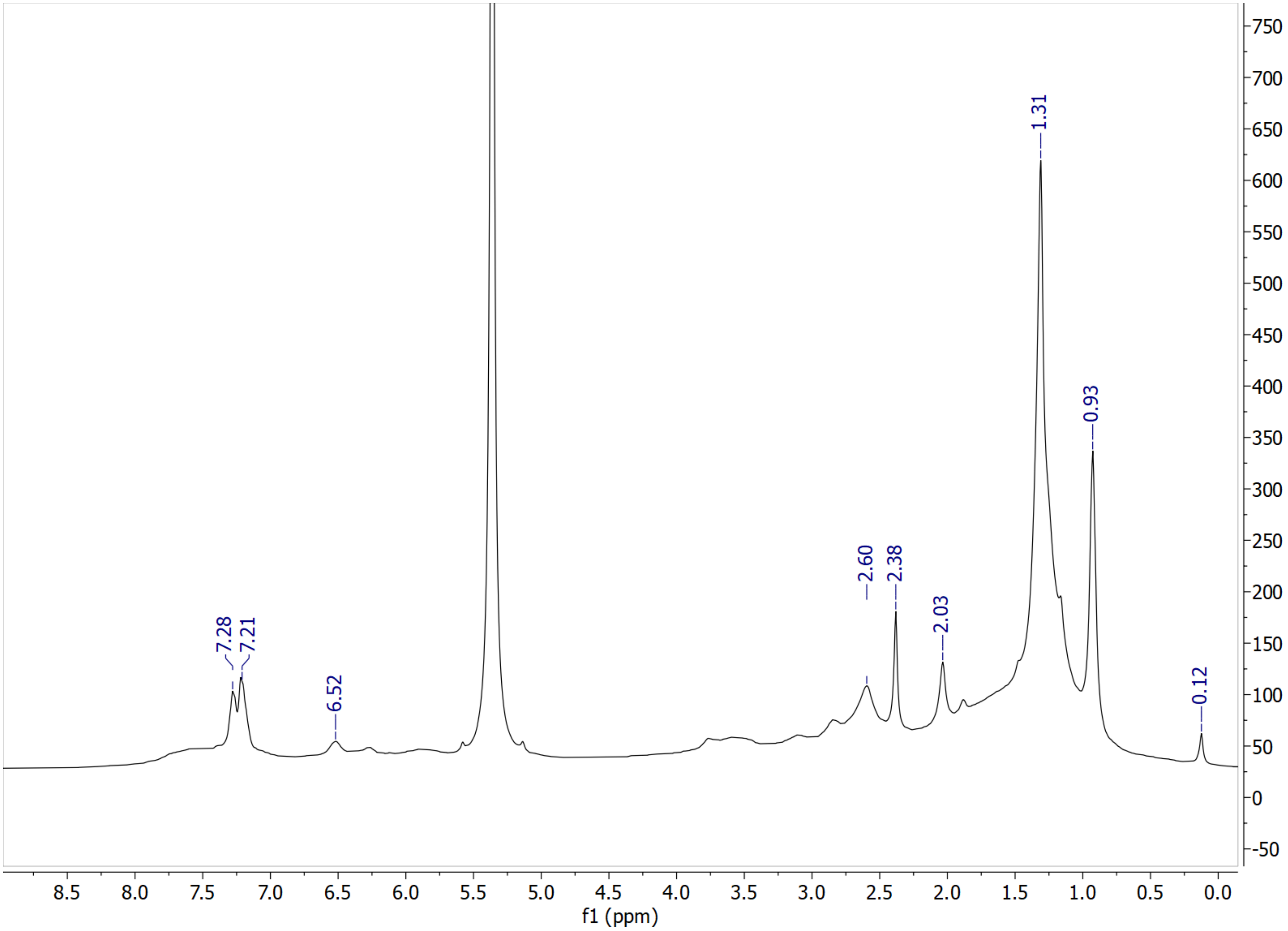

Figure S24: Full NMR spectrum of **3** taken in dry CD_2Cl_2 on a 500 MHz spectrometer

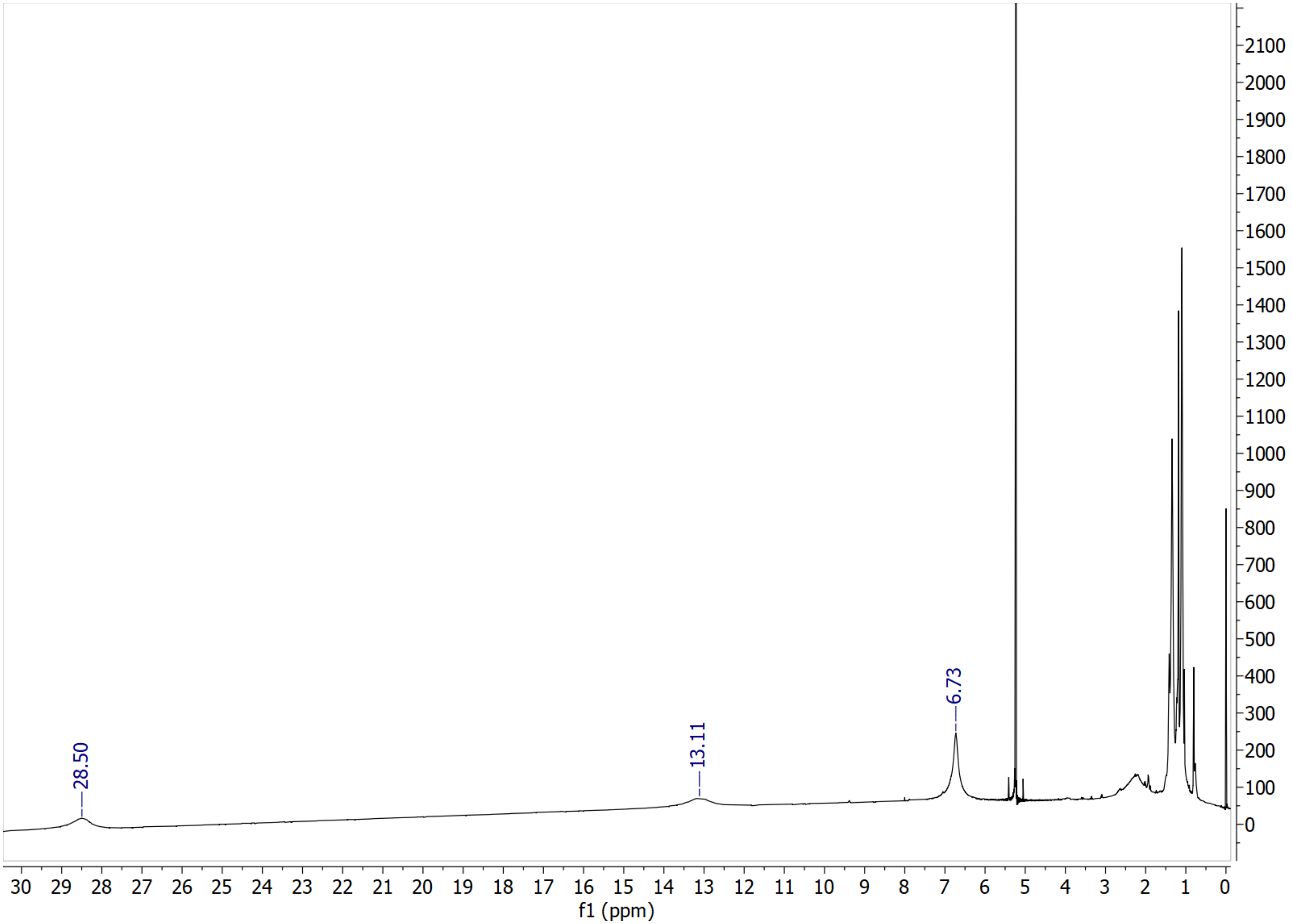

Figure S25: Full NMR spectrum of **6** taken in dry CD_2Cl_2 on a 500 MHz spectrometer

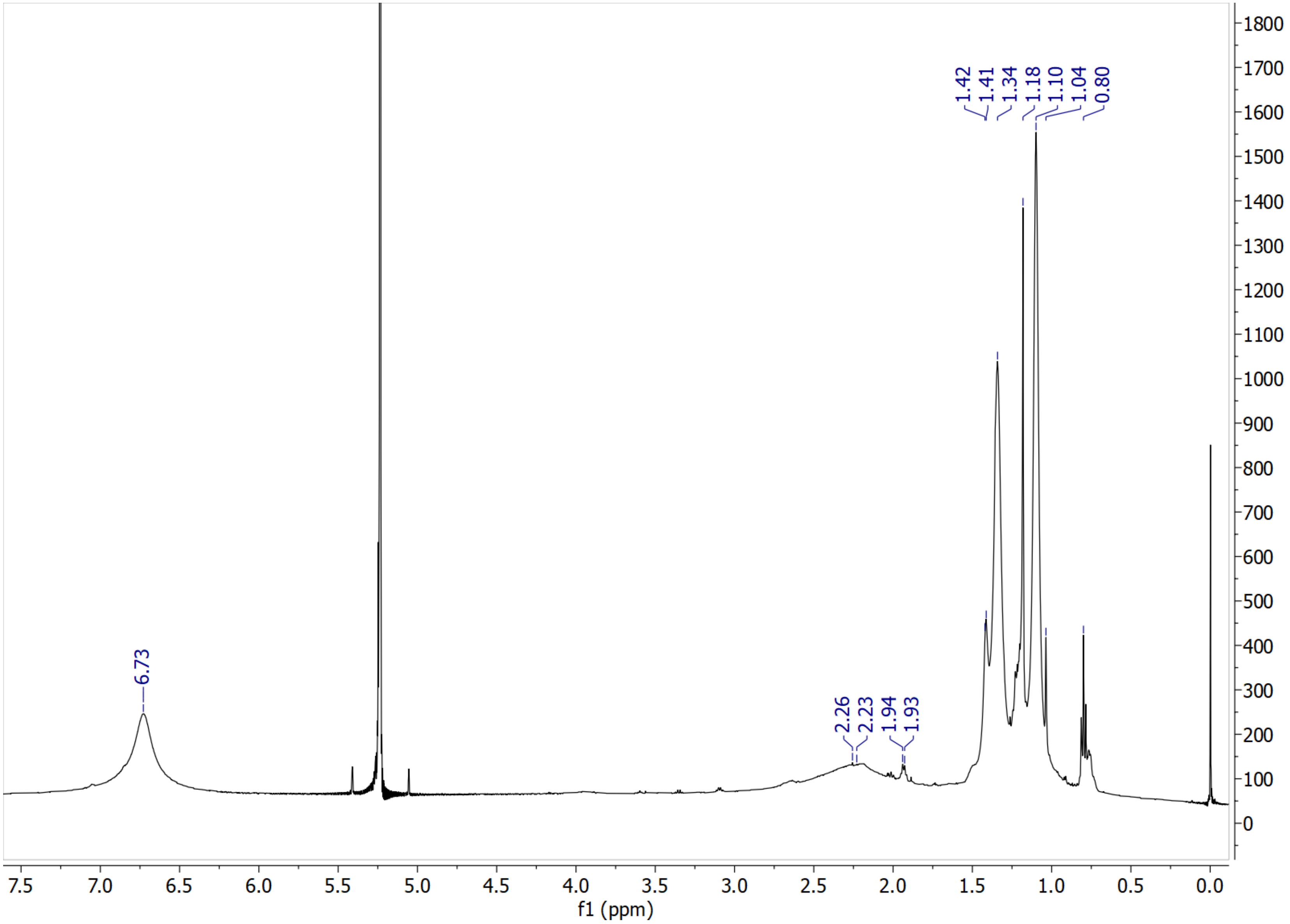

Figure S26: Zoomed in NMR spectrum of **6** taken in dry CD_2Cl_2 on a 500 MHz spectrometer

Collected Cyclic Voltammograms:

$Mo_2(dedpa)_4$ (**1**):

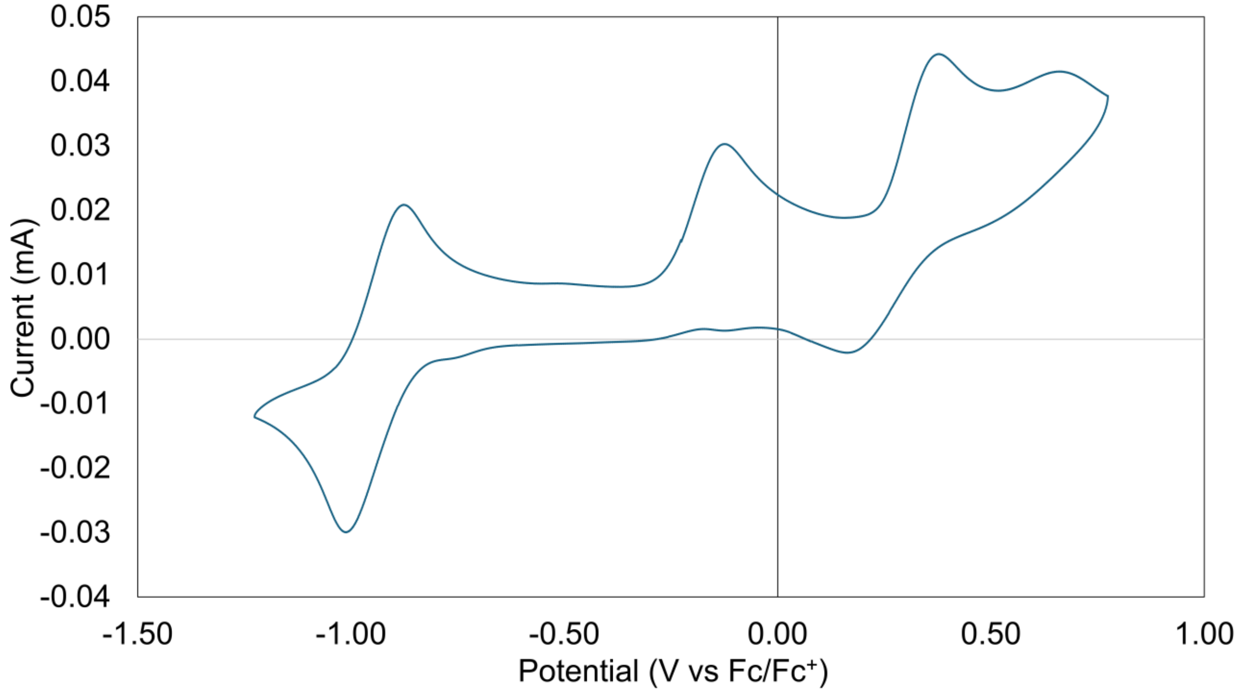

Figure S27: Cyclic voltammogram of 1 mM **1** in CH_2Cl_2 with 100 mM Bu_4NPF_6 as supporting electrolyte. Collected at 100 mV/s scan rate.

$Mo_2Cr(dedpa)_4Cl_2$ (**2**):

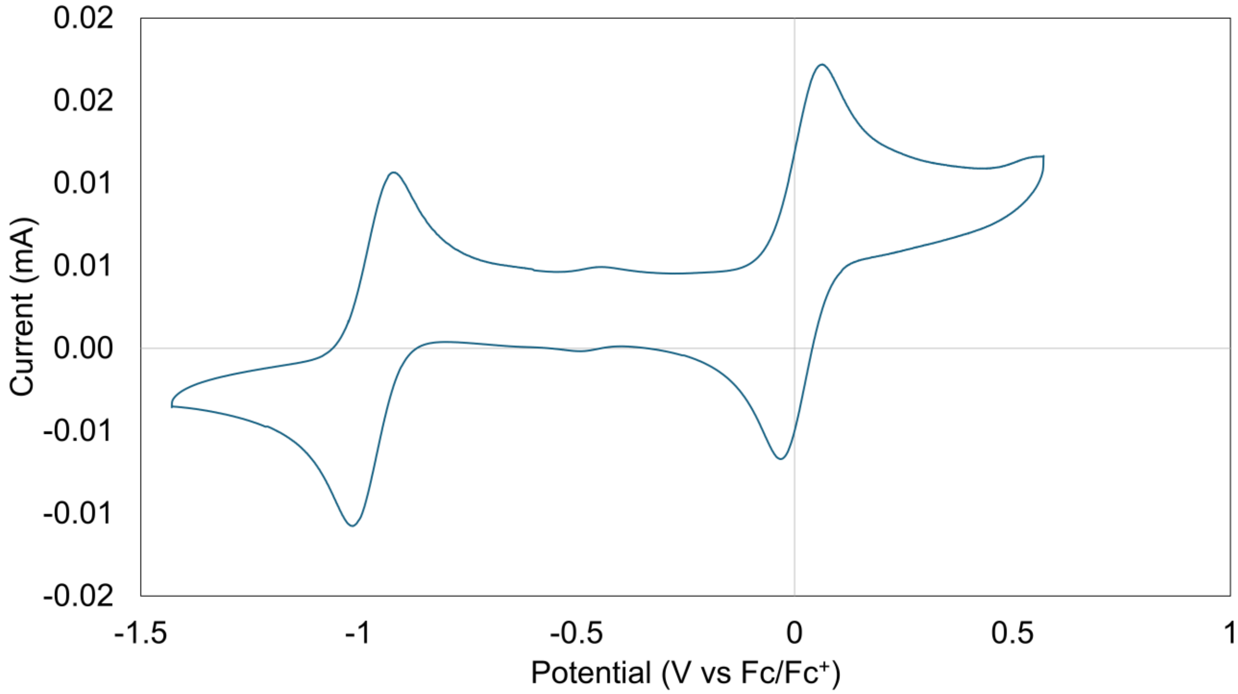

Figure S28: Cyclic voltammogram of 1 mM **2** in CH_2Cl_2 with 100 mM Bu_4NPF_6 as supporting electrolyte. Collected at 100 mV/s scan rate.

$Mo_2Mn(dedpa)_4Cl_2$ (**3**):

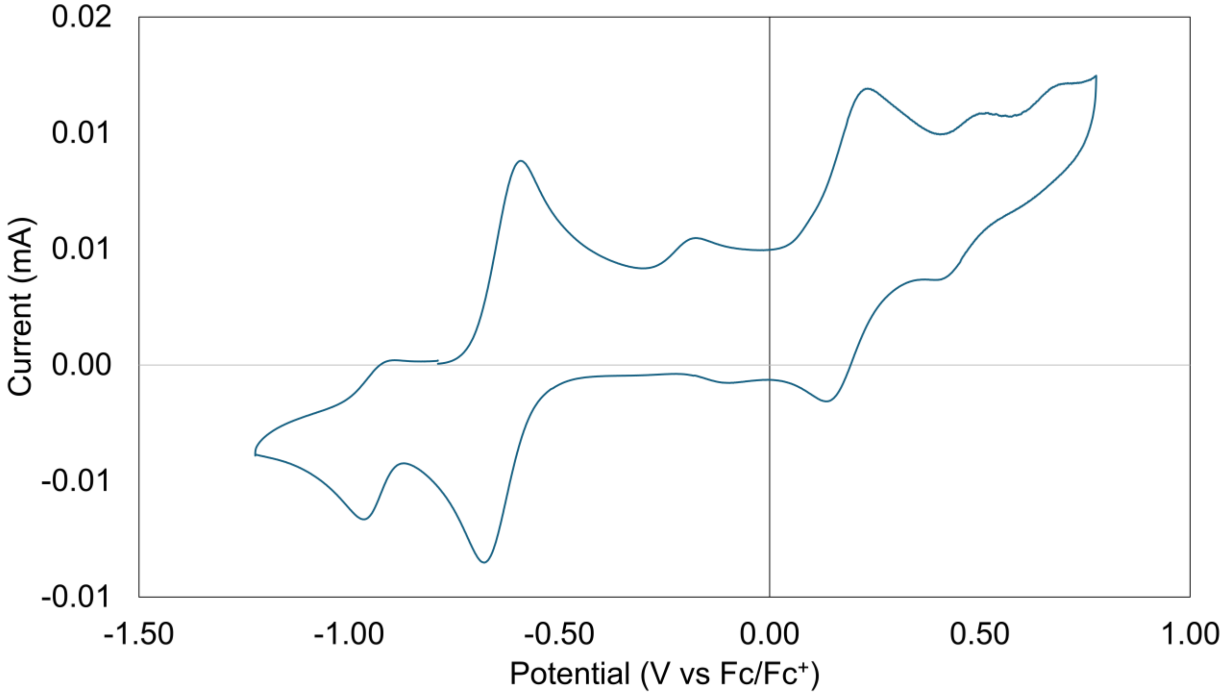

Figure S29: Cyclic voltammogram of 1 mM **3** in CH_2Cl_2 with 100 mM Bu_4NPF_6 as supporting electrolyte. Collected at 100 mV/s scan rate.

$Mo_2Fe(dedpa)_4Cl_2$ (**4**):

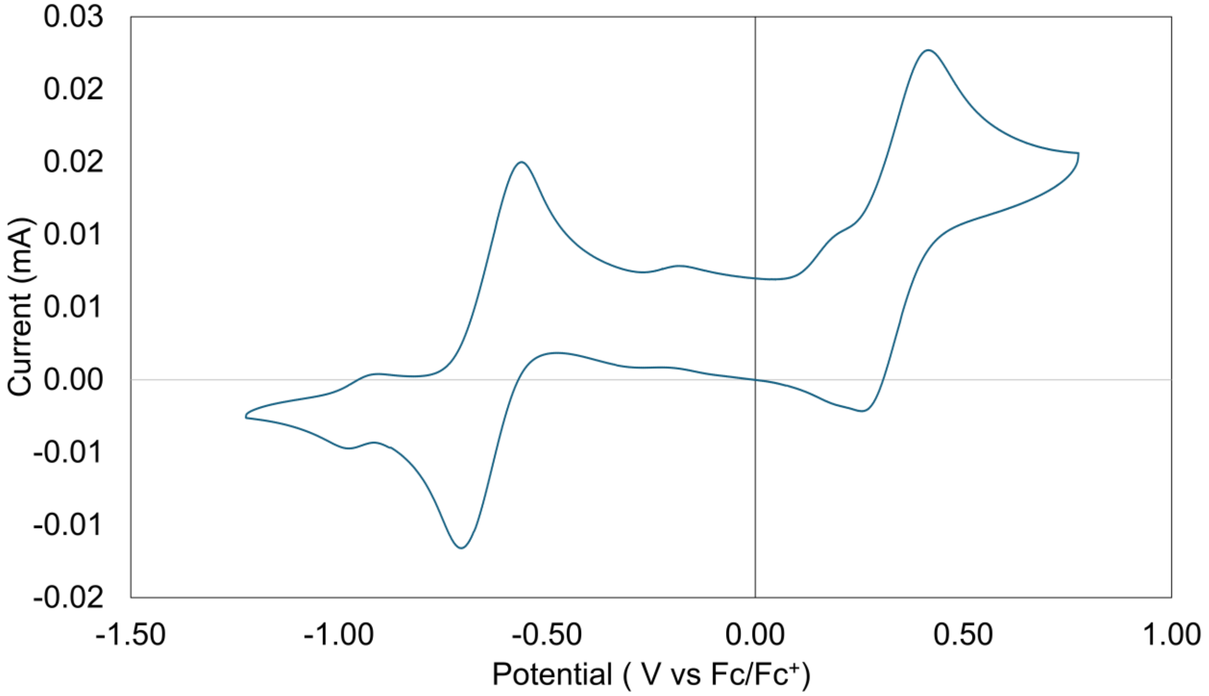

Figure S30: Cyclic voltammogram of 1 mM **4** in CH_2Cl_2 with 100 mM Bu_4NPF_6 as supporting electrolyte. Collected at 100 mV/s scan rate.

$Mo_2Co(dedpa)_4Cl_2$ (**5**):

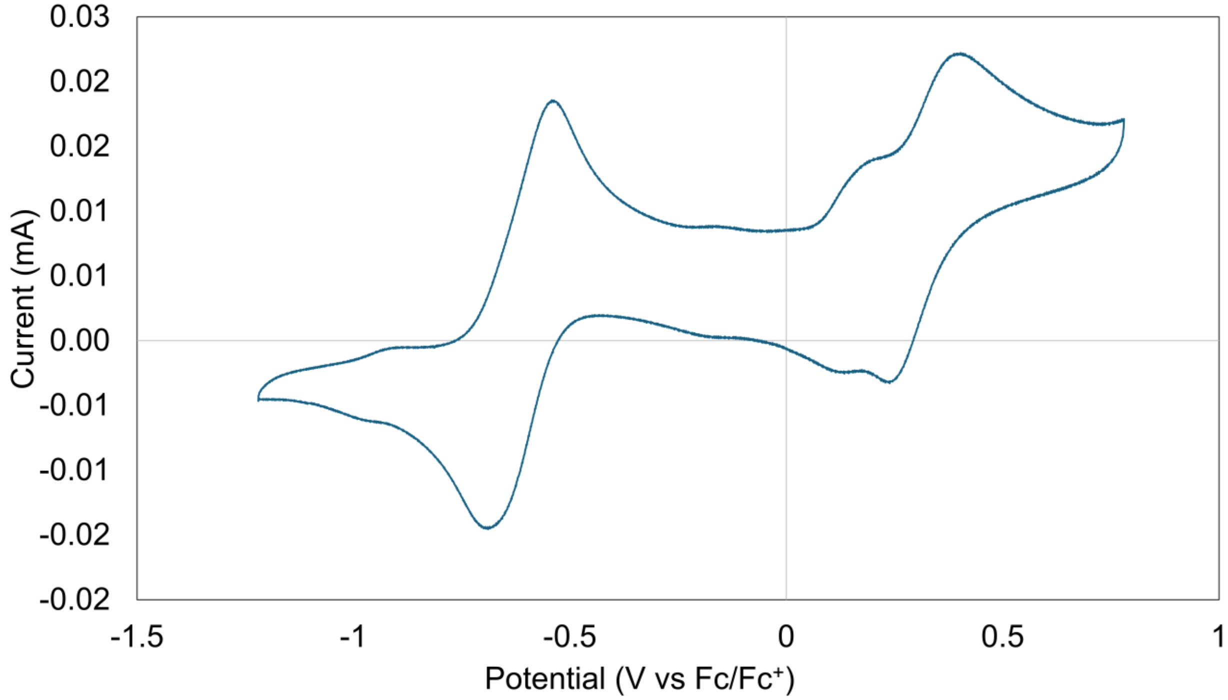

Figure S31: Cyclic voltammogram of 1 mM **5** in CH_2Cl_2 with 100 mM Bu_4NPF_6 as supporting electrolyte. Collected at 100 mV/s scan rate.

$Mo_2Ni(dedpa)_4Cl_2$ (**6**):

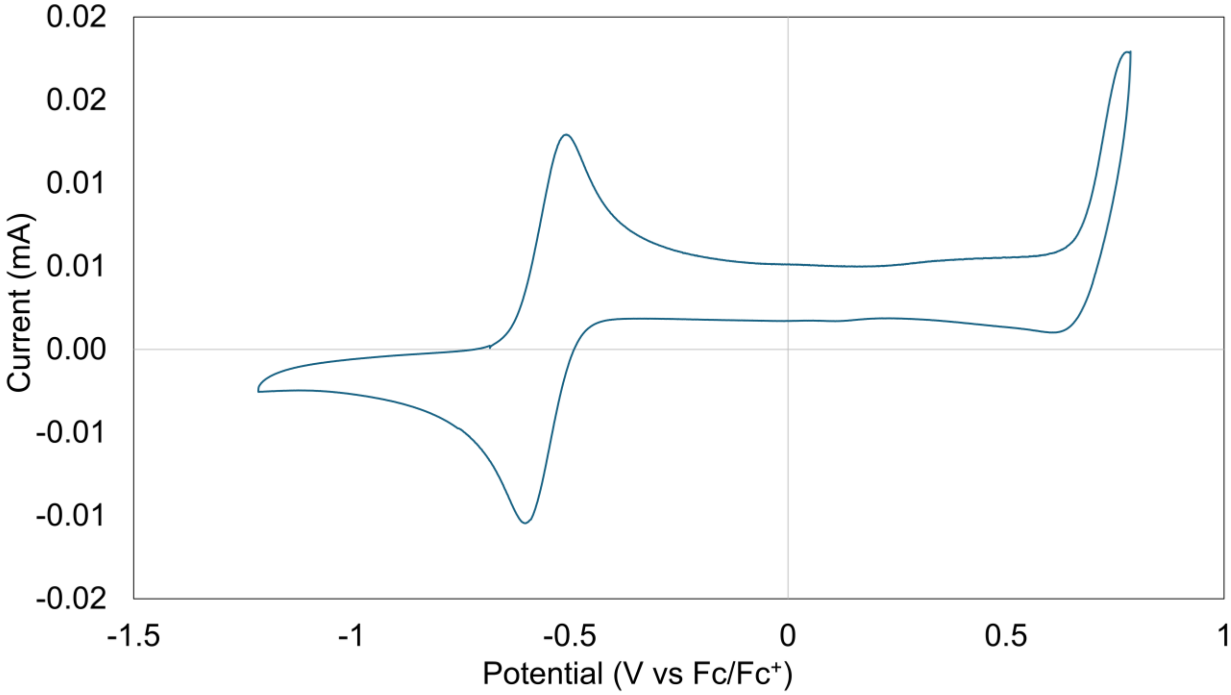

Figure S32: Cyclic voltammogram of 1 mM **6** in CH_2Cl_2 with 100 mM Bu_4NPF_6 as supporting electrolyte. Collected at 100 mV/s scan rate.

Table S1: EPR transitions visible in the spectrum of **3**, organized by g_{eff} values

Field (mT)	g_{eff}	Transition	Field direction
102	6.6	-1/2 to 1/2	*y*
106	6.3	-3/2 to 3/2	*z*
139	4.8	-1/2 to 1/2	*x*
152	4.4	1/2 to -3/2	*z*
226	3.0	3/2 to -5/2	*z*
300	2.2	-1/2 to -3/2	*z*
320	2.1	-1/2 to 1/2	*z*
354	1.9	-3/2 to 3/2	*x*
385	1.7	-5/2 to 3/2	*z*
420	1.6	-3/2 to 3/2	*y*
471	1.4	-3/2 to 1/2	*z*
510	1.3	1/2 to -5/2	*z*
721	0.93	-5/2 to 1/2	*z*
760	0.88	-1/2 to -5/2	*z*

Collected Magnetometry Data for **2-6**:

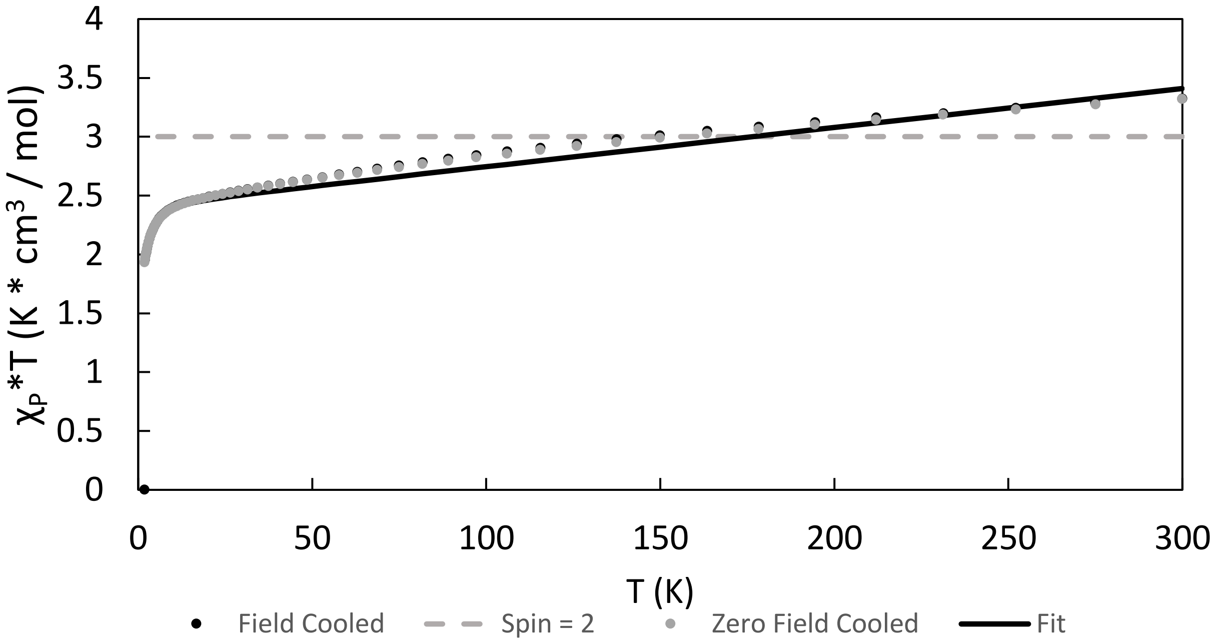

Figure S33: Overlay of field cooled and zero field cooled magnetic susceptibility data for **2** with fit

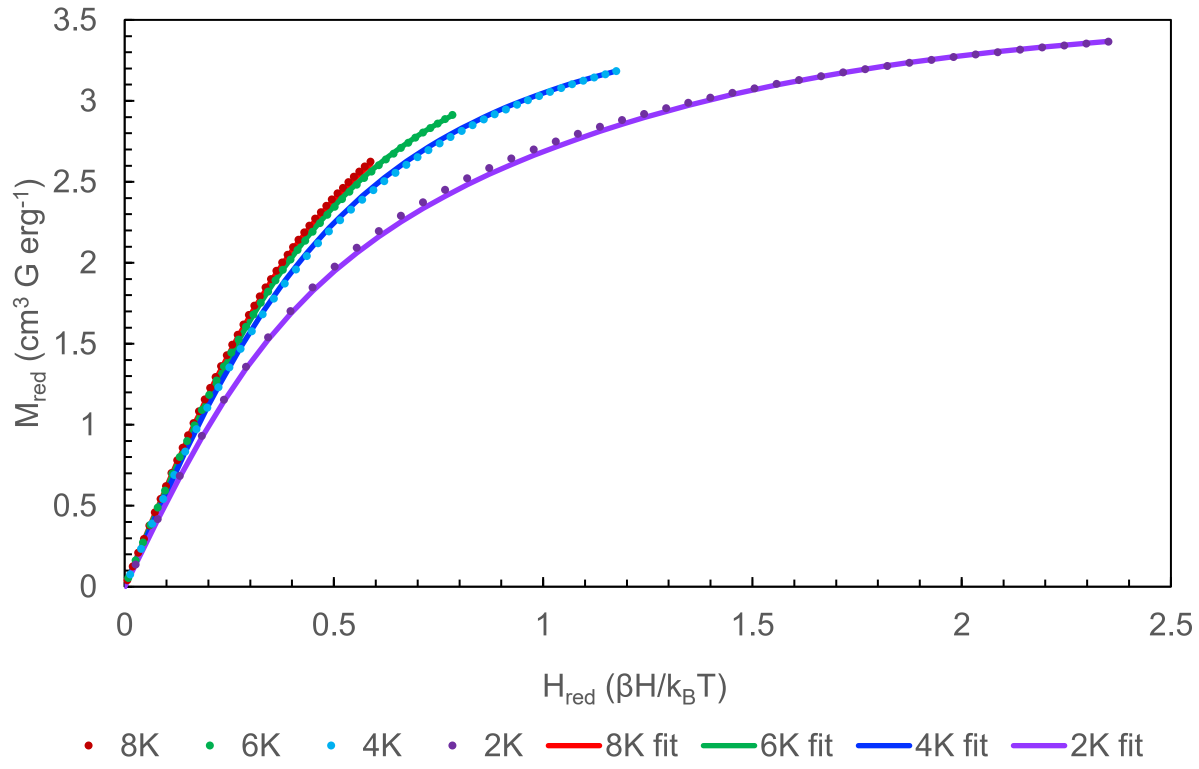

Figure S34: Reduced magnetization data of **2** at 2K, 4K, 6K, and 8K with fits for each.

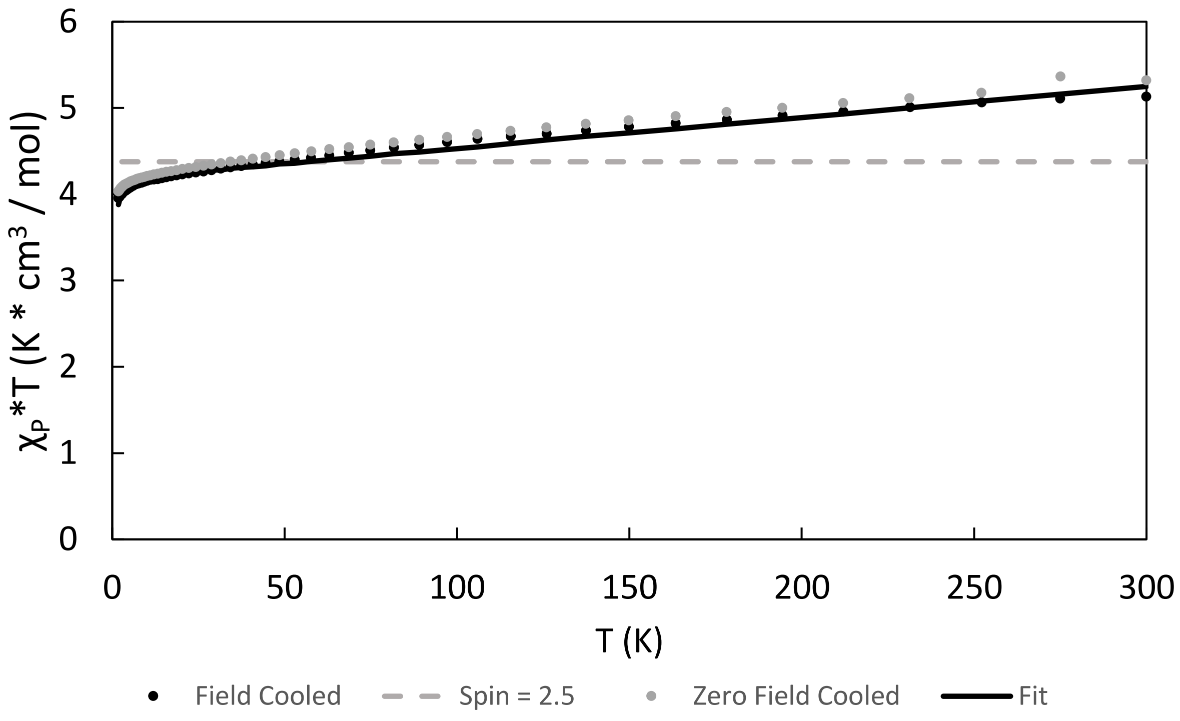

Figure S35: Overlay of field cooled and zero field cooled magnetic susceptibility data for **3** with fit

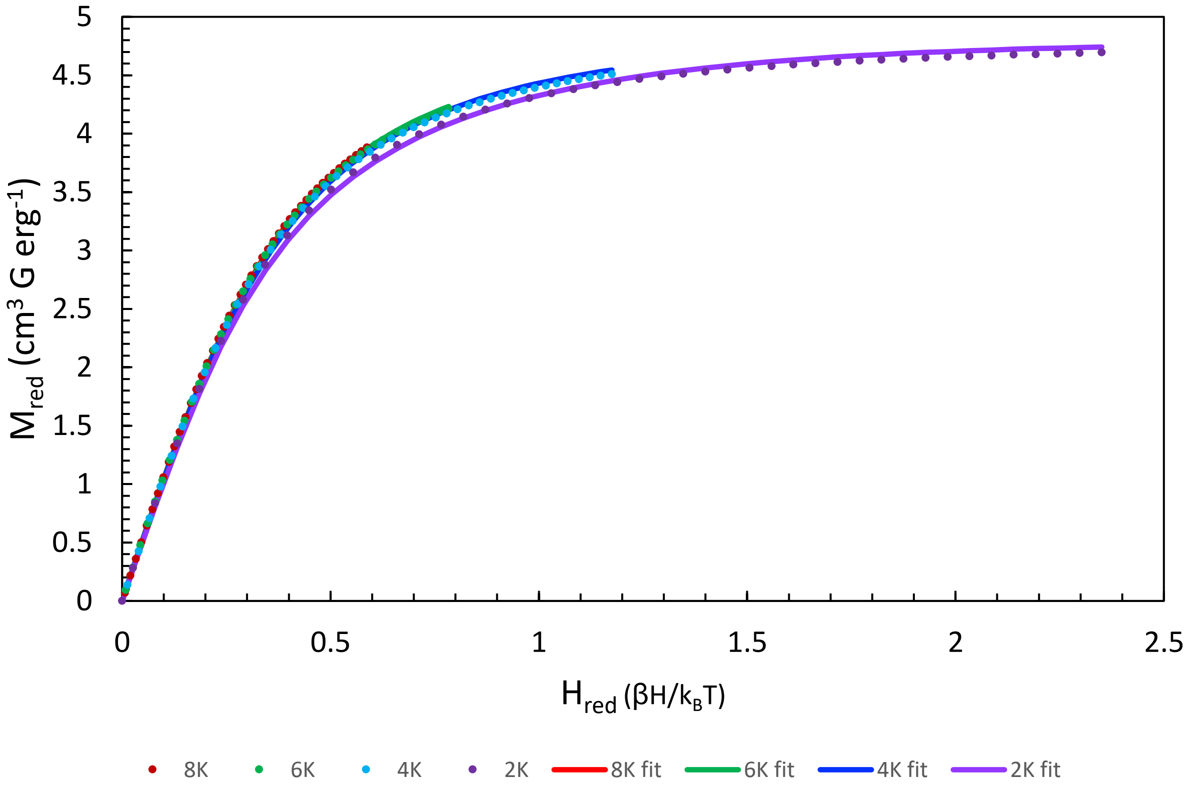

Figure S36: Reduced magnetization data of **3** at 2K, 4K, 6K, and 8K with fits for each.

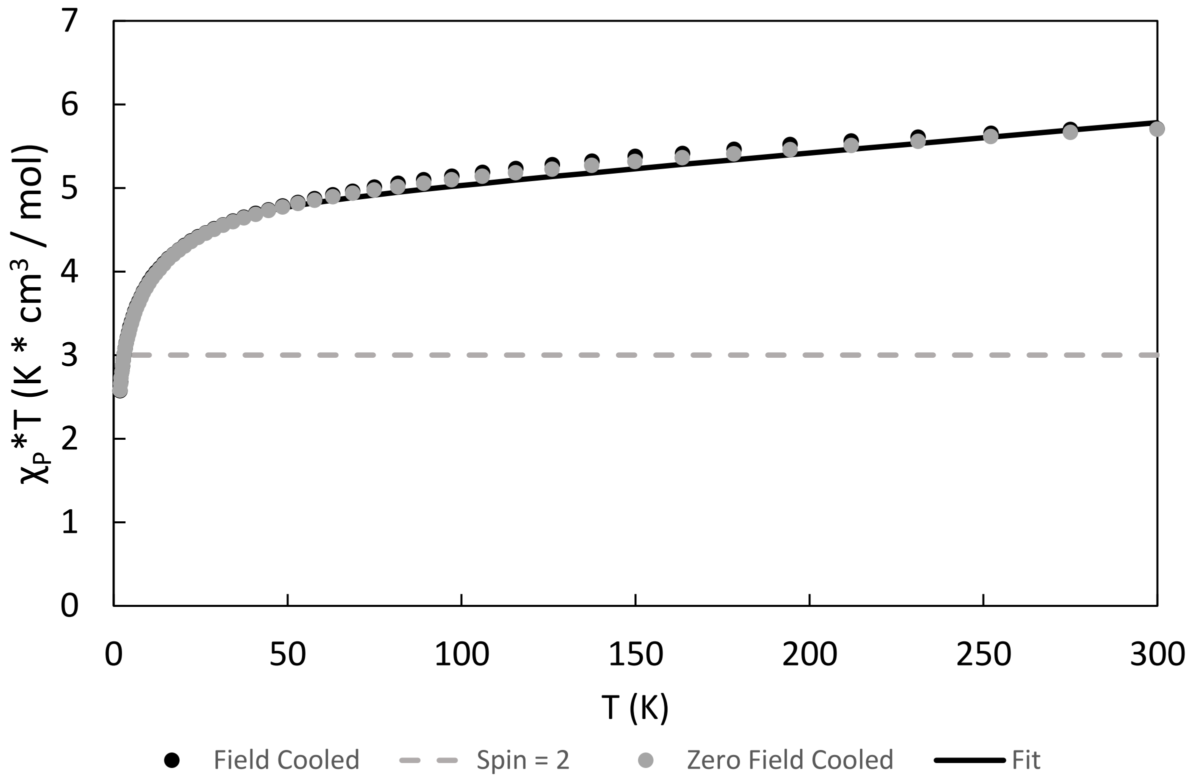

Figure S37: Overlay of field cooled and zero field cooled magnetic susceptibility data for **4** with fit

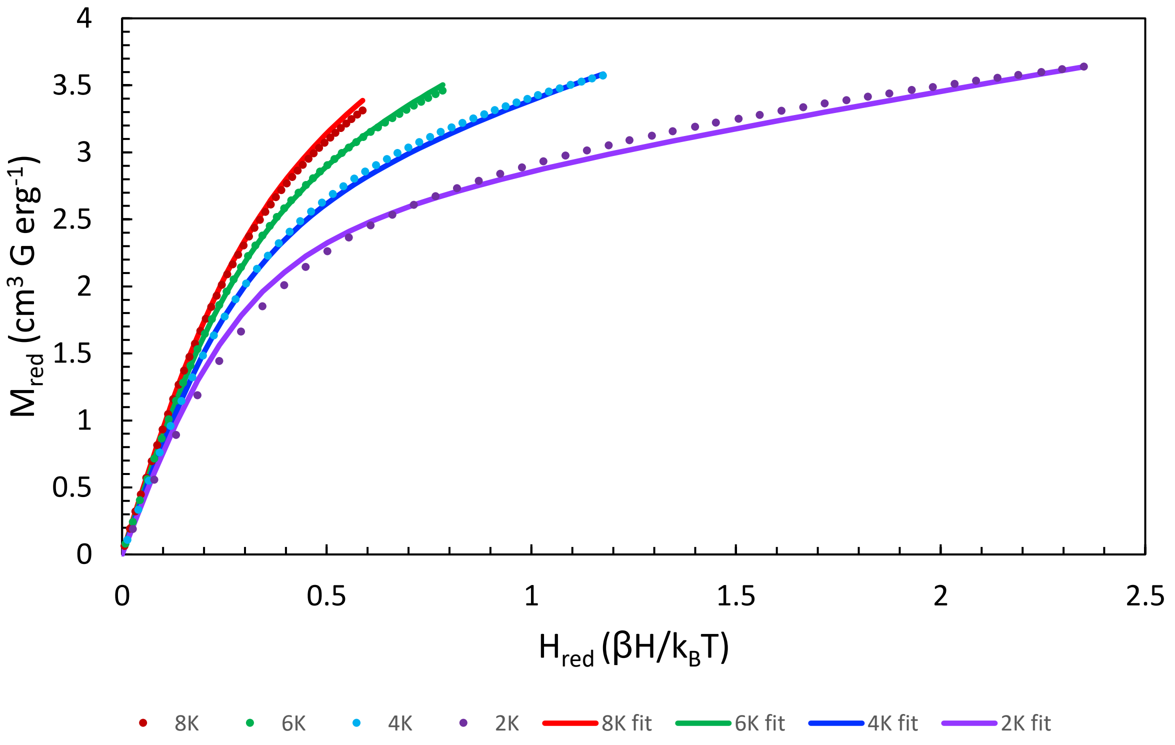

Figure S38: Reduced magnetization data of **4** at 2K, 4K, 6K, and 8K with fits for each.

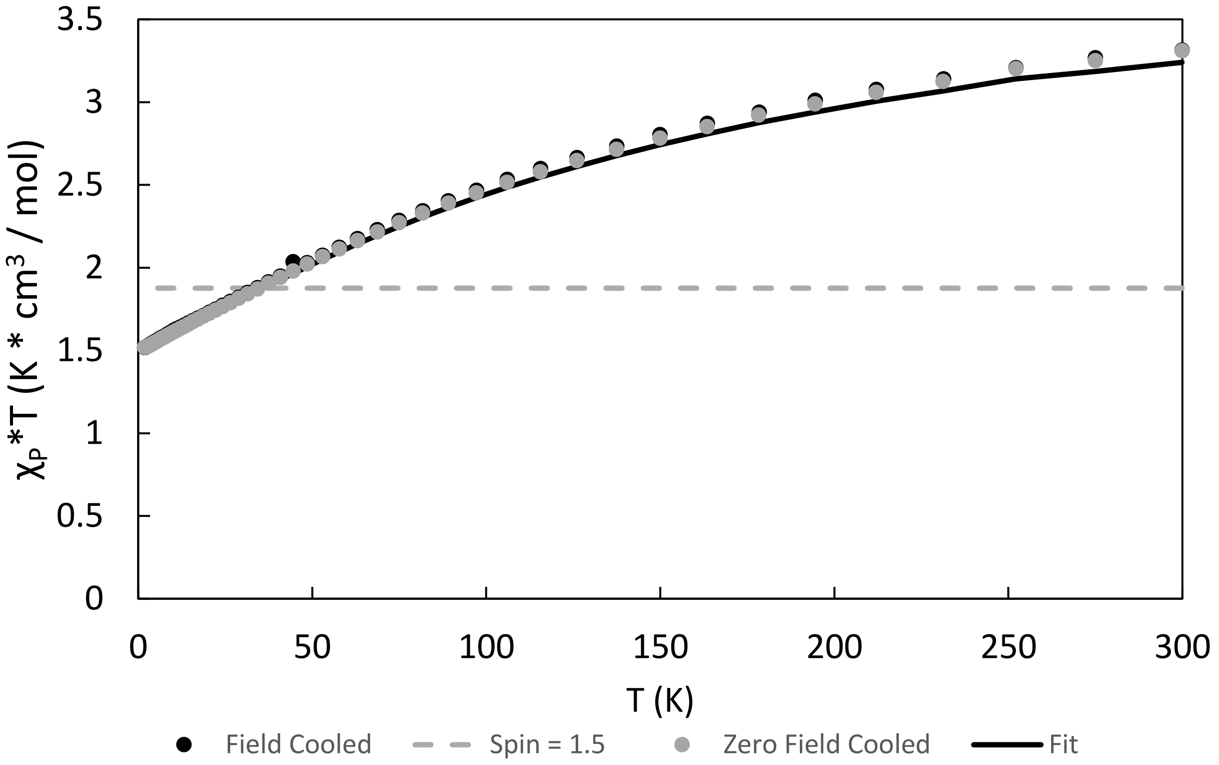

Figure S39: Overlay of field cooled and zero field cooled magnetic susceptibility data for **5** with fit

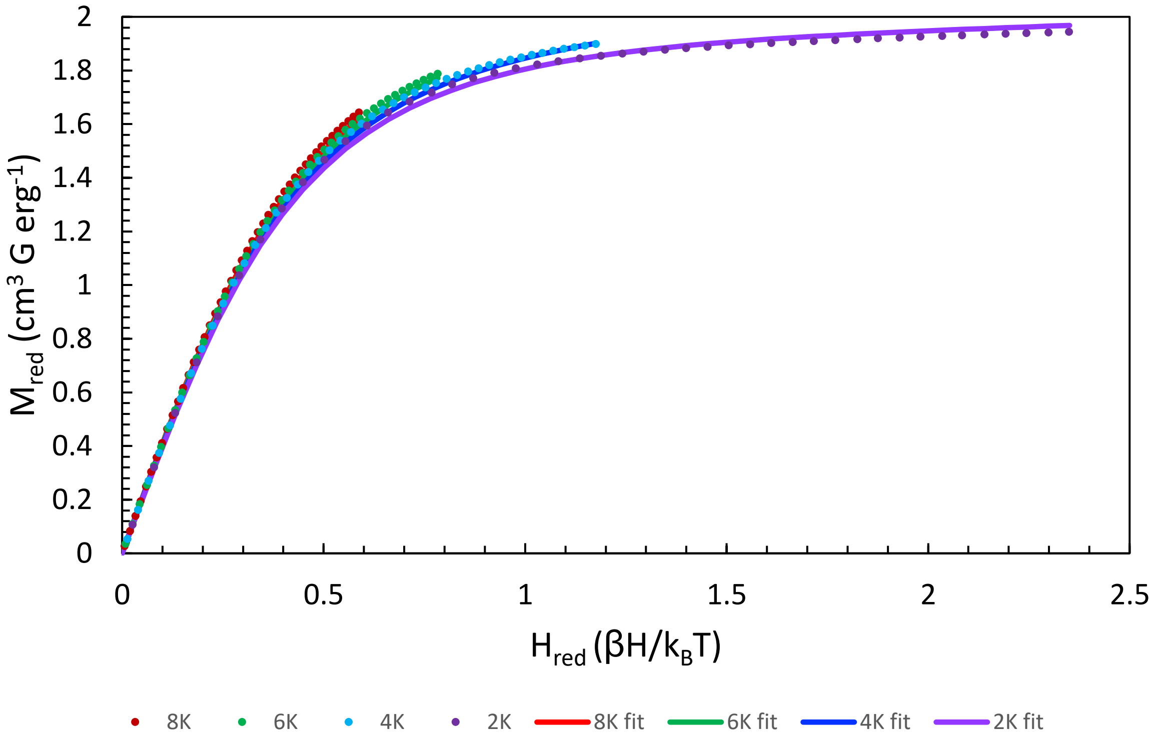

Figure S40: Reduced magnetization data of **5** at 2K, 4K, 6K, and 8K with fits for each.

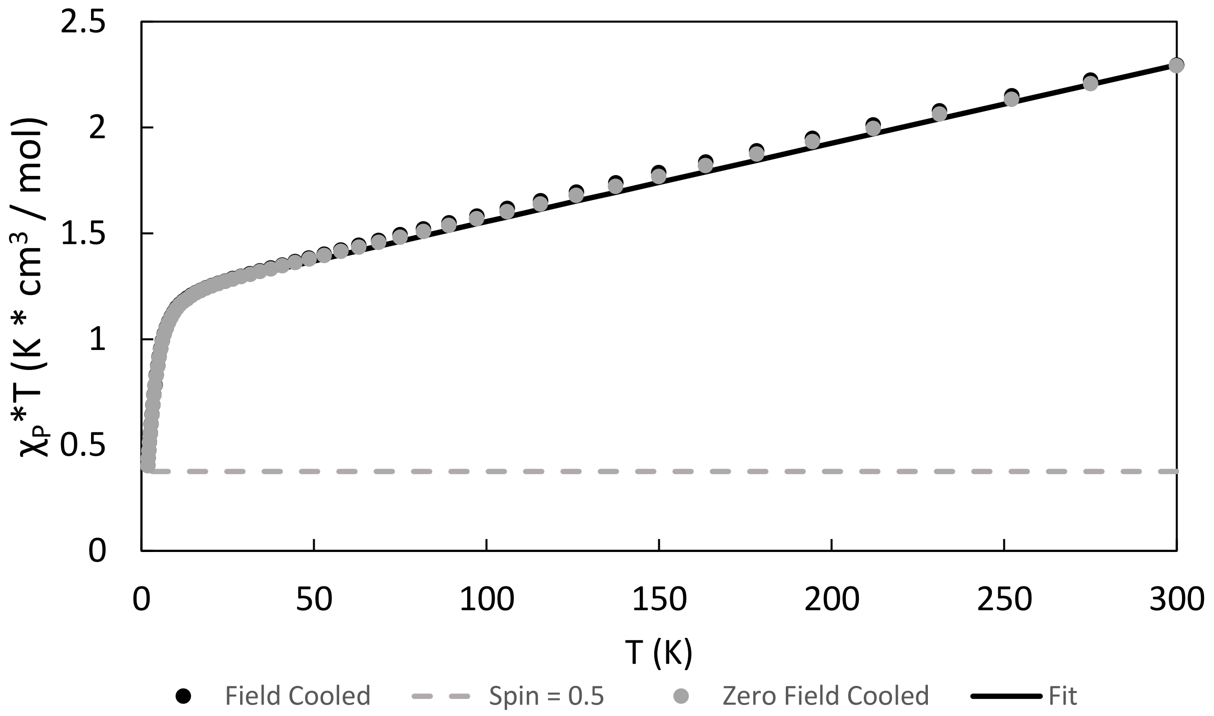

Figure S41: Overlay of field cooled and zero field cooled magnetic susceptibility data for **6** with fit

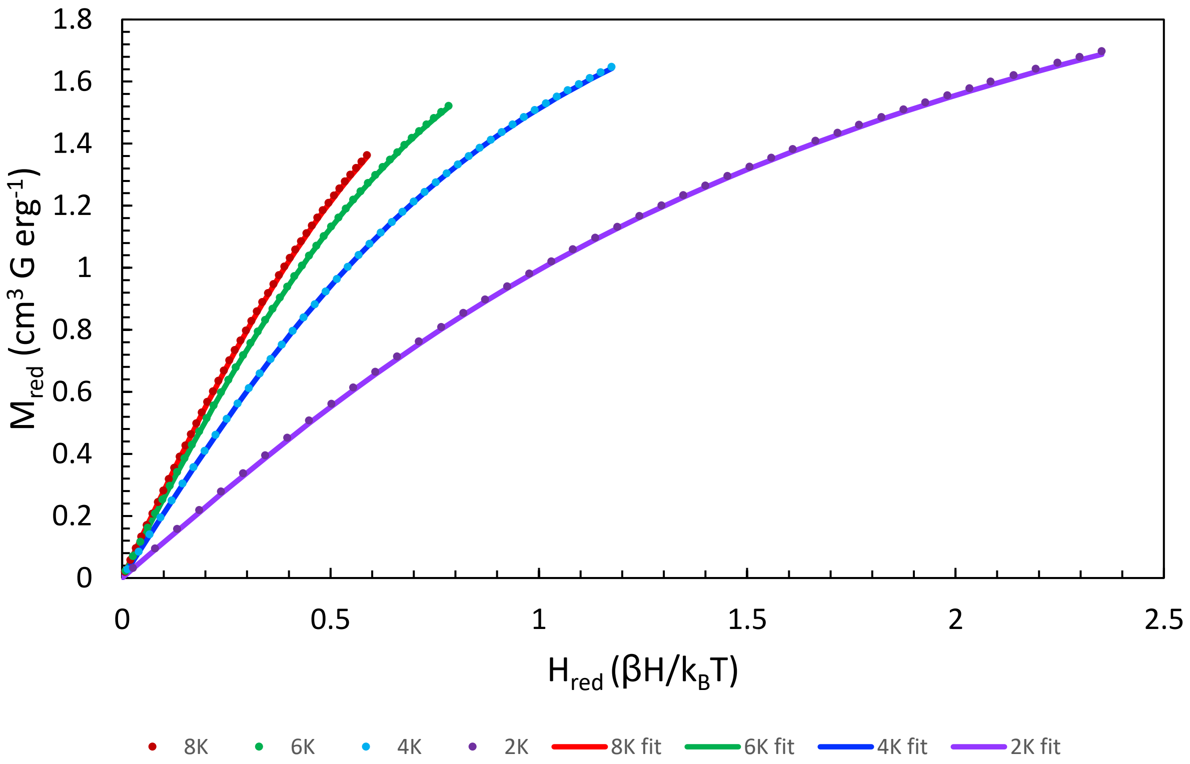

Figure S42: Reduced Magnetization data of **6** at 2K, 4K, 6K, and 8K with fits for each.

Crystallographic Details:

Table S2: Crystal Data for **1-6:**

	**1**	**2**	**3**	**4**	**5**	**6**
Empirical formula	$C_{56}H_{64}Mo_2N_{12}$	$C_{56}H_{64}Cl_2CrMo_2N_{12}$	$C_{58}H_{68}Cl_6MnMo_2N_{12}$	$Mo_2FeC_{56}H_{64}N_{12}Cl_2$	$Mo_2CoC_{56}H_{64}N_{12}Cl_2$	$C_{56}H_{64}Cl_2Mo_2N_{12}Ni$
Crystal system	Tetragonal	Tetragonal	Cubic	Tetragonal	Tetragonal	Tetragonal
Space group	$I4_1/a$	*P*4*nc*	*P*432	*P*4*nc*	*P*4*nc*	*P*4*nc*
a/Å	21.6108(4)	11.7107(5)	21.7029(10)	11.7802(4)	11.729(2)	11.7384(5)
b/Å	21.6108(4)	11.7107(5)	21.7029(10)	11.7802(4)	11.729(2)	11.7384(5)
c/Å	10.6990(3)	19.0748(17)	21.7029(10)	19.0119(10)	18.998(6)	18.9755(12)
α/°	90	90	90	90	90	90
β/°	90	90	90	90	90	90
γ/°	90	90	90	90	90	90
Volume/Å^3	4996.7(2)	2615.9(3)	10222.4(14)	2638.3(2)	2613.4(13)	2614.6(3)
Z	4	2	6	2	2	2
ρ_{calc} g/cm^3	1.458	1.549	1.357	1.541	1.559	1.558
$R_1{}^a$, $wR_2{}^b$ [I>=2σ (I)]	0.0208, 0.0537	0.0547, 0.1839	0.0368, 0.1053	0.0471, 0.1419	0.0556, 0.1676	0.0455, 0.1212
$R_1{}^a$, $wR_2{}^b$ [all data]	0.0216, 0.0542	0.0596, 0.1961	0.0393, 0.1073	0.0699, 0.1646	0.0726, 0.1900	0.0556, 0.1315

[a] $R_1 = \sum||F_o| - |F_c||/\sum|F_o|$. [b] $wR_2 = [\sum[w(F_o^2 - F_c^2)^2]/\sum[w(F_o^2)^2]]^{1/2}$, $w = 1/\sigma^2(F_o^2) + (aP)^2 + bP$, where $P = [\max(0 \text{ or } F_o^2) + 2(F_c^2)]/3$

Table S3: Selected Bond Lengths for $Mo_2M'dpa_4Cl_2$ compounds**:**

M'	Mo–Mo (Å)	Mo–M (Å)	M–N (Å)	Mo_i–N (Å)	Mo_o–N (Å)	Mo–L_{ax} (Å)	M–L_{ax} (Å)	Ref.
**Cr** · 2 CH_2Cl_2	2.0984(4)	2.6885(6)	2.127[2]	2.127[2]	2.217[2]	2.7891(8)	2.535(1)	[41]
**Mn**[a]	2.094(3)	2.793(4)	2.249[2]	2.136[3]	2.210[9]	2.7684(13)	2.327(3)	[1]
**Fe** · 2 CH_2Cl_2	2.104(1)	2.762(1)	2.186[2]	2.129[2]	2.214[2]	2.747(1)	2.337(1)	[1]
**Co** · 1.5 CH_2Cl_2	2.1027(5)	2.6170(7)	2.1025[11]	2.1205[4]	2.2155[9]	2.7200(9)	2.430(1)	[42]
**Ni** · C_4H_8O	2.107(2)	2.525(4)	2.146[3]	2.115[3]	2.174[3]	2.627(2)	2.394(3)	[43]

[a]in instances of metal-atom chain disorder, the majority position was used for geometric parameters.

Additional details and molecular drawings for **1-6**:

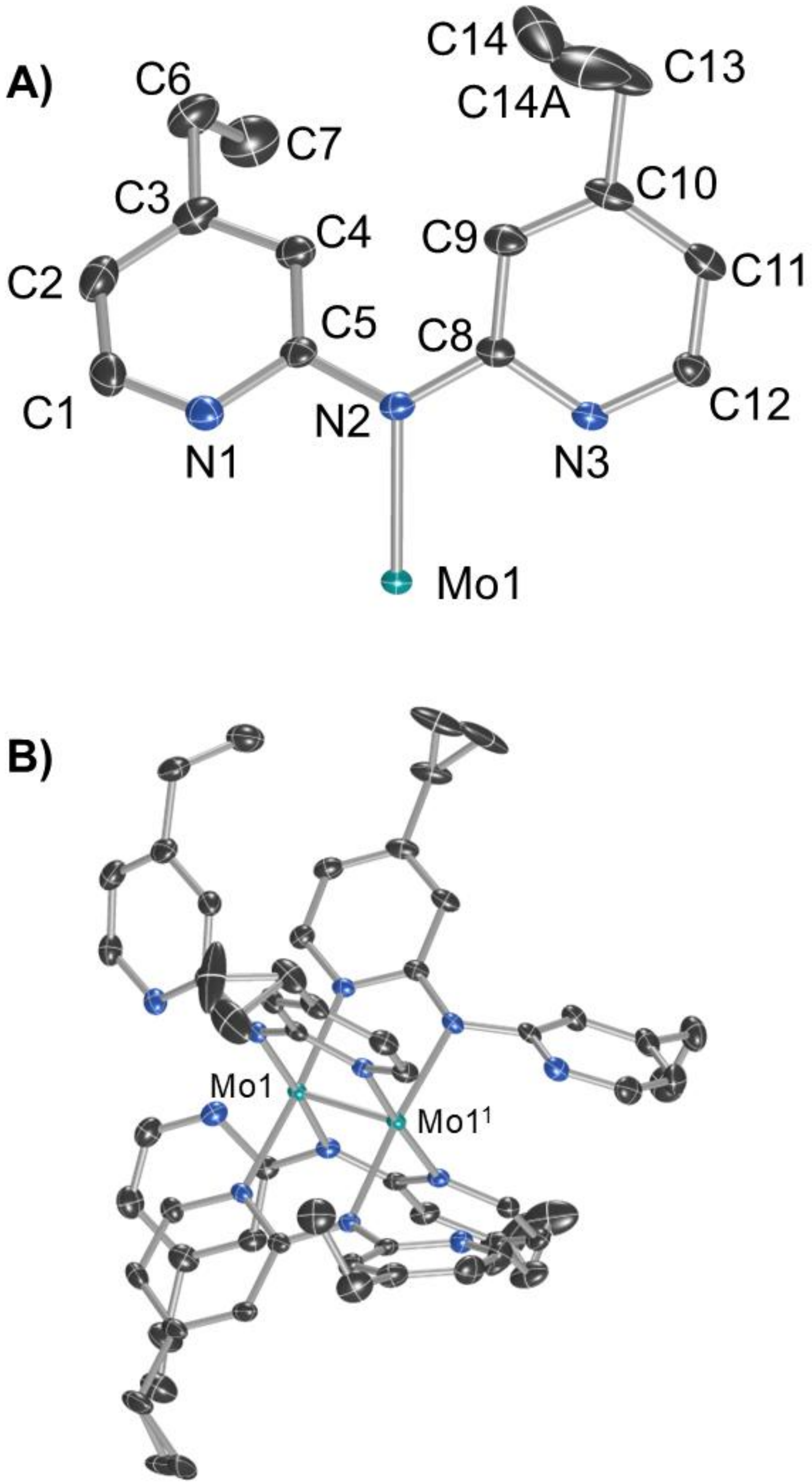

Figure S43: Part A shows the asymmetric unit of **1**, showing ¼ of the molecule. Ethyl group carbon C14 is split over two positions (C14 and C14A) with an occupancy ratio of 59.9:41.1. Part B shows the full structure of **1**. For all drawings, hydrogens are omitted for clarity and ellipsoids are drawn at 50% probability.

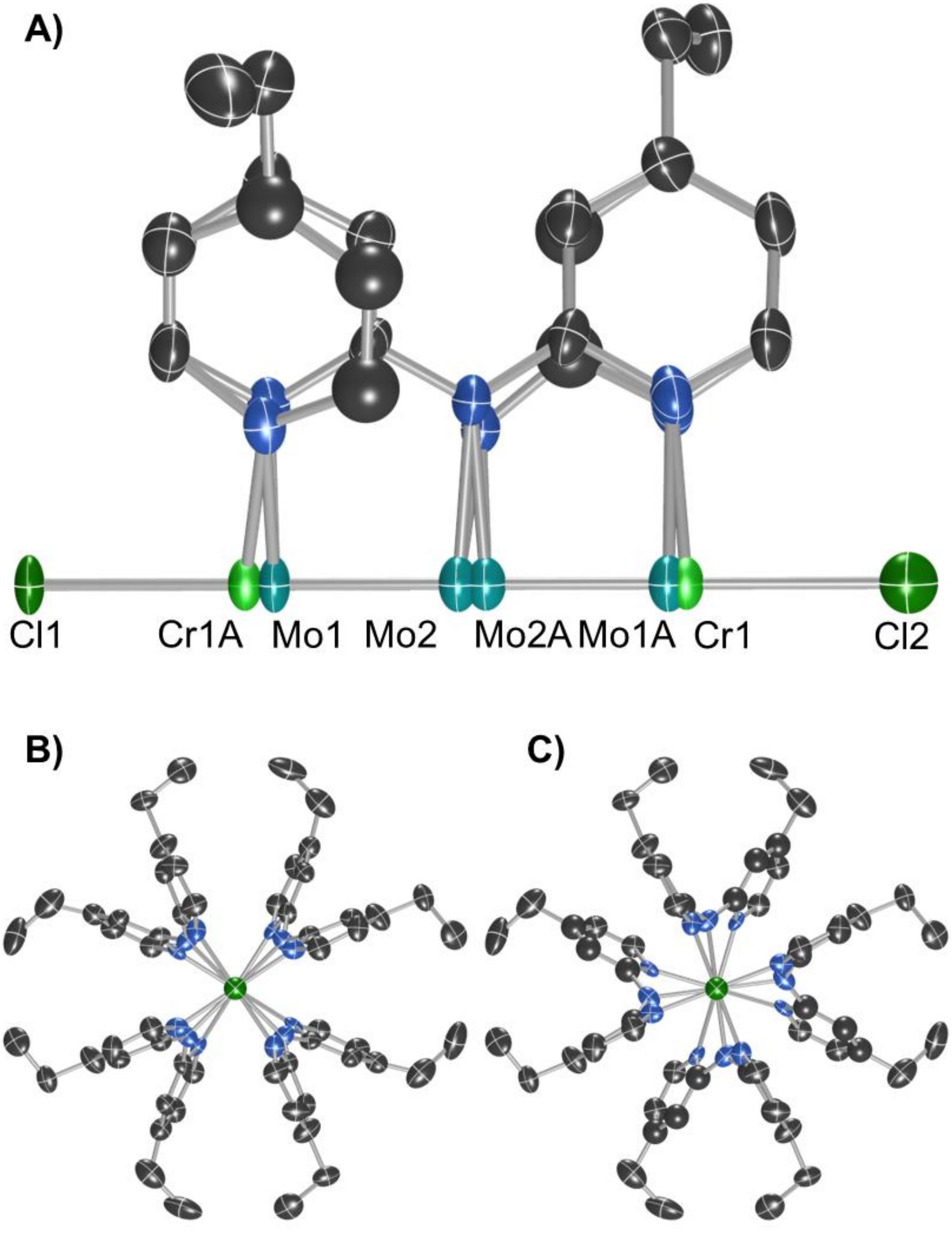

Figure S44: Part A shows the asymmetric unit of **2**, which exhibits disorder in both the metal atom positions and ligand positions. The metal atom components are split almost 50:50, with the major component having an occupancy of 52.2(15)%. B shows a view along Cl1 of the major ligand component (rho isomer, 75.3(6)%) while C shows a view along Cl1 of the minor ligand component (delta isomer, 24.7%). For all structures, the ellipsoids are shown at 50% probability and hydrogens are omitted for clarity.

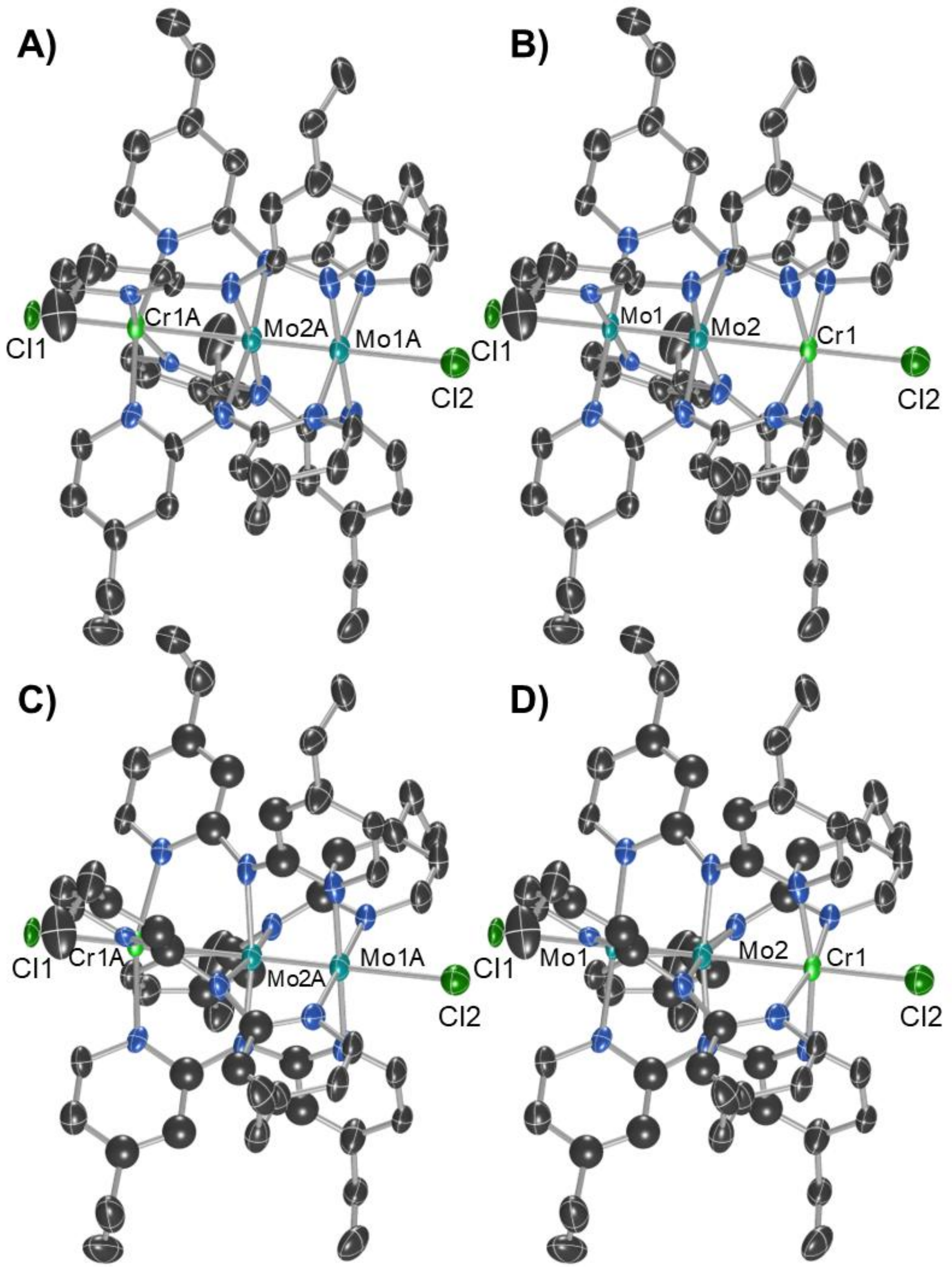

Figure S45: The four possible combinations of ligand and metal orientation for **2** are shown in A-D, with the major ligand component shown in A and B and the major metal component shown in B and D. Ellipsoids are drawn at 50% probability and hydrogens are omitted for clarity.

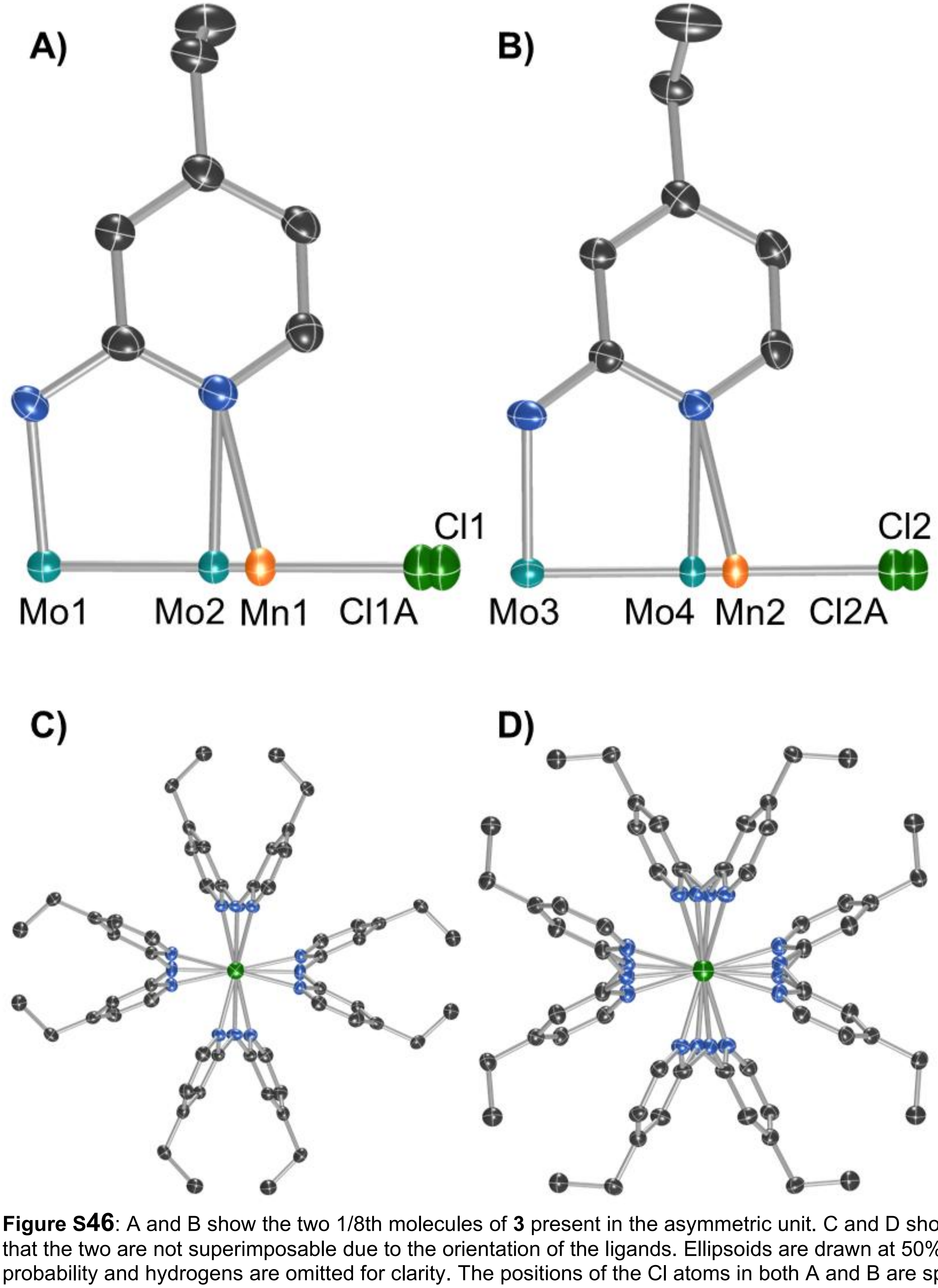

Figure S46: A and B show the two 1/8th molecules of **3** present in the asymmetric unit. C and D show that the two are not superimposable due to the orientation of the ligands. Ellipsoids are drawn at 50% probability and hydrogens are omitted for clarity. The positions of the Cl atoms in both A and B are split over two sites in a 50:50 ratio (Cl1/Cl1A and Cl2/Cl2A).

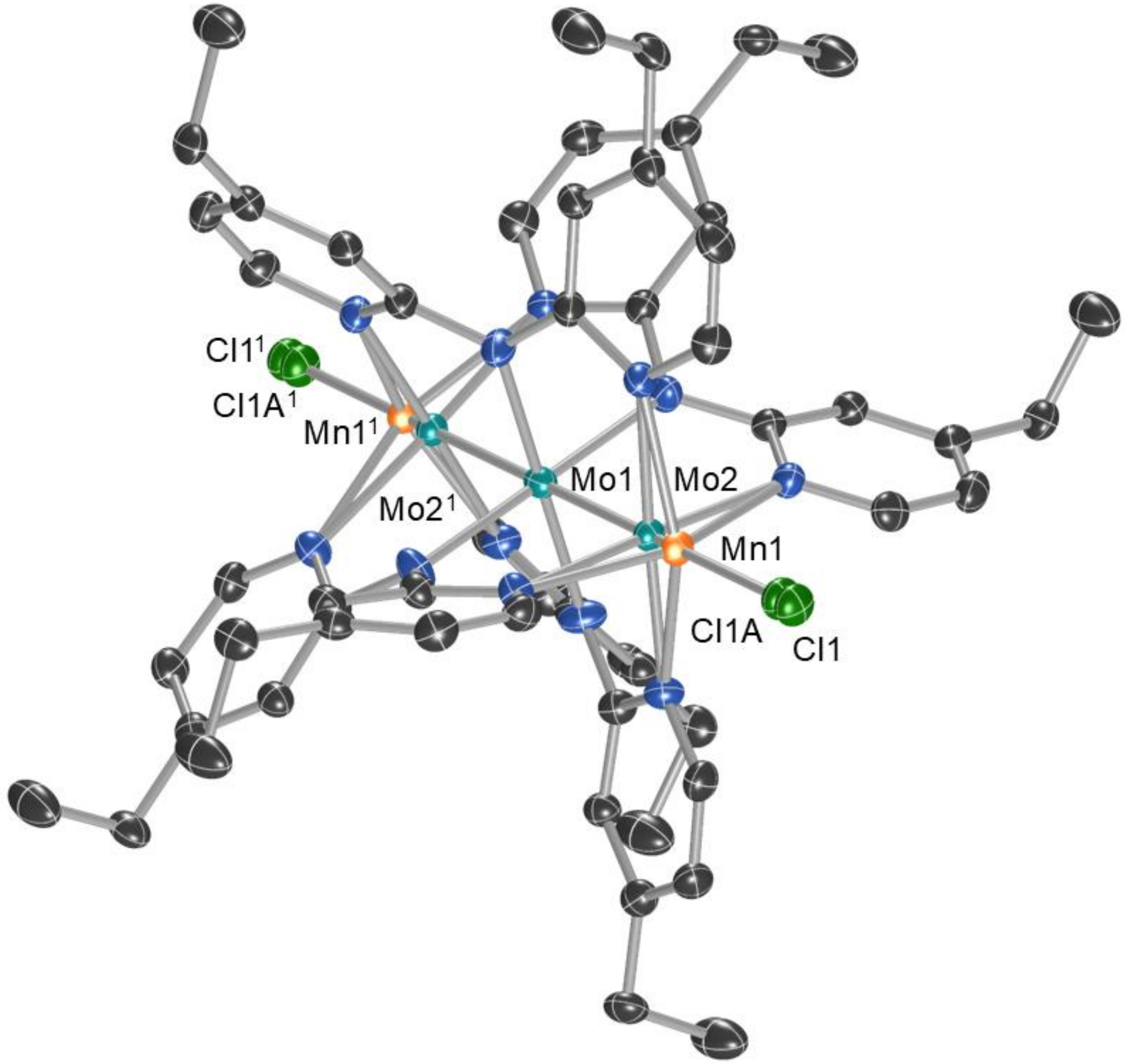

Figure S47: The grown structure of one of the molecules of **3** present in the unit cell. The Mn and Mo2 atoms are split 50:50 over two positions in the structure. The Cl atoms are also split over two positions with 50% occupancy in both. Ellipsoids are drawn at 50% probability and hydrogen atoms are omitted for clarity.

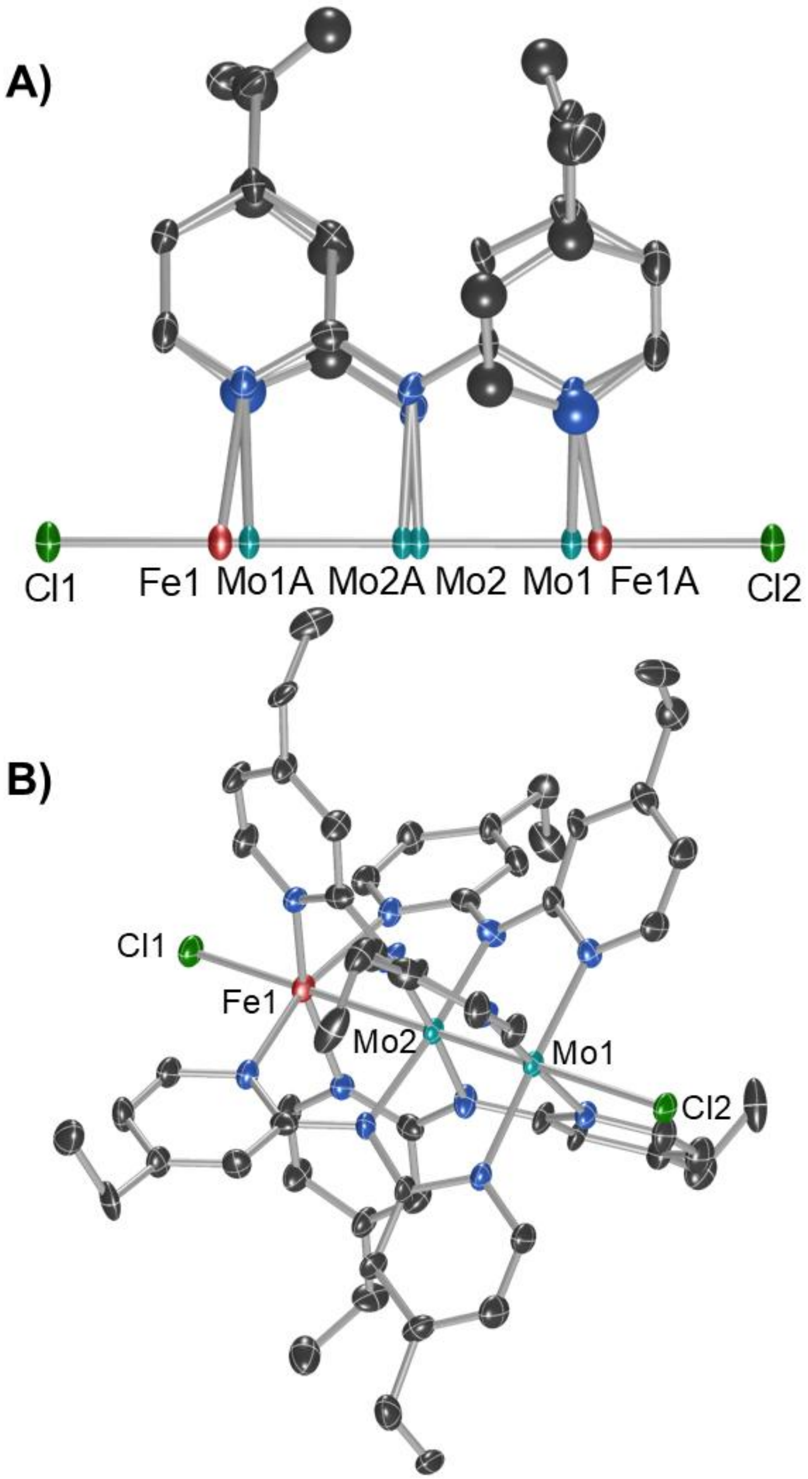

Figure S48: A shows the asymmetric unit of **4**, showing disorder in both the metal atom positions and ligand positions. The metal atom components are split almost 50:50, with the major component having an occupancy of 52.2(11)% while the ligands have a preference for the rho isomer with the major component having an occupancy of 84.6(4)%. B shows a drawing of the full structure of **4** with only the major components of the structure shown. Ellipsoids are drawn at 50% probability and hydrogen atoms are omitted for clarity.

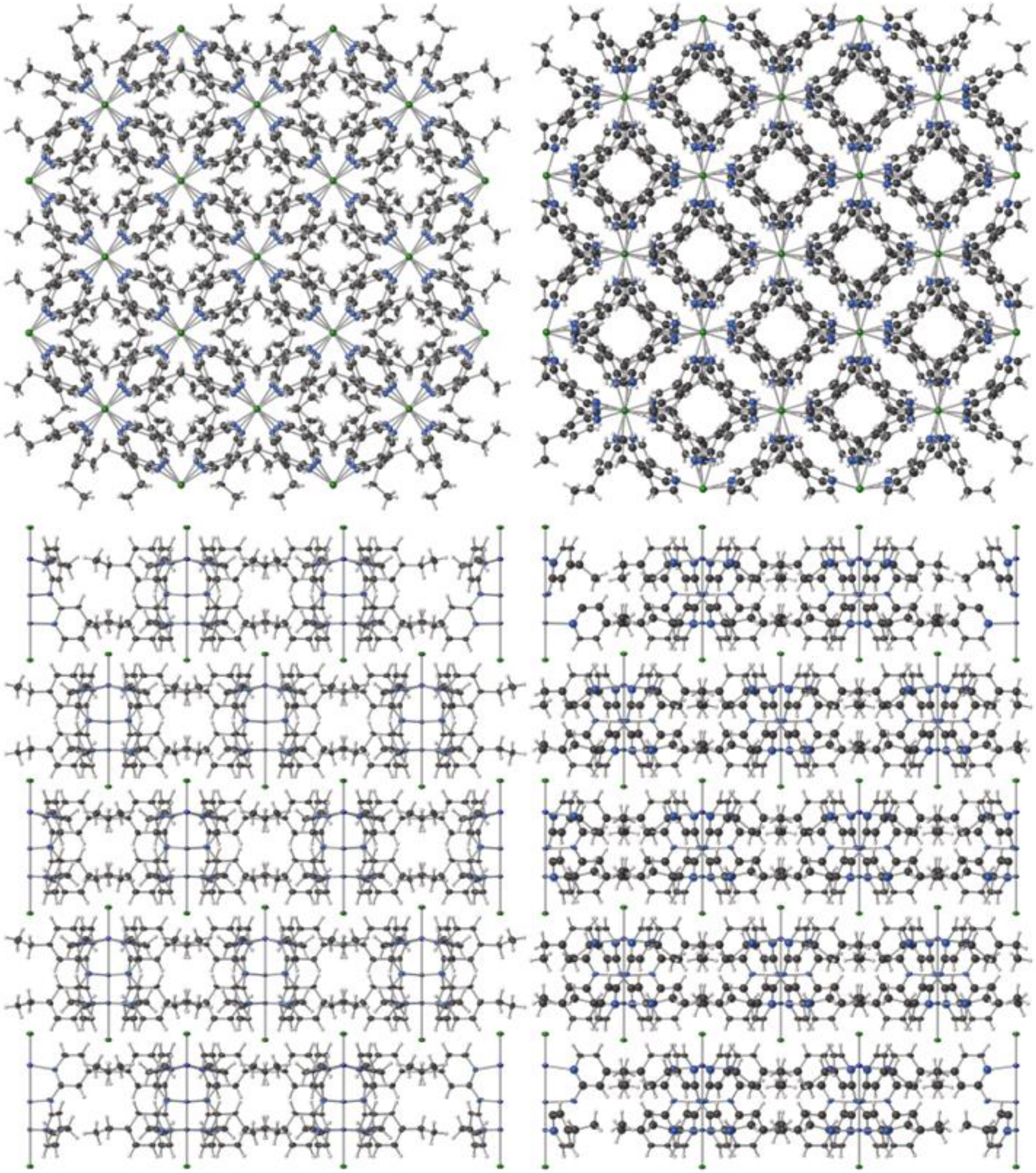

Figure S49: Packing diagrams of **4** showing the major (left) and minor (right) ligand components along the 001 (top) and 100 (bottom) views. Ellipsoids are drawn at 50% probability.

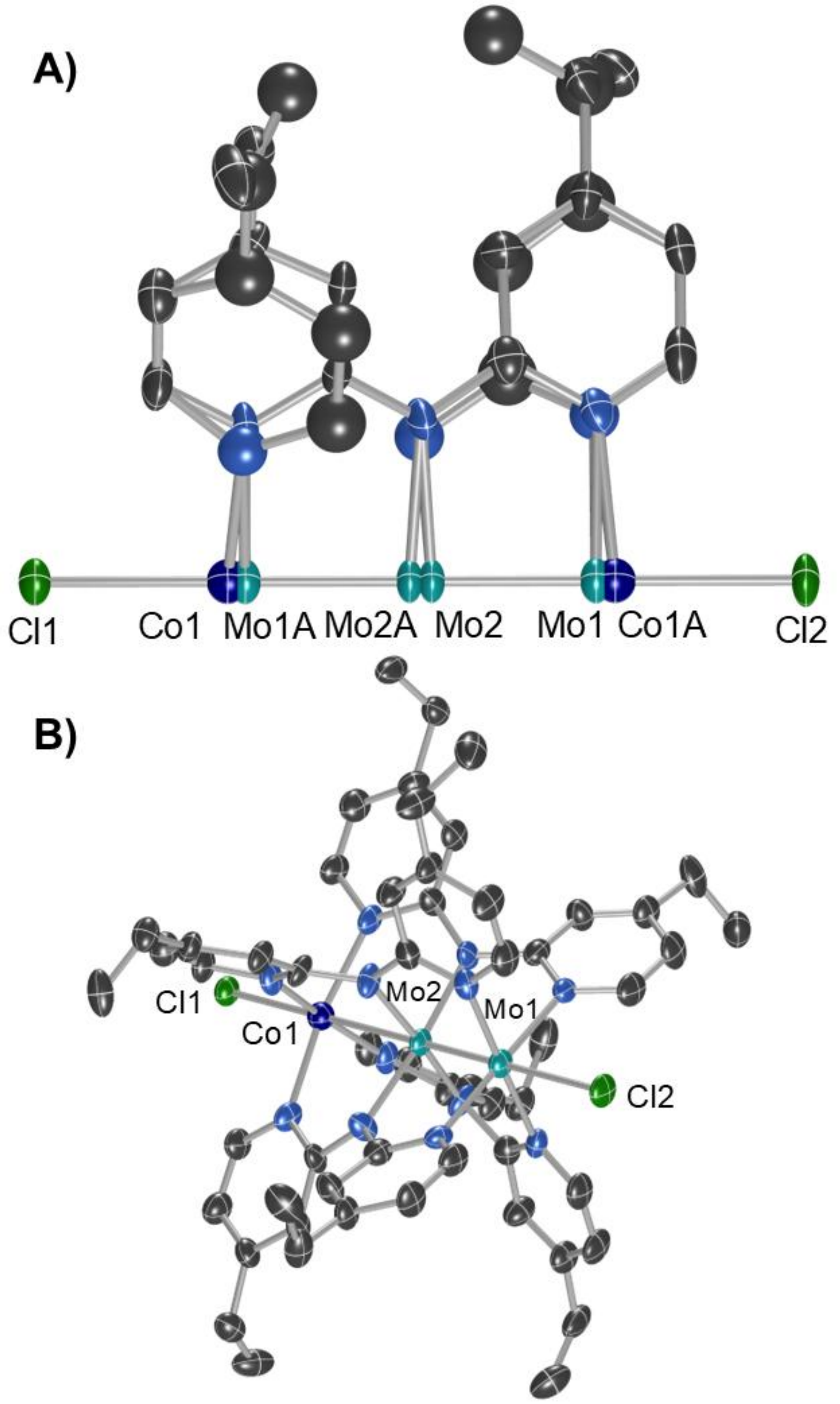

Figure S50: A shows the asymmetric unit of **5**, showing disorder in both the metal atom positions and ligand positions. The metal atom components are split almost 50:50, with the major component having an occupancy of 53.3(13)% while the ligands have a preference for the rho isomer with the major component having an occupancy of 80.1(5)%. B shows a drawing of the full structure of **5** with only the major components of the structure shown. Ellipsoids are drawn at 50% probability and hydrogen atoms are omitted for clarity.

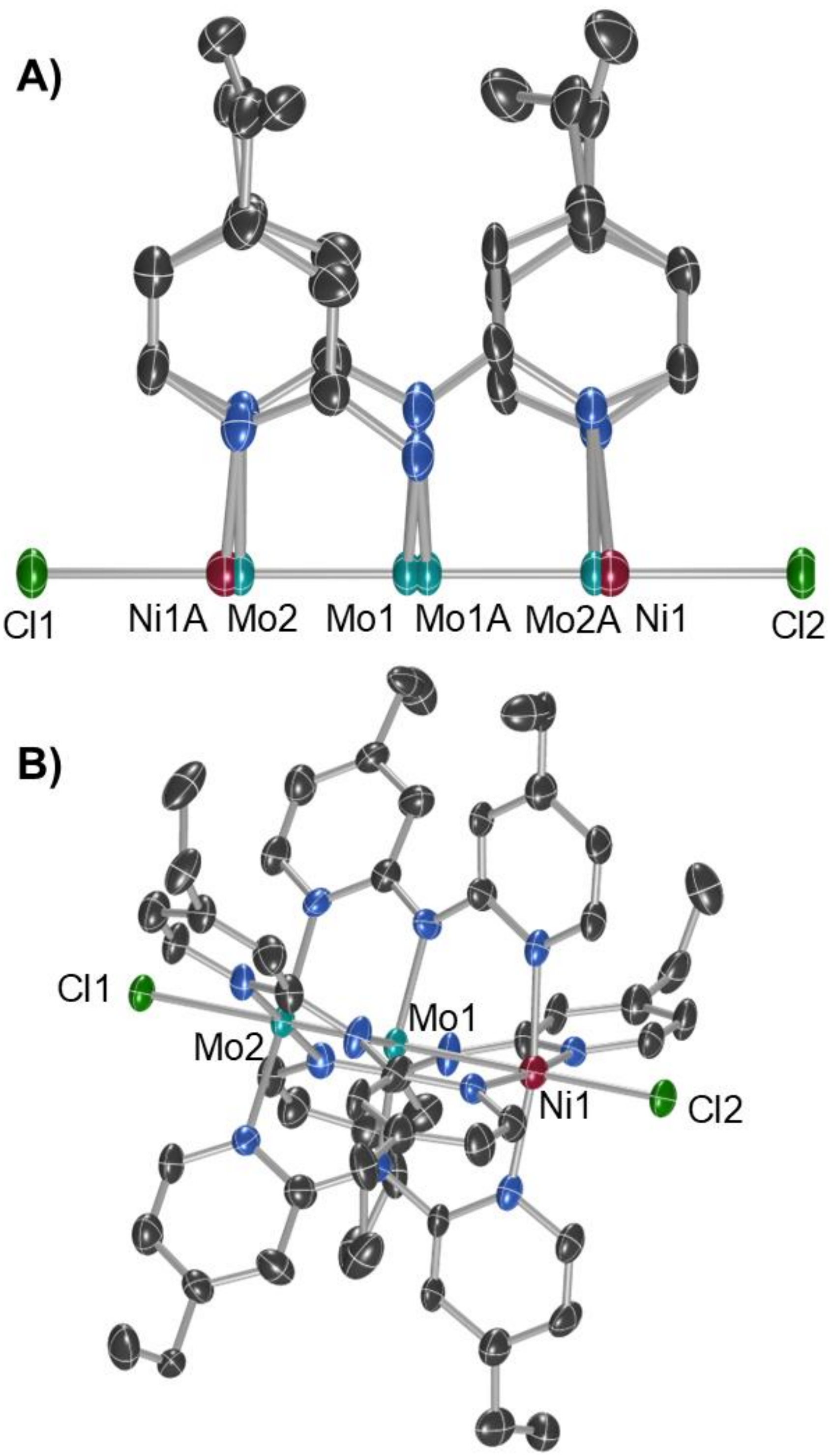

Figure S51: A shows the asymmetric unit of **6**, showing disorder in both the metal atom positions and ligand positions. The metal atom components are split with the major component having an occupancy of 60.9(9)% while the ligands have a preference for the rho isomer with the major component having an occupancy of 83.0(4)%. B shows a drawing of the full structure of **6** with only the major components of the structure shown. Ellipsoids are drawn at 50% probability and hydrogen atoms are omitted for clarity.

TD-DFT Results for 1-6:

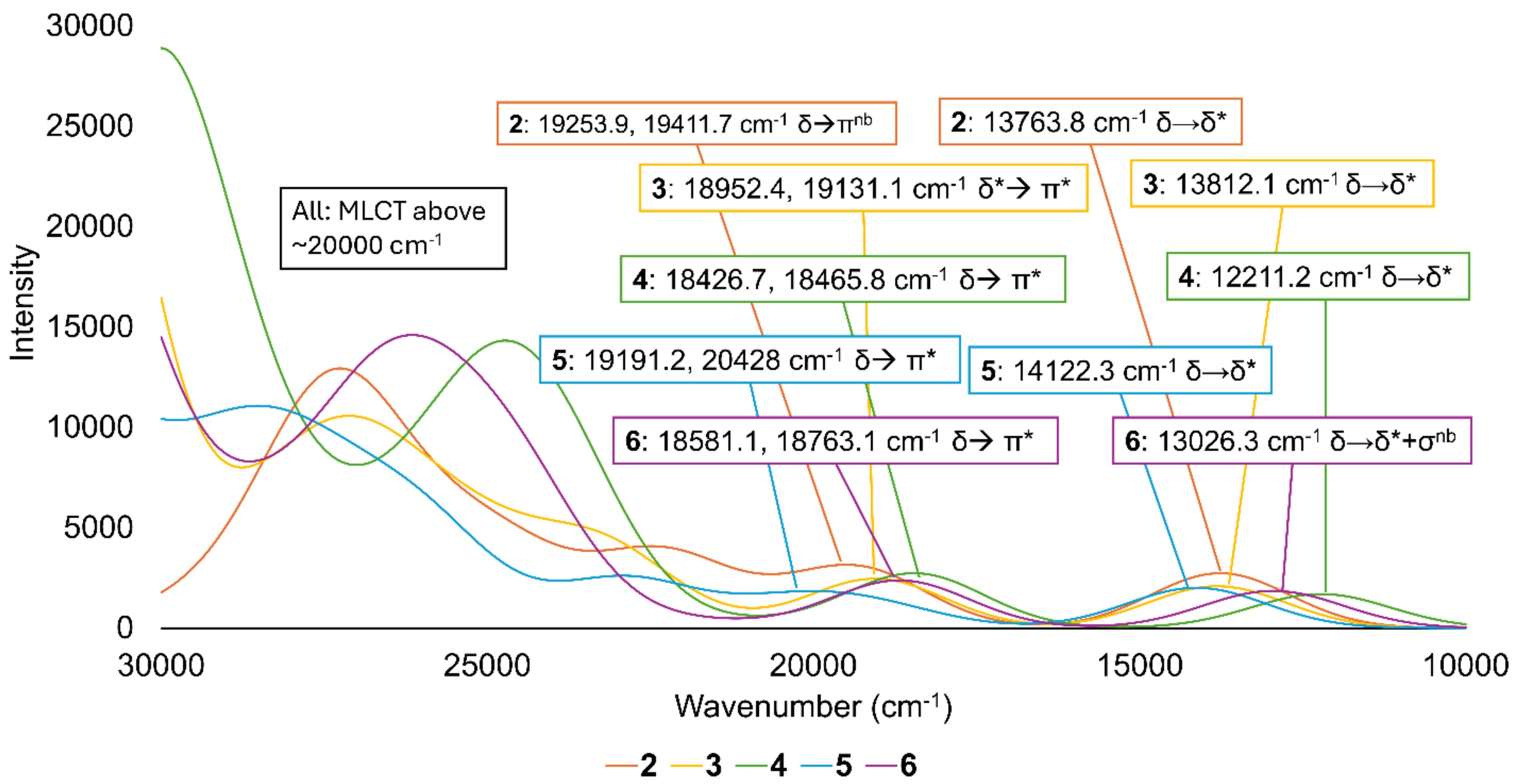

Figure S52: Calculated TD-DFT spectra for **2-6**, with labels indicating the primary orbital composition and calculated energy in cm^{-1} of each transition of interest

Calculated EDDMs by Compound and Absolute Energies (Eh):
2: **$Mo_2Cr(dedpa)_4Cl_2$:** -12745.48394334 Eh

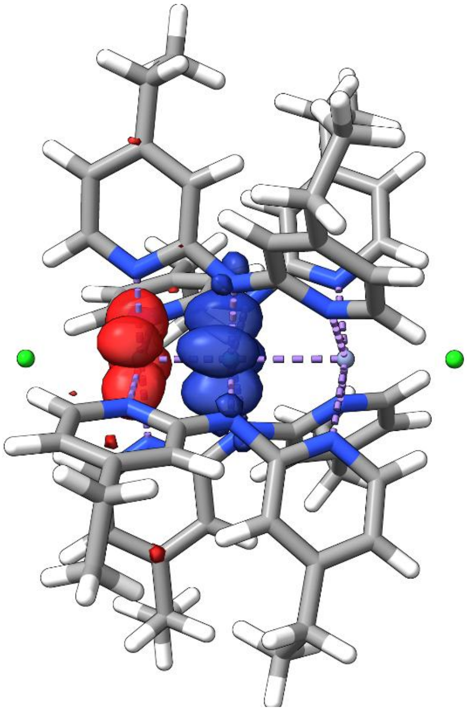

Figure S53: Calculated EDDM of the δ to δ* transition of **2**, at 13764 cm^{-1}

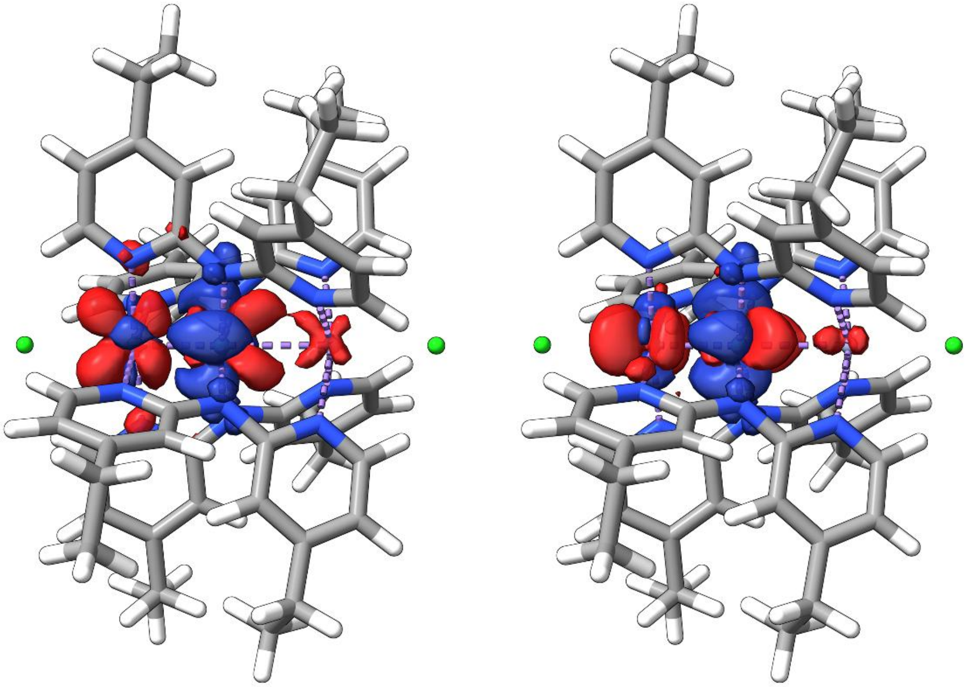

Figure S54: Calculated EDDMs of the δ to π* transition of **2**, at 19253.9, 19411.7 cm^{-1}

3: $Mo_2Mn(dedpa)_4Cl_2$: -12851.96871660 Eh

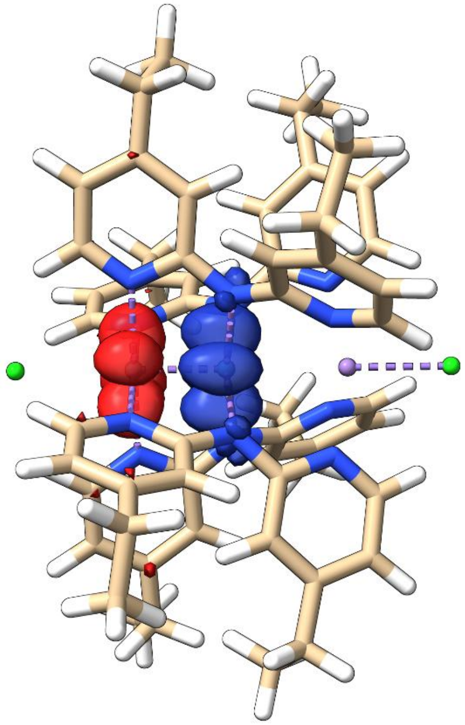

Figure S55: Calculated EDDM of the δ to δ* transition of **3**, at 13812 cm^{-1}

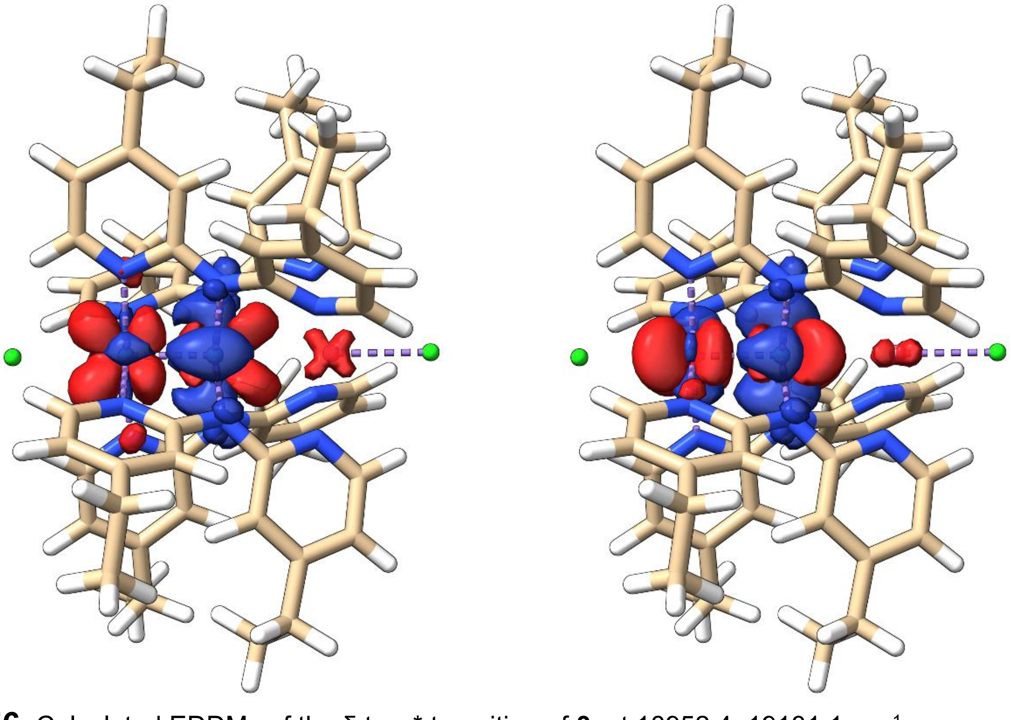

Figure S56: Calculated EDDMs of the δ to π* transition of **3**, at 18952.4, 19131.1 cm^{-1}

4: $Mo_2Fe(dedpa)_4Cl_2$: -12964.61696266 Eh

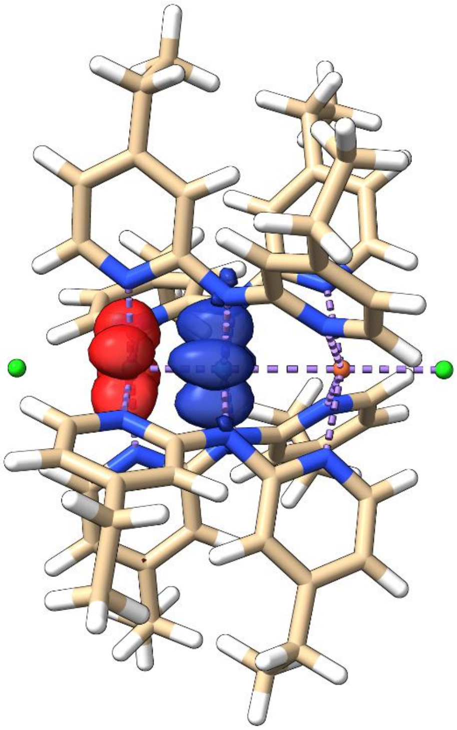

Figure S57: Calculated EDDM of the δ to δ* transition of **4**, at 12211.2 cm^{-1}

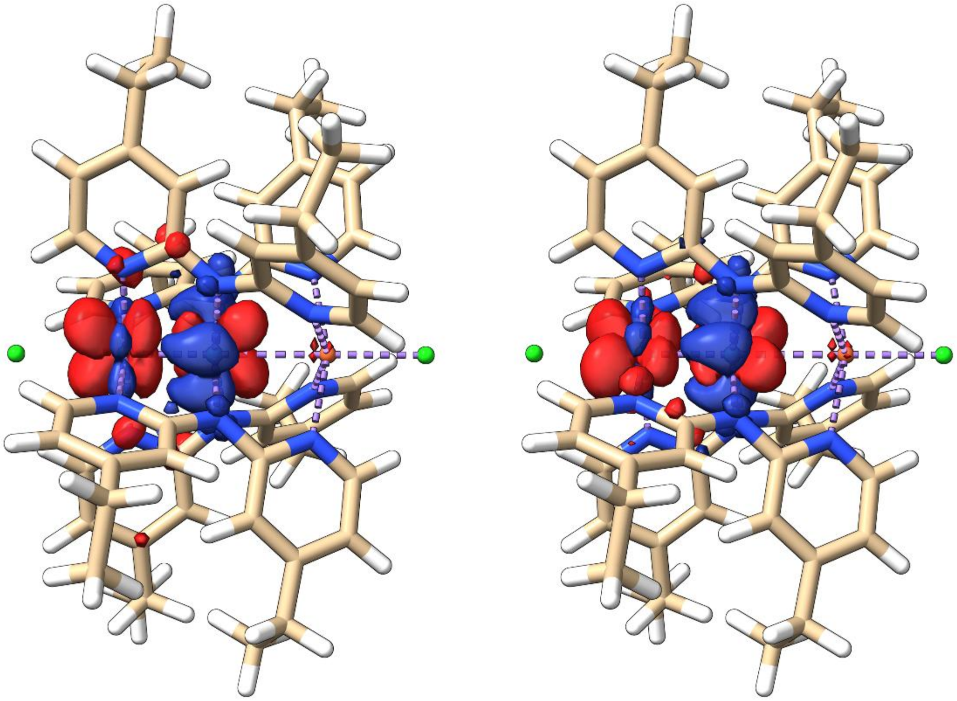

Figure S58: Calculated EDDMs of the δ to π* transition of **4**, at 18426.7, 18465.8 cm^{-1}

5: $Mo_2Co(dedpa)_4Cl_2$: -13083.61414725 Eh

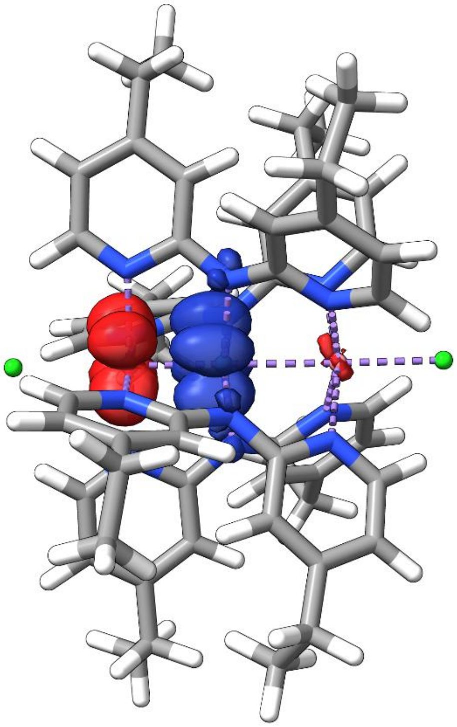

Figure S59: Calculated EDDM of the δ to δ* transition of **5**, at 14122.3 cm^{-1}

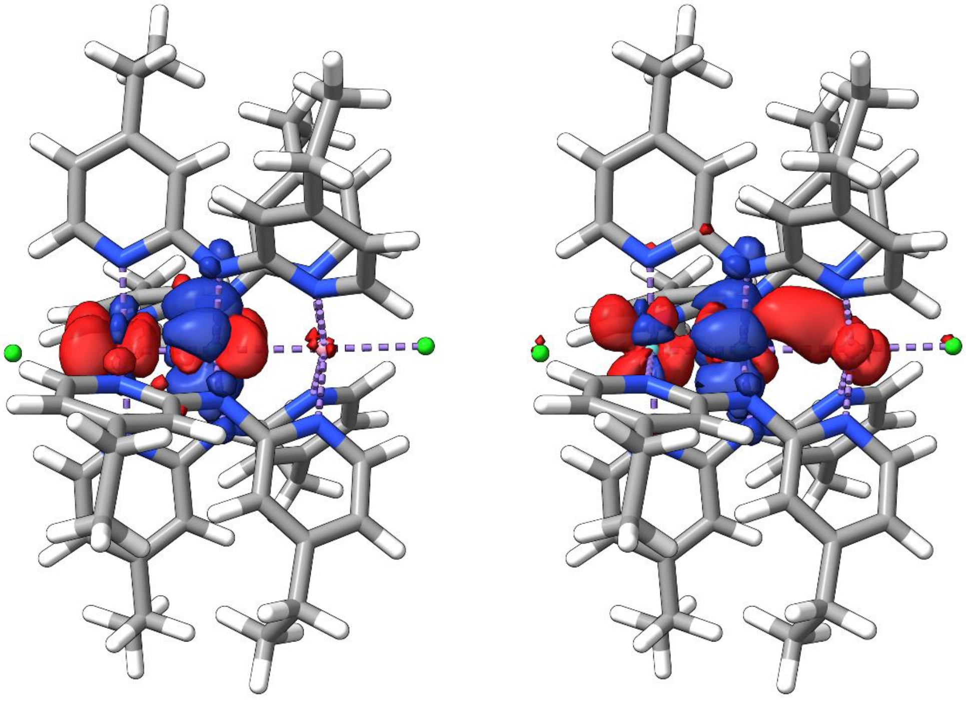

Figure S60: Calculated EDDMs of the δ to π* transition of **5**, at 19191.2, 20428 cm^{-1}

6: $Mo_2Ni(dedpa)_4Cl_2$: -13209.10220495 Eh

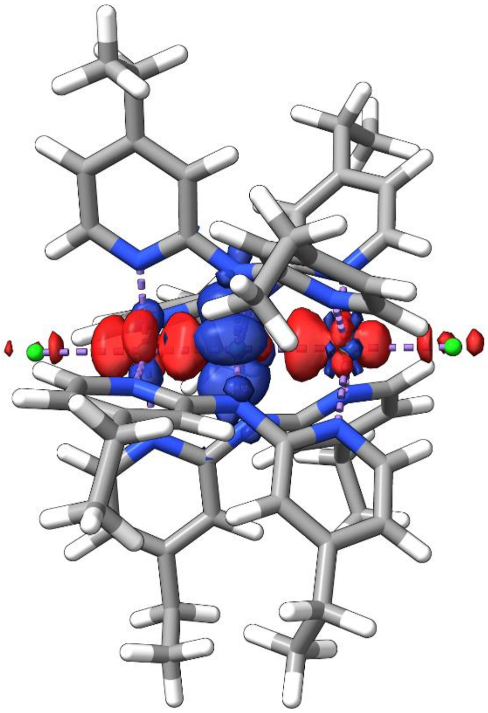

Figure S61: Calculated EDDM of the δ to δ* transition of **6**, at 13026.3 cm^{-1}

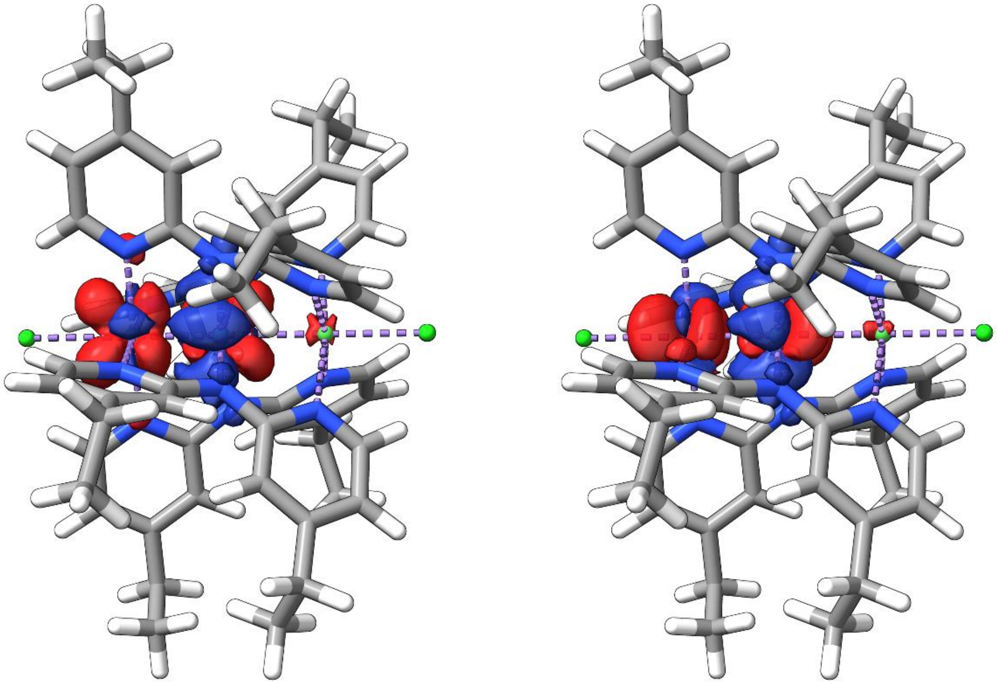

Figure S62: Calculated EDDMs of the δ to π* transition of **6**, at 18581.1, 18763.1 cm^{-1}

References:

[1] M. Nippe, J. Wang, E. Bill, H. Hope, N. S. Dalal, J. F. Berry, Crystals in Which Some Metal Atoms are More Equal Than Others: Inequalities From Crystal Packing and Their Spectroscopic/Magnetic Consequences *J. Am. Chem. Soc.* **2010**, *132*, 14261-14272, 10.1021/ja106510g.

[2] T. Bender, P. von Zezschwitz, Total Synthesis of 4-Acetyl-1,3-Dihydroimidazo[4,5-c]Pyridin-2-One, a New Microbial Metabolite from a Streptomyces Species *Nat. Prod. Commun.* **2009**, *4*, 1934578X0900400719, 10.1177/1934578X0900400719.

[3] V. Bonnet, F. Mongin, F. Trécourt, G. Quéguiner, P. Knochel, Syntheses of substituted pyridines, quinolines and diazines via palladium-catalyzed cross-coupling of aryl Grignard reagents *Tetrahedron* **2002**, *58*, 4429-4438, 10.1016/S0040-4020(02)00411-8.

[4] F. H. Case, T. J. Kasper, The Preparation of Some Substituted 2,6-Bis-(2-pyridyl)-pyridines *J. Am. Chem. Soc.* **1956**, *78*, 5842-5844, 10.1021/ja01603a036.

[5] S. Stoll, A. Schweiger, EasySpin, a comprehensive software package for spectral simulation and analysis in EPR *J. Magn. Reson.* **2006**, *178*, 42-55, 10.1016/j.jmr.2005.08.013.

[6] G. A. Bain, J. F. Berry, Diamagnetic Corrections and Pascal's Constants *J. Chem. Educ.* **2008**, *85*, 532, 10.1021/ed085p532.

[7] N. F. Chilton, R. P. Anderson, L. D. Turner, A. Soncini, K. S. Murray, PHI: A powerful new program for the analysis of anisotropic monomeric and exchange-coupled polynuclear d- and f-block complexes *J. Comput. Chem.* **2013**, *34*, 1164-1175, 10.1002/jcc.23234.

[8] I. Prisecaru, WMOSS4 Mossbauer spectral analysis software, **2009.**

[9] J. E. Dennis, D. M. Gay, R. E. Walsh, An Adaptive Nonlinear Least-Squares Algorithm *ACM Trans. Math. Softw.* **1981**, *7*, 348–368, 10.1145/355958.355965.

[10] F. Neese, Software update: The ORCA program system—Version 5.0 *Wiley Interdiscip. Rev. Comput. Mol. Sci.* **2022**, *12*, e1606, 10.1002/wcms.1606.

[11] M. Garcia-Ratés, F. Neese, Effect of the Solute Cavity on the Solvation Energy and its Derivatives within the Framework of the Gaussian Charge Scheme *J. Comput. Chem.* **2020**, *41*, 922-939, 10.1002/jcc.26139.

[12] F. Neese, An improvement of the resolution of the identity approximation for the formation of the Coulomb matrix *J. Comput. Chem.* **2003**, *24*, 1740-1747, 10.1002/jcc.10318.

[13] F. Neese, The SHARK integral generation and digestion system *J. Comput. Chem.* **2023**, *44*, 381-396, 10.1002/jcc.26942.

[14] D. A. Pantazis, X.-Y. Chen, C. R. Landis, F. Neese, All-Electron Scalar Relativistic Basis Sets for Third-Row Transition Metal Atoms *J. Chem. Theory Comput.* **2008**, *4*, 908-919, 10.1021/ct800047t.

[15] S. Grimme, J. Antony, S. Ehrlich, H. Krieg, A consistent and accurate ab initio parametrization of density functional dispersion correction (DFT-D) for the 94 elements H-Pu *J. Chem. Phys.* **2010**, *132*, 154104, 10.1063/1.3382344.

[16] S. Grimme, S. Ehrlich, L. Goerigk, Effect of the damping function in dispersion corrected density functional theory *J. Comput. Chem.* **2011**, *32*, 1456-1465, 10.1002/jcc.21759.

[17] F. Weigend, Accurate Coulomb-fitting basis sets for H to Rn *Phys. Chem. Chem. Phys.* **2006**, *8*, 1057-1065, 10.1039/B515623H.

[18] F. Weigend, R. Ahlrichs, Balanced basis sets of split valence, triple zeta valence and quadruple zeta valence quality for H to Rn: Design and assessment of accuracy *Phys. Chem. Chem. Phys.* **2005**, *7*, 3297-3305, 10.1039/B508541A.

[19] J. P. Perdew, K. Burke, M. Ernzerhof, Generalized Gradient Approximation Made Simple *Phys. Rev. Lett.* **1996**, *77*, 3865-3868, 10.1103/PhysRevLett.77.3865.

[20] C. Lee, W. Yang, R. G. Parr, Development of the Colle-Salvetti correlation-energy formula into a functional of the electron density *Phys. Rev. B: Condens. Matter* **1988**, *37*, 785-789, 10.1103/PhysRevB.37.785.

[21] P. J. Stephens, F. J. Devlin, C. F. Chabalowski, M. J. Frisch, Ab initio calculation of vibrational absorption and circular dichroism spectra using density functional force fields *J. Phys. Chem.* **1994**, *98*, 11623-11627,

[22] S. H. Vosko, L. Wilk, M. Nusair, Accurate spin-dependent electron liquid correlation energies for local spin density calculations: a critical analysis *Can. J. Phys.* **1980**, *58*, 1200-1211, 10.1139/p80-159.

[23] A. D. Becke, Density-functional thermochemistry. III. The role of exact exchange *J. Chem. Phys.* **1993**, *98*, 5648-5652, 10.1063/1.464913.
[24] F. Neese, Prediction and interpretation of the ^{57}Fe isomer shift in Mössbauer spectra by density functional theory *Inorg. Chim. Acta* **2002**, *337*, 181-192, 10.1016/S0020-1693(02)01031-9.
[25] M. Römelt, S. Ye, F. Neese, Calibration of Modern Density Functional Theory Methods for the Prediction of ^{57}Fe Mössbauer Isomer Shifts: Meta-GGA and Double-Hybrid Functionals *Inorg. Chem.* **2009**, *48*, 784-785, 10.1021/ic801535v.
[26] S. Sinnecker, L. D. Slep, E. Bill, F. Neese, Performance of Nonrelativistic and Quasi-Relativistic Hybrid DFT for the Prediction of Electric and Magnetic Hyperfine Parameters in ^{57}Fe Mössbauer Spectra *Inorg. Chem.* **2005**, *44*, 2245-2254, 10.1021/ic048609e.
[27] T. Yanai, D. P. Tew, N. C. Handy, A new hybrid exchange–correlation functional using the Coulomb-attenuating method (CAM-B3LYP) *Chem. Phys. Lett.* **2004**, *393*, 51-57, 10.1016/j.cplett.2004.06.011.
[28] P.-O. Löwdin, *Advances in quantum chemistry, Vol. 11*, Academic Press, **1979**.
[29] P. O. Löwdin, On the Non-Orthogonality Problem Connected with the Use of Atomic Wave Functions in the Theory of Molecules and Crystals *J. Chem. Phys.* **1950**, *18*, 365-375, 10.1063/1.1747632.
[30] T. D. Goddard, C. C. Huang, E. C. Meng, E. F. Pettersen, G. S. Couch, J. H. Morris, T. E. Ferrin, UCSF ChimeraX: Meeting modern challenges in visualization and analysis *Protein Sci.* **2018**, *27*, 14-25, 10.1002/pro.3235.
[31] E. C. Meng, T. D. Goddard, E. F. Pettersen, G. S. Couch, Z. J. Pearson, J. H. Morris, T. E. Ferrin, UCSF ChimeraX: Tools for structure building and analysis *Protein Sci.* **2023**, *32*, e4792, 10.1002/pro.4792.
[32] E. F. Pettersen, T. D. Goddard, C. C. Huang, E. C. Meng, G. S. Couch, T. I. Croll, J. H. Morris, T. E. Ferrin, UCSF ChimeraX: Structure visualization for researchers, educators, and developers *Protein Sci.* **2021**, *30*, 70-82, 10.1002/pro.3943.
[33] APEX4, 2022.1-1, Bruker-AXS, Madison, Wisconsin, USA, **2022.**
[34] F. Kleemiss, O. V. Dolomanov, M. Bodensteiner, N. Peyerimhoff, L. Midgley, L. J. Bourhis, A. Genoni, L. A. Malaspina, D. Jayatilaka, J. L. Spencer, F. White, B. Grundkötter-Stock, S. Steinhauer, D. Lentz, H. Puschmann, S. Grabowsky, Accurate crystal structures and chemical properties from NoSpherA2 *Chem. Sci.* **2021**, *12*, 1675-1692, 10.1039/D0SC05526C.
[35] L. Krause, R. Herbst-Irmer, G. M. Sheldrick, D. Stalke, Comparison of silver and molybdenum microfocus X-ray sources for single-crystal structure determination *J. Appl. Crystallogr.* **2015**, *48*, 3-10, 10.1107/S1600576714022985.
[36] G. Sheldrick, SHELXT - Integrated space-group and crystal-structure determination *Acta Crystallogr. Sect. A* **2015**, *71*, 3-8, 10.1107/S2053273314026370.
[37] G. Sheldrick, Crystal structure refinement with SHELXL *Acta Crystallogr. Sect. C* **2015**, *71*, 3-8, 10.1107/S2053229614024218.
[38] A. Spek, PLATON SQUEEZE: a tool for the calculation of the disordered solvent contribution to the calculated structure factors *Acta Crystallogr. Sect. C* **2015**, *71*, 9-18, doi:10.1107/S2053229614024929.
[39] M. Elie, F. Sguerra, F. Di Meo, M. D. Weber, R. Marion, A. Grimault, J.-F. Lohier, A. Stallivieri, A. Brosseau, R. B. Pansu, J.-L. Renaud, M. Linares, M. Hamel, R. D. Costa, S. Gaillard, Designing NHC–Copper(I) Dipyridylamine Complexes for Blue Light-Emitting Electrochemical Cells *ACS Appl. Mater. Interfaces* **2016**, *8*, 14678-14691, 10.1021/acsami.6b04647.
[40] R. Marion, F. Sguerra, F. Di Meo, E. Sauvageot, J.-F. Lohier, R. Daniellou, J.-L. Renaud, M. Linares, M. Hamel, S. Gaillard, NHC Copper(I) Complexes Bearing Dipyridylamine Ligands: Synthesis, Structural, and Photoluminescent Studies *Inorg. Chem.* **2014**, *53*, 9181-9191, 10.1021/ic501230m.
[41] D. W. Brogden, J. H. Christian, N. S. Dalal, J. F. Berry, Completing the series of Group VI heterotrimetallic $M_2Cr(dpa)_4Cl_2$ (M_2=Cr_2, Mo_2, MoW and W_2) compounds and investigating their metal–metal interactions using density functional theory *Inorg. Chim. Acta* **2015**, *424*, 241-247, 10.1016/j.ica.2014.08.020.
[42] M. Nippe, E. Victor, J. F. Berry, Do Metal–Metal Multiply-Bonded “Ligands” Have a trans Influence? Structural and Magnetic Comparisons of Heterometallic Cr≣Cr⋯Co and Mo≣Mo⋯Co Interactions *Eur. J. Inorg. Chem.* **2008**, 5569-5572, 10.1002/ejic.200801001.

[43] J. A. Chipman, J. F. Berry, Extraordinarily Large Ferromagnetic Coupling ($J \geq 150\ cm^{-1}$) by Electron Delocalization in a Heterometallic Mo≣Mo−Ni Chain Complex *Chem. Eur. J.* **2018**, *24*, 1494-1499, 10.1002/chem.201704588.
